# Genome-wide association study of multisite chronic pain in UK Biobank

**DOI:** 10.1371/journal.pgen.1008164

**Published:** 2019-06-13

**Authors:** Keira J. A. Johnston, Mark J. Adams, Barbara I. Nicholl, Joey Ward, Rona J. Strawbridge, Amy Ferguson, Andrew M. McIntosh, Mark E. S. Bailey, Daniel J. Smith

**Affiliations:** 1 Institute of Health and Wellbeing, University of Glasgow, Scotland, United Kingdom; 2 Deanery of Molecular, Genetic and Population Health Sciences, College of Medicine and Veterinary Medicine, University of Edinburgh, Scotland, United Kingdom; 3 School of Life Sciences, College of Medical, Veterinary & Life Sciences, University of Glasgow, Scotland, United Kingdom; 4 Centre for Cognitive Ageing and Cognitive Epidemiology, University of Edinburgh, Scotland, United Kingdom; 5 Department of Medicine Solna, Karolinska Institute, Stockholm, Sweden; Case Western Reserve University, UNITED STATES

## Abstract

Chronic pain is highly prevalent worldwide and represents a significant socioeconomic and public health burden. Several aspects of chronic pain, for example back pain and a severity-related phenotype ‘chronic pain grade’, have been shown previously to be complex heritable traits with a polygenic component. Additional pain-related phenotypes capturing aspects of an individual’s overall sensitivity to experiencing and reporting chronic pain have also been suggested as a focus for investigation. We made use of a measure of the number of sites of chronic pain in individuals within the UK general population. This measure, termed Multisite Chronic Pain (MCP), is a complex trait and its genetic architecture has not previously been investigated. To address this, we carried out a large-scale genome-wide association study (GWAS) of MCP in ~380,000 UK Biobank participants. Our findings were consistent with MCP having a significant polygenic component, with a Single Nucleotide Polymorphism (SNP) heritability of 10.2%. In total 76 independent lead SNPs at 39 risk loci were associated with MCP. Additional gene-level association analyses identified neurogenesis, synaptic plasticity, nervous system development, cell-cycle progression and apoptosis genes as enriched for genetic association with MCP. Genetic correlations were observed between MCP and a range of psychiatric, autoimmune and anthropometric traits, including major depressive disorder (MDD), asthma and Body Mass Index (BMI). Furthermore, in Mendelian randomisation (MR) analyses a causal effect of MCP on MDD was observed. Additionally, a polygenic risk score (PRS) for MCP was found to significantly predict chronic widespread pain (pain all over the body), indicating the existence of genetic variants contributing to both of these pain phenotypes. Overall, our findings support the proposition that chronic pain involves a strong nervous system component with implications for our understanding of the physiology of chronic pain. These discoveries may also inform the future development of novel treatment approaches.

## Author summary

Chronic pain is common worldwide and imposes a significant burden from a public health and socioeconomic perspective. The reasons why some individuals develop chronic pain and others do not are not fully understood. In this study we searched for genetic variants associated with chronic pain in a large general-population cohort. We also assessed how this genetic variation was correlated with a range of other diseases and traits, such as depression and BMI, and we tested for causal relationships between depression and chronic pain. We found that chronic pain was associated with several genes involved in brain function and development and was correlated with mental health and autoimmune traits (including depression, PTSD and asthma). We also found evidence for causal relationships between chronic pain and major depressive disorder. This work provides new insights into the genetics and underlying biology of chronic pain and may help to inform new treatment strategies.

## Introduction

Chronic pain, conventionally defined as pain lasting longer than 3 months, has high global prevalence (~30%; [1]), imposes a significant socioeconomic burden, and contributes to excess mortality [2,3]. It is often associated with both specific and non-specific medical conditions such as cancers, HIV/AIDS, fibromyalgia and musculoskeletal conditions [4–6], and can be classified according to different grading systems, such as the Von Korff chronic pain grade [7]. Several aspects of chronic pain, such as chronic pain grade and back pain, have been studied from the genetic point of view, and several have been shown to be complex traits with moderate heritability [3,8]. In part due to the heterogeneity of pain assessment and pain experience, there are very few large-scale genetic studies of chronic pain and no genome-wide significant genetic variants have yet been identified [9,10].

Chronic pain and chronic pain disorders are often comorbid with psychiatric and neurodevelopmental disorders, including Major Depressive Disorder (MDD) [11]. The immune and nervous systems play a central role in chronic pain development and maintenance [12,13]. Similarly, obesity and chronic pain are often comorbid, with extrinsic factors such as sleep disturbance also impacting on chronic pain [14,15]. Altered sleep quality and reduced circadian rhythmicity are also common in those with chronic pain [16]. Chronic pain is also a common component of many neurological diseases [17].

The relationship between injury and other peripheral insult, consequent acute pain and the subsequent development of chronic pain has not been fully explained. Not everyone who undergoes major surgery or is badly injured will develop chronic pain, for example [18], and the degree of joint damage in osteoarthritis is not related to chronic pain severity [19]. Conversely, Complex Regional Pain Syndrome (CRPS) can be incited by minor peripheral insult such as insertion of a needle (reviewed by Denk, McMahon and Tracey, 2014). Structural and functional changes in the brain and spinal cord are associated with the development and maintenance of chronic pain, and affective brain regions are involved in chronic pain perception (this is in contrast to acute pain and even to prolonged acute pain experience) [20–24]. It is also unlikely that there are legitimate cut-off points or thresholds for localised and widespread chronic pain, with pain instead existing on a “continuum of widespreadness” [25]. It may, therefore, be more valuable and powerful to examine measures of chronic pain as complex neuropathological traits in themselves, rather than just to study disorders and conditions with chronic pain as a main feature or pain experienced only in specific bodily locations. Our aim in this study was predicated on the idea that predisposing biological processes might influence how many sites are affected in individuals that experience any chronic pain, and we carried out a genome-wide association study of number of chronic pain sites to look for predisposing loci, assess the degree of genetic overlap with related traits and disorders and generate insights into the genetic architecture of chronic pain.

## Results

### Genome-wide association study

**Fig 1:**
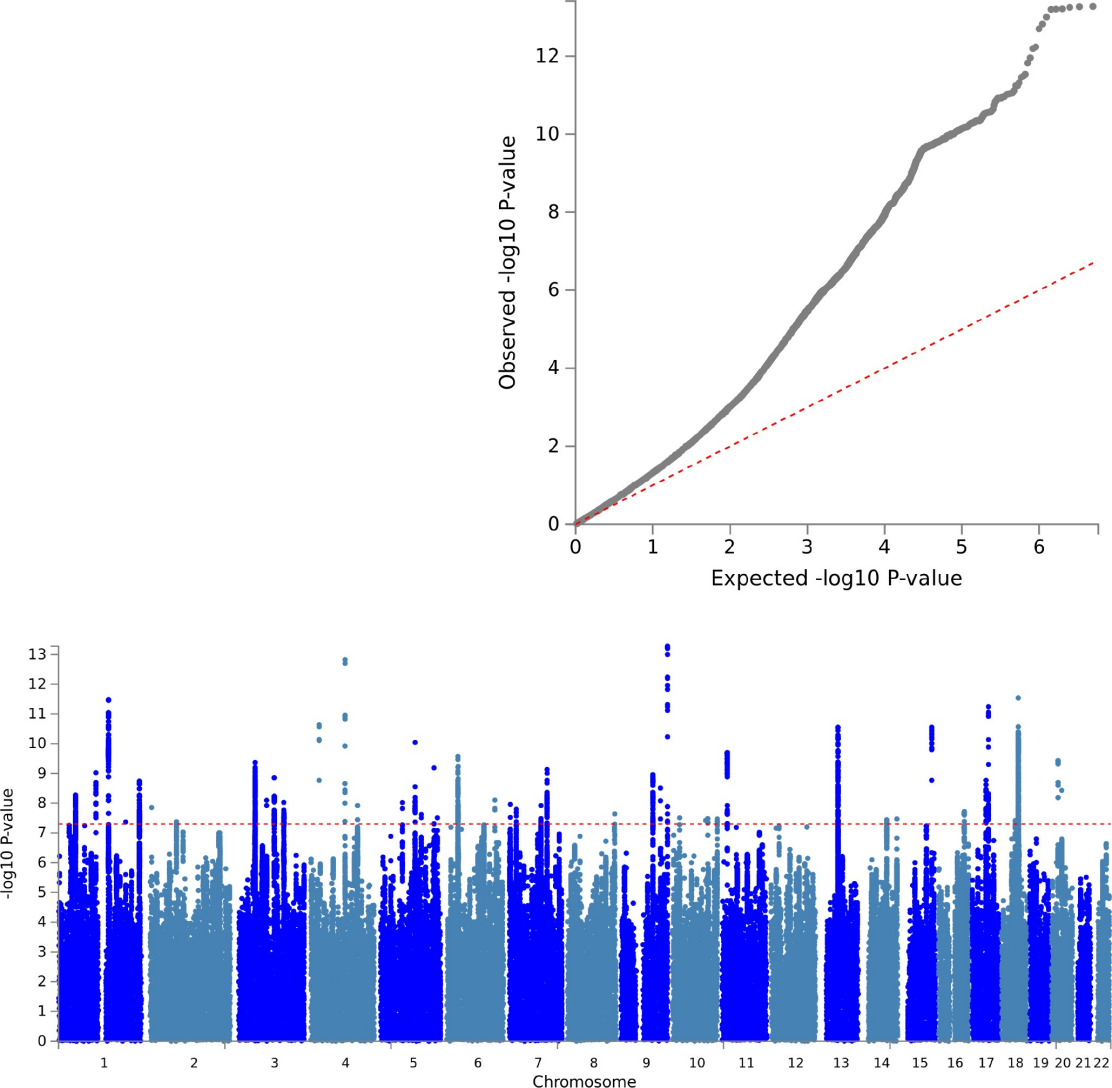
Manhattan plot & QQ plot for MCP GWAS. **A:** SNP associations across chromosomes 1–22 are displayed. Genome-wide significance (a p value of 5 x 10^−8^, ~ 7.3 on the -log10 scale) is indicated by the dashed red line. **B**: Observed versus expected GWAS p values on the -log10 scale are shown.

To identify genetic risk loci influencing Multisite Chronic Pain (MCP), we performed a GWAS with adjustment for age, sex and genotyping array using BOLT-LMM (see Methods). No evidence was found for inflation of the test statistics due to hidden population stratification (λ_GC_ = 1.26; after adjustment for sample size λ_GC_1000 = 1.001). LD-score regression (LDSR) analysis was consistent with a polygenic contribution to MCP (LDSR intercept = 1.0249, SE 0.0274; Fig 1) [26] and yielded a Single Nucleotide Polymorphism (SNP) heritability estimate of 10.2%. BOLT-LMM gave a similar SNP heritability estimate (pseudo-h^2^ = 10.3%). In total, 1, 748 SNPs associated with MCP level at genome-wide significance (p < 5 x 10^−8^) were identified. Conditional analysis of the association signals at each locus revealed 76 independent genome-wide significant lead SNPs across 39 risk loci located on chromosomes 1–11, 13–18 and 20 (Table 1). Sensitivity analysis additionally adjusting for BMI did not significantly alter these association analysis results.

**Table 1:** Genomic risk loci.

Genomic Locus	rsID	chr	pos	Nearest Gene	A1	A2	MAF	r2	beta	se	P
1	rs10888692	1	50991473	FAF1	C	G	0.4301	1	-0.0143	0.0025	5.30E-09
2	rs197422	1	112000000	KCND3	C	A	0.3794	1	-0.015	0.0025	2.00E-09
3	rs59898460	1	150000000	LINC00568	T	C	0.4044	1	0.0169	0.0025	9.20E-12
4	rs12071912	1	243000000	RP11-261C10.3	C	T	0.3163	1	-0.0153	0.0026	5.30E-09
5	rs4852567	2	80703379	CTNNA2	A	G	0.2834	1	0.0149	0.0027	4.30E-08
6	rs7628207	3	49754970	RNF123:AMIGO3:GMPPB	T	C	0.1766	1	0.0195	0.0032	8.40E-10
7	rs28428925	3	107000000	BBX	G	A	0.1365	1	-0.0214	0.0035	1.40E-09
8	rs6770476	3	136000000	STAG1	C	T	0.289	1	-0.0154	0.0027	9.40E-09
9	rs34811474	4	25408838	ANAPC4	G	A	0.2285	1	0.0192	0.0029	2.70E-11
10	rs13135092	4	103000000	SLC39A8	A	G	0.08071	1	-0.0328	0.0044	1.50E-13
11	rs13136239	4	141000000	MAML3	G	A	0.3508	1	0.0141	0.0026	3.60E-08
12	rs6869446	5	65570607	RP11-305P14.1	T	C	0.3861	1	-0.0144	0.0025	9.50E-09
13	rs1976423	5	104000000	RP11-6N13.1	A	C	0.4968	1	-0.014	0.0024	8.20E-09
14	rs17474406	5	123000000	CEP120	G	A	0.01805	1	-0.0492	0.0088	2.40E-08
15	rs1946247	5	161000000	GABRB2	T	G	0.1389	1	-0.019	0.0035	4.90E-08
16	rs11751591	6	33794215	MLN	G	A	0.1516	1	0.0214	0.0034	2.70E-10
17	rs6907508	6	34592090	C6orf106	A	G	0.1146	1	-0.0217	0.0038	1.10E-08
18	rs6926377	6	145000000	UTRN	A	C	0.294	1	-0.0155	0.0027	7.90E-09
19	rs10259354	7	3487414	SDK1	G	A	0.2983	1	0.0147	0.0026	3.00E-08
20	rs7798894	7	21552995	SP4	A	T	0.2888	1	0.0153	0.0027	1.60E-08
21	rs6966540	7	95727967	DYNC1I1	T	C	0.3762	1	-0.0139	0.0025	3.30E-08
22	rs12537376	7	114000000	FOXP2	A	G	0.3969	1	0.0151	0.0025	1.70E-09
23	rs11786084	8	143000000	AC138647.1	G	A	0.3328	1	-0.0145	0.0026	2.30E-08
24	rs10992729	9	96181075	Y_RNA	C	T	0.3344	1	0.0158	0.0026	1.10E-09
25	rs6478241	9	119000000	ASTN2	A	G	0.365	1	0.0149	0.0025	3.10E-09
26	9:140251458_G_A	9	140000000	EXD3	G	A	0.123	1	-0.0277	0.0037	5.30E-14
27	rs2183271	10	21957229	MLLT10	T	C	0.3578	1	-0.014	0.0025	3.10E-08
28	rs11599236	10	106000000	SORCS3	T	C	0.4058	1	0.0138	0.0025	3.30E-08
29	rs12765185	10	135000000	KNDC1	T	A	0.2669	1	-0.0151	0.0027	3.90E-08
30	rs61883178	11	16317779	SOX6	C	A	0.1696	1	-0.0208	0.0033	2.00E-10
31	rs1443914	13	53917230	AL450423.1	T	C	0.475	1	0.0162	0.0024	2.80E-11
32	rs12435797	14	73797669	NUMB	G	T	0.1859	1	-0.0173	0.0031	3.70E-08
33	rs2006281	14	104000000	CTD-2134A5.4	C	T	0.4981	1	0.0135	0.0024	3.40E-08
34	rs2386584	15	91539572	PRC1	T	G	0.3835	1	-0.0166	0.0025	2.80E-11
35	rs285026	16	77100089	MON1B	G	T	0.4297	1	-0.0138	0.0025	1.90E-08
36	rs11871043	17	43172849	NMT1	T	C	0.4213	1	0.0149	0.0025	1.70E-09
37	rs11079993	17	50301552	snoZ178	G	T	0.3825	1	-0.0173	0.0025	5.70E-12
38	rs62098013	18	50863861	DCC	G	A	0.3631	1	-0.0169	0.0026	4.00E-11
39	rs2424248	20	19650324	SLC24A3	G	A	0.1255	1	0.023	0.0037	3.70E-10

Genomic risk loci are as defined by FUMA. Genomic Locus = numeric label (1–39), rsID = SNP rsID label, chr = chromosome, pos = position in base-pairs, Nearest Gene = nearest mapped gene, A1 = effect allele, A2 = non-effect allele, MAF = minor allele frequency (MAF here refers to A1 frequency as all values are < 0.5 i.e. A1 is the minor allele as well as the effect allele), r2 = imputation r-squared value, beta = association beta value, se = standard error of beta, P = P value of association (GWAS P value).

Post-GWAS analyses including gene expression and gene-level association testing was carried out using FUMA. Gene-level association tests (MAGMA gene-based test) revealed 113 genes across 39 genomic risk loci significantly associated with MCP (S1–S3 Figs), including genes with roles in neuronal adhesion and guidance, regulation of neural development and neurotransmitter receptor function.

**Fig 2:**
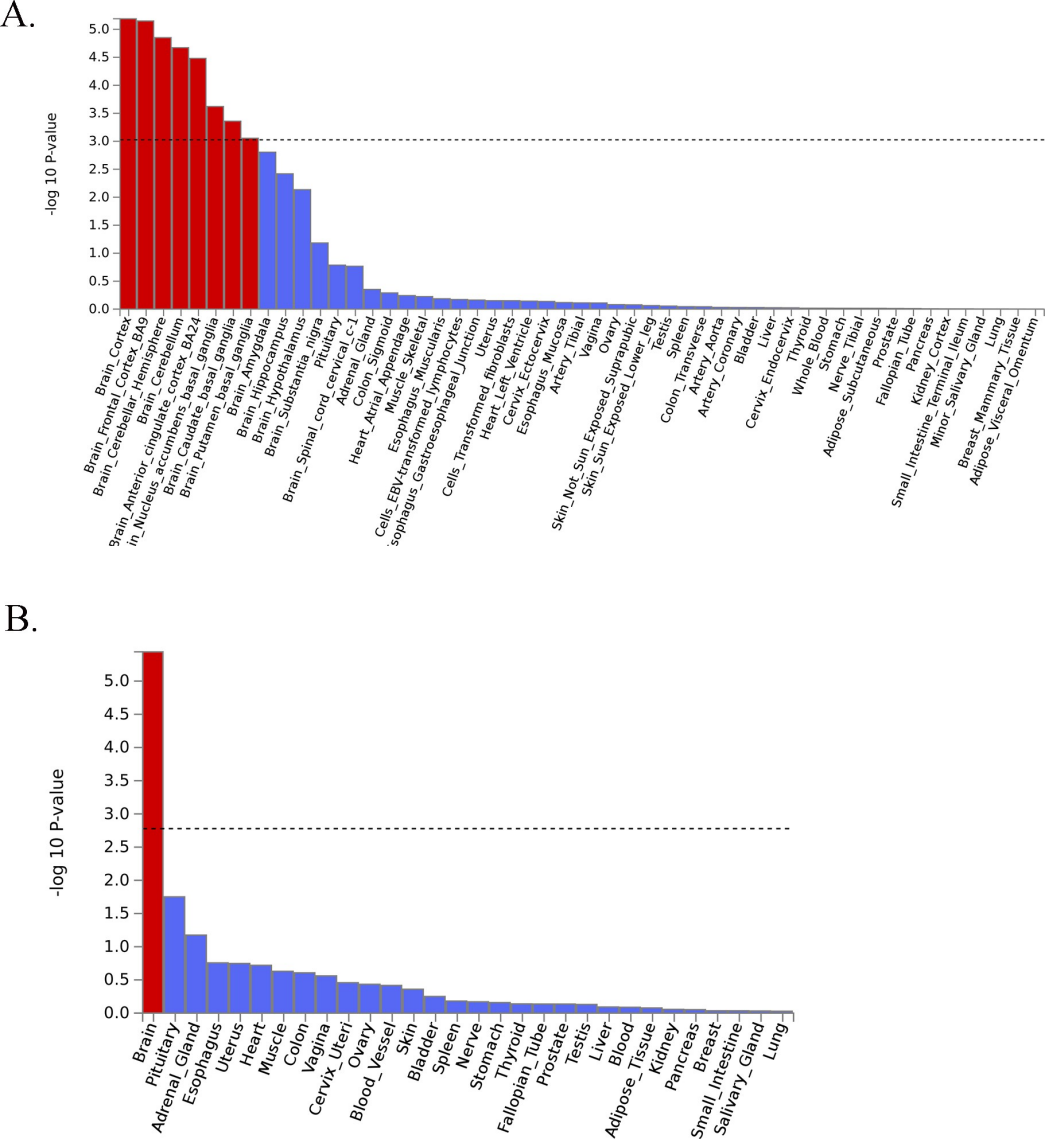
A) GteX Output–General Tissues. B) GteX Output–Detailed Tissues. Fig 2A and 2B. GTeX output–General Tissues and Detailed Tissues.

Analysis of Gene Ontology (GO) annotations revealed 3 significant categories (Table 2: Bonferroni-corrected p < 0.05). The significant categories were enriched for terms including neurogenesis and synaptic plasticity, DCC-mediated attractive signalling, neuron projection guidance and central nervous system neuron differentiation, amongst others. Genes of interest (n = 35) designated based on gene-level association tests and on annotation of genes at the identified genomic loci (see S1 Text) are listed in S2 Table. Analysis of tissue-level expression showed significant enrichment of brain-expressed genes, particularly in the cortex and cerebellum (Fig 2),

**Table 2:** GO annotations.

Gene Set	N genes	Beta	SE	SE	P	Pbon
**GO_bp:go_neuron_projection_guidance**	**195**	**0.335**	**0.0341**	**0.0711**	**1.25E-06**	**0.013361**
**Curated_gene_sets:reactome_dcc_mediated_attractive_signaling**	**13**	**1.45**	**0.0381**	**0.313**	**1.94E-06**	**0.020616**
**GO_bp:go_central_nervous_system_neuron_differentiation**	**158**	**0.362**	**0.0331**	**0.0811**	**4.05E-06**	**0.043154**

Significant GO annotations (ranked by p value) are shown. Beta = beta coefficient value from the FUMA MAGMA gene-set analyses for this Gene Ontology (GO) gene set, SE = standard error of beta, Pbon = Bonferroni-corrected p value. ‘GO_bp’ and ‘Curated_gene_sets’ refers to Gene Ontology categories biological processes and curated gene sets respectively [27].

### Genetic correlations

Genetic correlations between MCP and 22 traits were estimated via LD-score regression using ldsc [28]. The psychiatric phenotype most significantly genetically correlated with MCP was MDD (Table 3: r_g_ = 0.53, p_FDR_ = 1.69e-78) while the largest significant genetic correlation coefficient was for MCP and depressive symptoms (Table 3: r_g_ = 0.59, p_FDR_ = 6.19e-65). MCP was also positively genetically correlated with neuroticism (Table 3: r_g_ = 0.40), anxiety (Table 3: r_g_ = 0.49), schizophrenia (Table 3:r_g_ = 0.10), cross-disorder psychiatric phenotype (Table 3: r_g_ = 0.13) and PTSD (Table 3: r_g_ = 0.41). Significant negative genetic correlations were observed between MCP and subjective well-being (Table 3: r_g_ = -0.36), autism spectrum disorder (Table 3: ASD; r_g_ = -0.10) and between MCP and anorexia nervosa (Table 3: AN; r_g_ = -0.06). There was no significant genetic correlation between MCP and Bipolar disorder (Table 3: BD; P_FDR_ > 0.05). In relation to the immune-related disorders, rheumatoid arthritis (Table 3: r_g_ = 0.16) and asthma (Table 3: r_g_ = 0.22) were significantly positively genetically correlated with MCP, as was primary biliary cholangitis (Table 3: r_g_ = 0.10), while systemic lupus erythematosus (SLE), ulcerative colitis and Crohn disease were not (P_FDR_ > 0.05). BMI was significantly genetically correlated with MCP (Table 3: r_g_ = 0.31), while low relative amplitude, a circadian rhythmicity phenotype, exhibited a significant negative genetic correlation with MCP (Table 3: r_g_ = -0.30). There was no correlation between Parkinson disease and MCP (P_FDR_ > 0.05). Non-significant genetic correlation results are shown in S3 Table.

**Table 3:** Genetic correlations between MCP and multiple traits.

Trait	rg	se	z	h2	P_h2_ (fdr)	source	PMID	Category	p	P (fdr-corrected)
MDD	0.53	0.03	18.92	0.077	1.25E-47	PGC	29700475	psychiatric	7.68E-80	1.69E-78
Depressive symptoms	0.59	0.03	17.16	0.047	6.87E-29	ld_hub	27089181	psychiatric	5.63E-66	6.19E-65
BMI	0.31	0.02	15.69	0.138	5.42E-59	GIANT consortium	25673413	anthropometric	1.90E-55	1.39E-54
Neuroticism	0.4	0.03	11.9	0.089	3.66E-26	ld_hub	27089181	personality	1.24E-32	6.82E-32
Subjective well being	-0.36	0.04	-8.94	0.025	2.77E-32	ld_hub	27089181	psychiatric	3.78E-19	1.66E-18
Low Relative Amplitude	-0.3	0.05	-6.37	0.053	3.03E-13	In-house analysis	30120083	circadian	1.91E-10	7.00E-10
Rheumatoid Arthritis	0.16	0.03	4.7	0.160	7.41E-08	ld_hub	24390342	autoimmune	2.64E-06	8.30E-06
Anxiety (Case-Control)	0.49	0.11	4.53	0.081	0.00405	PGC	26754954	psychiatric	5.91E-06	1.63E-05
Schizophrenia	0.1	0.03	4.08	0.443	6.56E-79	PGC	25056061	psychiatric	4.50E-05	1.10E-04
Asthma	0.22	0.06	3.63	0.123	3.53E-06	ld_hub	17611496	autoimmune	3.00E-04	6.60E-04
PGC cross-disorder analysis	0.13	0.04	3.54	0.172	7.89E-36	ld_hub	23453885	psychiatric	4.00E-04	8.00E-04
PTSD (European Ancestry)	0.41	0.12	3.28	0.097	0.030855	PGC	28439101	psychiatric	0.001047	1.92E-03
Autism spectrum disorder	-0.1	0.04	-2.22	0.451	9.38E-17	ld_hub	NA	psychiatric	0.026	0.0443
Primary biliary cirrhosis	0.1	0.04	2.17	0.376	1.11E-08	ld_hub	26394269	autoimmune	0.03	0.047
Anorexia Nervosa	-0.06	0.03	-2.14	0.556	2.18E-63	ld_hub	24514567	psychiatric	0.032	0.0471

rg = genetic correlation coefficient value, se = standard error of correlation value, z = z value, h2 = SNP-heritability value, ph2(fdr) = p value (FDR-corrected) for SNP-heritability, source = source of GWAS summary statistics, PMID = PubMed ID of associated paper (if applicable), p = p value for genetic correlation coefficient, p(fdr) = FDR-corrected p value for genetic correlation coefficient.

### Mendelian randomisation of MCP and major depressive disorder

Mendelian Randomisation with Robust Adjusted Profile Score (MR-RAPS) analysis was performed to investigate causal relationships between MDD and MCP, first with MDD as the exposure and MCP as the outcome. QQ plots, leave-one out versus t-value plots (S4 Fig) and Anderson-Darling/ Shapiro-Wilk test p values indicated that models without dispersion were best-fitting (S4 Table rows 1–3, p_AD_ > 0.05, p_SW_ > 0.05). Effects of outliers (idiosyncratic pleiotropy) are not ameliorated in models with dispersion despite robust regression (S4D, S4E and S4F Fig right-hand panels). The model allowing the greatest amelioration of pleiotropy is one without over-dispersion and with a Tukey loss function (S4 Table: row 3, S4C Fig). This indicates idiosyncratic pleiotropy (pleiotropy in some but not all instruments), i.e. that a subset of instruments may affect MCP through pathways other than via MDD (the exposure). The causal effect of MDD on MCP is positive and significant at beta = 0.019 and p = 0.0006, but the diagnostic plots show a ‘swapping’ of sign for the causal estimate (S4 Fig), suggesting that there is not a truly significant causal effect of MDD on MCP.

MR-RAPS analyses were then carried out with MCP as the exposure and MDD as the outcome. Models with dispersion are a better fit than those without (S5A, S5B, S5C vs S5D, S5E and S5F Fig, S5 Table: rows 4–6, p_AD_ > 0.05, p_SW_ > 0.05, pτ << 0.05). This indicates that effectively all instruments are pleiotropic (affecting MDD through pathways other than via MCP). The causal effect of MCP on MDD is positive and significant at beta = 0.16 and p = 0.047.

Overall, this analysis suggests a causal effect of MCP on MDD.

### Relationship between multisite chronic pain and chronic widespread pain

Polygenic Risk Score (PRS) analyses were carried out to examine the relationship between MCP and chronic widespread pain in UK Biobank. Increasing MCP PRS value was significantly associated with having chronic pain all over the body (S6 Table: p = 1.45 x 10^−109^), with each per-standard-deviation increase in PRS associated with a 63% increase in the odds of having chronic widespread pain.

A secondary GWAS of chronic widespread pain (CWP) was carried out, the results from which were used in LD score regression analysis to determine the genetic correlation between CWP and MCP. This was found to be large (r_g_ = 0.83) and significant (p = 2.45 x 10^−54^). A lookup analysis was also carried out using the CWP GWAS summary statistics, and >90% of SNPs showed consistent direction of effect between MCP and CWP (S7 Table). In addition, a paired t-test of MCP versus CWP effect values showed that they are not significantly different overall (t = -1.82, p = 0.07).

### LocusZoom plots

LocusZoom plots for independent, genome-wide significant loci, calculated according to the supplementary methods detailed in S1 Text, are shown in S6 Fig.

## Discussion

We identified 76 independent genome-wide significant SNPs associated with MCP across 39 loci. The genes of interest had diverse functions, but many were implicated in nervous-system development, neural connectivity and neurogenesis.

### Genes of interest identified in GWAS of MCP

Potentially interesting genes included *DCC* (Deleted in Colorectal Cancer a.k.a. DCC netrin 1 receptor) which encodes DCC, the receptor for the guidance cue netrin 1, which is important for nervous-system development [29]. *SDK1* (Sidekick Cell Adhesion molecule 1) is implicated in HIV-related nephropathy in humans [30] and synaptic connectivity in vertebrates [31], and *ASTN2* (Astrotactin 2) is involved in glial-guided neuronal migration during development of cortical mammalian brain regions [32].

*MAML3* (Mastermind-Like Transcriptional coactivator 3) is a key component of the Notch signalling pathway [33,34], which regulates development and maintenance of a range of cell and tissue types in metazoans. During neurogenesis in development the inhibition of Notch signalling by Numb promotes neural differentiation [35]. Numb is encoded by *NUMB* (Endocytic Adaptor Protein), which was also associated with MCP. In the adult brain Notch signalling has been implicated in CNS plasticity across the lifespan [35].

*CTNNA2* (Catenin Alpha 2) encodes a protein involved in cell-cell adhesion [36], found to play a role in synapse morphogenesis and plasticity [37,38]. *CEP120* (Centrosomal Protein 120) encodes Cep120, vital for Interkinetic Nuclear Migration (INM) in neural progenitor cells of the cortex [39]. *KNDC1* (Kinase Non-Catalytic C-Lobe Domain Containing 1) encodes v-KIND in mice, linked to neural morphogenesis in the cortex [40], and KNDC1 in humans, linked to neuronal dendrite development and cell senescence [41]. *SOX6* (SRY-Box 6) is part of the *Sox* gene family, first characterised in mouse and human testis-determining gene *Sry* [42] and encoding transcription factors involved in a range of developmental processes [43,44]. *SOX6* may be involved in development of skeletal muscle [43], maintenance of brain neural stem cells [45] and cortical interneuron development [46], and variants in this gene have been associated with bone mineral density in both white and Chinese populations [47]. *CA10* (Carbonic Anhydrase 10) is predominantly expressed in the CNS, encoding a protein involved in development and maintenance of synapses [48]. *DYNC1I1* (Dynein Cytoplasmic 1 Intermediate Chain 1) encodes a subunit of cytoplasmic dynein, a motor protein which plays a role in cargo transport along microtubules, including in the function of neuronal cells [49]. *UTRN* (Utrophin) is a homologue of Duchenne Muscular Dystrophy gene (*DMD*), encoding utrophin protein which is localised to the neuromuscular junction (NMJ) [50]. Utrophin has also been implicated in neutrophil activation [51], dystrophin-associated-protein (DPC)-like complex formation in the brain [52], and is expressed during early foetal brain development in neurons and astrocytes [53].

*FOXP2* encodes a member of the FOX family of transcription factors, which are thought to regulate expression of hundreds of genes in both adult and foetal tissue, including the brain [54]. These transcription factors may play an important role in brain development, neurogenesis, signal transmission and synaptic plasticity [55]. *FOXP2* is essential for normal speech and language development [56]. *GABRB2* encodes a GABA (gamma-aminobutyric acid) type A receptor beta subunit. These pentameric chloride channels mediate fast inhibitory synaptic transmission and are extremely important for network function in many brain regions, with the b2 subunit forming part of the most widely expressed receptor across the mammalian brain [57,58].

Another group of genes associated with MCP were linked to cell-cycle progression, DNA replication and apoptosis such as *EXD3* (Exonuclease 3’-5’ Domain Containing 3), which encodes a protein involved in maintaining DNA fidelity during replication (‘proof-reading’) [59]. *BBX* (HMG-Box Containing protein 2) encodes an HMG (high mobility group) box-containing protein necessary for cell-cycle progression from G1 to S phase [60]. *STAG1* (Cohesin Subunit SA-1) encodes a cohesin-complex component–cohesin ensures sister chromatids are organised together until prometaphase [61–63]. *ANAPC4* (Anaphase Promoting Complex Subunit 4) encodes a protein making up the anaphase promoting complex (APC), an essential ubiquitin ligase for eukaryotic cell-cycle progression [64]. *PRC1* (Protein Regulator of Cytokinesis 1) is involved in the regulation of cytokinesis [65], the final stage of the cell cycle. *Y RNA* (Small Non-Coding RNA, Ro-Associated Y3) encodes a small non-coding Y RNA. These RNAs have been implicated in a wide range of processes, including cell stress response, DNA replication initiation and RNA stability [66]. *FAM120A* (Oxidative Stress-Associated Src Activator) encodes an RNA-binding protein which regulated Src-kinase activity during oxidative stress-induced apoptosis [67]. The protein encoded by *MON1B* (MON1 Homolog B, Secretory Trafficking Associated) is necessary for clearance of cell ‘corpses’ following apoptosis, with defects associated with autoimmune pathology [68]. *FAF1* (Fas Associated Factor 1) encodes a protein which binds the Fas antigen to initiate or facilitate apoptosis, amongst a wide range of other biological processes (including neuronal cell survival) [69].

Several MCP associated genes have been previously implicated in diseases such as Brugada Syndrome 9 and Spinal ataxia 19 & 22 (*KCND3*) [70–72], Systemic lupus erythematosus (SLE) (Y RNAs) [66], Joubert syndrome 31 and short-rib thoracic dysplasia 13 (*CEP120*) [73], Amyotrophic lateral sclerosis (ALS) (*FAF1*) [74], Urbach-Wiethe disease (*ECM1*) [75,76], mental retardation and other cohesinopathies such as Cornelia de Lange Syndrome (*STAG1*) [77,78], split hand/ split foot malformation (*DYNC1I1*) [79,80], and a wide range of cancers (*PRC1*) [81]. Other disorders found to involve MCP-related genes include schizophrenia (*FOXP2* and *GABRB2*) [82–88], intellectual disability and epilepsy (*GABRB2*) [89], and neuroleptic-induced tardive dyskinesia (*GABRB2*) [90].

Several GWASs of chronic pain at specific body sites, of specific pain types such as neuropathic pain, and of diseases and disorders where chronic pain is a defining symptom, have been carried out previously (reviewed by [10], [91]). *DCC* and *SOX5* (which jointly functions with *SOX6* in chondrogenesis) have been associated with chronic back pain [92], *GABRB3* (encoding one of three beta subunits of the GABA A receptor along with *GABRB2*) has been associated with migraine and fibromyalgia [10], and *ASTN2* and *SLC24A3* have been associated with migraine [10,93]

Overall, this indicated that MCP, a chronic pain phenotype, involves structural and functional changes to the brain, including impact upon neurogenesis and synaptic plasticity both during development and in adulthood. Also implicated was regulation of cell-cycle progression and apoptosis. This is also supported by GO categories DCC-mediated attractive signalling, neuron projection guidance and CNS neuron differentiation being significantly associated with MCP. There was also evidence of pleiotropy, with genes associated with a range of neurodegenerative, psychiatric, developmental and autoimmune disease traits, as well as being associated with MCP.

### Genetic correlations

Chronic pain and chronic pain disorders are often comorbid with psychiatric and neurodevelopmental disorders [11]. This has been observed for Major Depressive Disorder (MDD) [8,94], post-traumatic stress-disorder (PTSD) [95–99], schizophrenia [100–102] and bipolar disorder (BD) [94,103]. There are also reported differences in the perception of pain and interoception (sensing and integration of bodily signals) for people with schizophrenia [104,105], anorexia nervosa (AN) [106–108] and autism spectrum disorders (ASD) [109,110], with some evidence of an increase in pain thresholds for AN and ASD.

There is significant cross-talk between the immune system and nervous system in nociception and sensitisation leading to chronic pain [12,13], and many autoimmune disorders cause or have been associated with chronic pain including neuroinflammation implicated in development of neuropathic pain [111].

Similarly, obesity and chronic pain are often comorbid, with extrinstic factors such as MDD and sleep disturbance also impacting on chronic pain [14,15]. Obesity and related chronic inflammation may affect chronic pain [112], and adipose tissue is metabolically active in ways that can affect pain perception and inflammation [113–115].

Sleep changes and loss of circadian rhythm is common in those with chronic pain [16], and myriad chronic diseases, including chronic pain, have shown diurnal patterns in symptom severity, intensity and mortality [116,117]. Chronic pain is also a common component of many neurological diseases, particularly Parkinson’s disease [17], and disorders such as Multiple Sclerosis and migraines are considered neurological in nature.

MCP showed moderate positive genetic correlation with a range of psychiatric disorders including MDD, SCZ, and PTSD, along with traits anxiety and neuroticism. The magnitude of genetic correlation between MCP and MDD was similar to that shown for von Korff chronic pain grade (a chronic pain phenotype) and MDD by McIntosh et al via a mixed-modelling approach (ρ = 0.53) [8]. This is in line with previous observations of association and indicates that shared genetic risk factors exist between MCP and a range of psychiatric disorders, most notably MDD, and that the genetic correlation between MCP and MDD matches with that between MDD and von Korff CPG, a validated chronic-pain questionnaire-derived phenotype [7].

Autoimmune disorders rheumatoid arthritis, asthma and primary biliary cholangitis showed positive genetic correlation with MCP. However, gastrointestinal autoimmune disorders UC, IBD and Crohn’s Disease did not. This suggests separate genetic variation and mechanisms underlying chronic pain associated with these autoimmune disorders compared to those outwith the digestive system. Pain related to inflammatory bowel diseases may represent something less ‘chronic’ and more ‘on-going acute’, as stricture, abscesses and partial or complete obstruction of the small bowel result in pain [118]. Structural and functional brain changes associated with the transition to chronic pain may also play a less central role in gastrointestinal autoimmune disorder-associated pain, due to potential for the enteric nervous system (ENS) to act independently from the CNS, and the role of the gut-brain axis (GBA) [119,120].

There was significant negative genetic correlation between low relative amplitude, a circadian rhythmicity phenotype indicating poor rhythmicity [121]. Opposing direction of effect of genetic variants on MCP versus low RA may mean that insomnia and other sleep difficulties (for which low RA represents a proxy phenotype) associated with MCP are due to environmental and lifestyle factors related to chronic pain, rather than shared genetic factors predisposing to increased risk for both traits. There was also significant negative genetic correlation between MCP and both AN and ASD, which may be linked to changes in interoception and atypical pain experience seen in individuals with these conditions [106–110], and may suggest a genetic basis for increased pain thresholds.

### SNP heritability of MCP

LDSR analyses gave a heritability estimate of 10.2% for MCP, lower than the pseudo-h^2^ estimate of 10.3% given by BOLT-LMM. this suggests SNP-heritability (h^2^) of MCP to be roughly-10%, slightly lower than an estimate of ‘any chronic pain’ of 16%, and markedly lower than a heritability estimate of 30% for ‘severe chronic pain’ derived from a pedigree-based analyses [3].

### Causal associations between MDD and MCP

Mendelian randomisation analyses indicated a causal effect of MCP on MDD, with widespread pleiotropy and a less significant causal estimate value for MCP as the exposure–this suggests most instruments for MCP are pleiotropic, affecting MDD through pathways other than directly through MCP. In contrast, only a small subset of instruments for MDD as the exposure were found to be pleiotropic.

### Relationship between MCP and CWP

It has been argued that CWP and other clinical syndromes involving chronic pain all over the body represent the upper end of a spectrum of centralisation of pain, or the extreme of a chronic pain state [122]. It has also been argued that there are not “natural cut-off points” when it comes to chronic widespread pain versus localised chronic pain [25]. In support of this view, the MCP PRS was significantly associated with increased odds of having chronic pain all over the body/ CWP, suggesting that chronic widespread pain may in fact represent the upper end of a spectrum of ‘widespreadness’ of chronic pain, as previously suggested [25,122], and that there are likely to be genetic variants that predispose both to MCP and to CWP.

### Conclusions & limitations

Multisite chronic pain (MCP), a chronic pain phenotype defined as the number of sites at which chronic pain is experienced, is a complex trait with moderate heritability. To date, this study represents the largest GWAS of any chronic pain phenotype and elucidates potential underlying mechanisms of chronic pain development. Substantial genetic correlations with a range of psychiatric, personality, autoimmune, anthropometric and circadian traits were identified.

The genes potentially associated with MCP implicated neurogenesis, neuronal development and neural connectivity, along with cell-cycle and apoptotic processes, and expression was primarily within brain tissues. This is in line with theories of functional and structural changes to the brain contributing to development of chronic pain [21,24,123–125], and may also explain the genetic correlations observed. A causal effect of MCP on MDD was identified.

Although the phenotype was based on self-report, this study was very large in size and so likely had sufficient power to detect genetic variation associated with MCP. Replication of SNP associations was not possible due to the nature of chronic pain phenotyping and available cohort sizes, but several genes significantly associated with MCP have been previously associated with chronic pain conditions including chronic back pain, migraine and fibromyalgia, and genetic risk for MCP was found to be significantly associated with chronic widespread pain.

## Methods

We carried out a GWAS of Multisite Chronic Pain (MCP), a derived chronic pain phenotype, in 387,649 UK Biobank participants (Table 4). UK Biobank is a general-population cohort of roughly 0.5 million participants aged 40–79 recruited across the UK between 2006 and 2010. Details on phenotyping, follow-up and genotyping have been described in detail elsewhere [126].

**Table 4:** Demographics of those included in BOLT-LMM GWAS of MCP.

chronic pain sites	male (N)	female (N)	male (%)	female (%)	age (mean)	total (N)	total (%)
0	105474	113148	48.2	51.8	56.71	218622	56.40
1	42734	49984	46.1	53.9	57.03	92718	23.92
2	18612	26000	41.7	58.3	57.29	44612	11.51
3	7771	12376	38.6	61.4	57.65	20147	5.20
4	2970	5319	35.8	64.2	57.48	8289	2.14
5	780	1723	31.2	68.8	56.53	2503	0.65
6	181	471	27.8	72.2	56.20	652	0.17
7	34	72	32.1	67.9	56.17	106	0.03
total	178556	209093	NA	NA	56.91	387649	NA

### Phenotype definition and GWAS

During the baseline investigations, UK Biobank participants were asked via a touchscreen questionnaire about “pain types experienced in the last month” (field ID 6159), with possible answers: ‘None of the above’; ‘Prefer not to answer’; pain at seven different body sites (head, face, neck/shoulder, back, stomach/abdomen, hip, knee); or ‘all over the body’. The seven individual body-site pain options were not mutually exclusive and participants could choose as many as they felt appropriate. Where patients reported recent pain at one or more body sites, or all over the body, they were additionally asked (category ID 100048) whether this pain had lasted for 3 months or longer. Those who chose ‘all over the body’ could not also select from the seven individual body sites.

Multisite Chronic Pain (MCP) was defined as the sum of body sites at which chronic pain (at least 3 months duration) was recorded: 0 to 7 sites. Those who answered that they had chronic pain ‘all over the body’ were excluded from the GWAS as there is some evidence that this phenotype relating to widespread pain can be substantially different from more localised chronic pain [94] and should not, therefore, be considered a logical extension of the multisite scale. 10,000 randomly-selected individuals reporting no chronic pain were excluded from the GWAS to use as controls in subsequent polygenic risk score (PRS) analyses.

SNPs with an imputation quality score of less than 0.3, Minor Allele Frequency (MAF) < 0.01 and Hardy-Weinberg equilibrium (HWE) test p < 10^−6^ were removed from the analyses. Participants whose self-reported sex did not match their genetically-determined sex, those who had putative sex-chromosome aneuploidy, those considered outliers due to missing heterozygosity, those with more than 10% missing genetic data and those who were not of self-reported white British ancestry were excluded from analyses.

An autosomal GWAS was run using BOLT-LMM [127], with the outcome variable, MCP, modelled as a linear quantitative trait under an infinitesimal model, and the model adjusted for age, sex and chip (genotyping array). Related individuals are included and accounted for, as are any population stratification effects, via use of a genetic relatedness matrix as part of the BOLT-LMM analysis [127]. The SNP-level summary statistics from the GWAS output were analysed using FUMA [128], which implements a number of the functions from MAGMA (gene-based association testing, gene-set analyses) [129]. Tissue expression (GTEx) analysis [130] and Gene Ontology [27] and ANNOVAR [131] annotation analysis with default settings was used to characterise lead SNPs further. LocusZoom [132] was used to plot association results at higher resolution (N = 47) (S1 Text). Genomic risk loci were identified using the definition deployed by FUMA [128].

### Genetic correlation analysis

Genetic correlations between MCP and 22 complex traits selected on the basis of prior phenotypic association evidence were calculated using linkage disequilibrium score regression (LDSR) analyses [28], implemented either using the ‘ldsc’ package [28] and downloaded publicly-available summary statistics and summary statistics from in-house analyses or using LD Hub [133]. LD Hub datasets from the categories Psychiatric, Personality, Autoimmune and Neurological were selected and datasets with the attached warning note ‘Caution: using this data may yield less robust results due to minor departure from LD structure’ were excluded from the analyses. Where multiple GWAS datasets were available for the same trait, the one with the largest sample size and/or European ancestry was retained with priority given to European ancestry.

### Mendelian randomisation analysis of MCP and major depressive disorder

Mendelian randomisation analysis was carried out with MR-RAPS (MR-Robust Adjusted Profile Score; [134] using the R package ‘mr-raps’. This method is appropriate when doing MR analysis of phenotypes that are moderately genetically correlated and likely to share some pleiotropic risk loci. MDD was chosen for MR analysis as this disorder represents an important and common comorbidity with chronic pain [2,8,135]. Summary statistics from the most recent MDD GWAS meta-analysis [136], with UK Biobank and 23andMe results removed, were harmonised with MCP GWAS summary statistics following guidelines [137] as closely as possible with the available data. Bi-allelic SNPs shared between the two datasets were identified and harmonised (by ‘flipping’) with respect to the strand used to designate alleles. Reciprocal MR analysis was carried out using subsets of SNPs associated with each of the exposure traits (MCP and MDD) at p < 10^−^5. This threshold is an order of magnitude lower than suggested as part of the MR-RAPS method [134] and was chosen in order to attempt to account for ‘winner’s curse’, as independently selecting and then testing association for instruments in separate GWAS datasets was not possible in this study. The harmonisation process also involved ensuring that the effect allele was trait-increasing in the exposure trait, and that the effect allele matched between the exposure and the outcome. These selected subsets of variants were then LD-pruned at a threshold of r^2^ < 0.01 using command-line PLINK using ‘indep-pairwise’ with a 50-SNP window and sliding window of 5 SNPs [138]. This resulted in a set of 200 instruments for MCP as the exposure, and a set of 99 instruments for MDD as the exposure.

### PRS prediction of chronic widespread pain

Those who reported chronic pain all over the body were excluded from the MCP GWAS analyses above. This is because chronic pain all over the body, taken as a proxy for chronic widespread pain (CWP), may be a different clinical syndrome from more localised chronic pain, and does not necessarily directly reflect chronic pain at 7 bodily sites. To investigate the relationship between CWP and MCP, a polygenic risk score (PRS) approach was taken.

A PRS was constructed for MCP in individuals who reported chronic pain all over the body (n = 6,815; these individuals had all been excluded from the MCP GWAS), and in controls (n = 10,000 individuals reporting no chronic pain at any site, also excluded from the MCP GWAS). The PRS was calculated using SNPs associated with MCP at p < 0.01, weighting by MCP GWAS effect size (GWAS β) for each SNP. A standardised PRS (based on Z-scores) was used in all analyses, constructed by dividing the calculated PRS by its standard deviation across all samples. The ability of the standardised PRS to predict chronic widespread pain status was investigated in logistic regression models adjusted for age, sex, genotyping array and the first 8 genetic principal components.

Individual-level data are available via application to UK Biobank. Multisite chronic pain GWAS summary statistics are available via contacting the authors and will be submitted to UK Biobank for publication at their website.

## Supporting information

S1 Text Supplementary information. Supplementary methods and background information on defining genes of interest, MR-RAPS and LocusZoom.
(DOCX)

S1 Fig Summary of findings at the identified loci. Further information on genomic risk loci as identified by FUMA is shown, including locus size in terms of base-pairs (Size(kb)), number of SNP associations within the locus range (#SNPs), number of genes mapped to the locus (#mapped genes) and the number of genes physically located within the locus (#genes physically located in loci).
(TIF)

S2 Fig Gene-Based test (MAGMA) manhattan plot. Results of the MAGMA gene-based test results implemented via FUMA are shown, with the SNPs with the top 10 most-significant gene associations (by Bonferroni-corrected gene-based test p value) labelled. Significance (a Bonferroni-corrected p-value of less than ~6 on the -log10 scale) is indicated by the dashed red line.
(TIF)

S3 Fig Gene-based test QQ plot. Observed versus expected gene-based test p values on the -log10 scale are shown.
(TIF)

S4 Fig MR-RAPS MDD Exposure QQ Plots. Quantile-Quantile plots (left-hand panels), and leave-one-out beta estimate versus t-value plots (right-hand panels) for each of the six models fitted during MR-RAPS analysis with MDD as the exposure are shown (A-F).
(PDF)

S5 Fig MR-RAPS MCP Exposure QQ Plots. Quantile-Quantile plots (left-hand panels), and leave-one-out beta estimate versus t-value plots (right-hand panels) for each of the six models fitted during MR-RAPS analysis with MCP as the exposure are shown (A-F).
(PDF)

S6 Fig LocusZoom Plots. Plots of the 46 SNP regions +/- 1 mega-base pairs flanking the region are shown. Mb = mega-base pairs, cM = centimorgans, -log10(p-value) refers to GWAS p value on -log10 scale. Lower panel shows genes in the plotted region. Lead SNP is marked with a purple diamond point and labelled with rsID.
(PDF)

S1 Table MR-RAPS Models. Six different regression models fitted during MR-RAPS analysis and their corresponding S1 or S2 Figs label (A-F) are shown. L2 = L2 loss function, huber = Huber loss function, tukey = Tukey loss function.
(PDF)

S2 Table Genes of Interest. Genes of interest as determined via Supplementary Methods. Note that this is distinct from MAGMA gene-based test results (N significant genes there = 113).
(DOCX)

S3 Table Non-significant Genetic Correlation Results. (DOCX)

S4 Table MR RAPS Results MDD Exposure (all models). MR results for MDD-exposure. Β refers to the causal effect, SE (β) and P (β) to the standard error and p value of β, P (AD) to the Anderson-Darling test of normality p value, P (SW) to the Shapiro-Wilk test of normality p value, tau to the over-dispersion statistic size and P (τ) to the p value. C.F = corresponding QQ plot panel for the model. P (τ) was calculated from the tau estimate and its standard error [139]. The row of the table corresponding to the regression model found to be best-fitting is in bold.
(DOCX)

S5 Table MR RAPS Results MCP Exposure (all models). MR results for chronic pain-exposure. Β refers to the causal effect, SE (β) and P (β) to the standard error and p value of β, P (AD) to the Anderson-Darling test of normality p value, P (SW) to the Shapiro-Wilk test of normality p value, τ to the over-dispersion statistic size and P (τ) to the p value. P (τ) was calculated from the τ estimate and its standard error [139]The row of the table corresponding to the regression model found to be of best fit is in bold.
(DOCX)

S6 Table PRS Results. Regression beta coefficient values (Estimate), odds ratios (OR), and P values. The reference level for ‘sex’ is set to female, PRS = z-polygenic risk score.
(DOCX)

S7 Table Association of top MCP-SNPs with CWP in UK Biobank. GL = Genomic Locus, Chr = chromosome, pos = position, base pairs, mcp_A2 = other allele (MCP GWAS), mcp_A1 = effect allele (MCP GWAS), mcp_beta = effect (beta) (MCP GWAS), mcp_se = standard error of beta (MCP GWAS), cwp_A1 = effect allele (CWP GWAS), cwp_A2 = other allele (CWP GWAS), cwp_beta = effect (beta) (CWP GWAS), cwp_se = standard error of the beta (CWP GWAS), cwp_gwas_p = gwas P value (CWP GWAS).
(DOCX)
