# Supplementary material for: Genome-wide association study of multisite chronic pain in UK Biobank
Source: PLoS Genet. 2019 Jun 13;15(6):e1008164. doi: 10.1371/journal.pgen.1008164 (PMC6592570; doi:10.1371/journal.pgen.1008164)
Supplement: S1 Text — Supplementary methods and background information on defining genes of interest, MR-RAPS and LocusZoom. (DOCX) [file pgen.1008164.s001.docx]

**Supplementary Information**

**Defining Genes of Interest**

The 10 most-significant genes identified using the FUMA/MAGMA gene-based test results (ranked by Bonferroni-corrected p value) were selected for further analysis.

In addition, 46 genes identified by FUMA SNP2GENE analysis of associated loci and their annotations in ANNOVAR (output files GenomicRiskLoci.txt and annov.txt) were also selected and matched by ‘uniqID’ to make a subset of data consisting of ANNOVAR annotations for lead SNPs at 39 genomic risk loci (1 locus has 2 independent lead SNPs). Lead SNPs can have multiple ANNOVAR gene annotations. From this total, genes that were already in the MAGMA gene-based test list were removed (-7), as were RNAs and pseudogenes that were not well-characterised or associated with diseases or traits from preliminary OMIM (Online Mendelian Inheritance in Man), GeneCards and PubMed ‘Gene’ database searches.

These two lists were combined with the top 10 FUMA MAGMA gene-based test results to give N = 35 genes of interest.

**MR-RAPS**

Briefly, the main problem associated with pleiotropy in MR analyses is that instruments may be invalid due to pleiotropy, associated measurement error, weak-instrument bias, and selection bias. MR-RAPS treats pleiotropy-related issues as an ‘errors in regression’ problem (Zhao *et al.*, 2018), in contrast to MR modifications with their roots in meta-analyses (such as Egger and Inverse-Variance-Weighted (IVW) MR) which treat pleiotropy in a manner similar to heterogeneity between individual studies in a meta-analysis (Smith and Hemani, 2014; Zheng, Baird, *et al.*, 2017).

The basic model types fitted by MR-RAPS are: no pleiotropy, systematic pleiotropy (all instruments subject to pleiotropy), and a combination of idiosyncratic (only some instruments subject to pleiotropy) and systematic pleiotropy. MR-RAPS was used to fit a total of 6 regressions per analysis (Supplementary Table 1), and diagnostics for the fit to each model were evaluated to select the optimal model type prior to interpretation of the causal effect estimate.

**LocusZoom Plots**

Plots were created using Locuszoom v1.4 standalone (Pruim *et al.*, 2010) with pop flag set to EUR, build set to hg19 and source set to 1000G_Nov2014. Associated regions were defined as all SNPs in LD with the lead SNP at each locus (r^2^ > 0.1) within 500Mbp on either side of the lead SNP. Plot boundaries were set at +/- 1 Mbp flanking the associated region. See Supplementary Figures ‘LocusZoom Plots’.
