# Supplementary figures and images for: Genome-wide association study of multisite chronic pain in UK Biobank
Source: PLoS Genet. 2019 Jun 13;15(6):e1008164. doi: 10.1371/journal.pgen.1008164 (PMC6592570; doi:10.1371/journal.pgen.1008164)

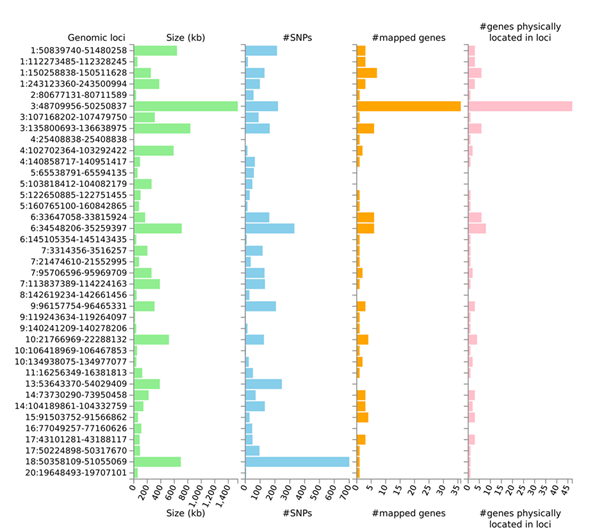

Supplement: S1 Fig — Further information on genomic risk loci as identified by FUMA is shown, including locus size in terms of base-pairs (Size(kb)), number of SNP associations within the locus range (#SNPs), number of genes mapped to the locus (#mapped genes) and the number of genes physically located within the locus (#genes physically located in loci). (TIF) [file pgen.1008164.s002.tif]

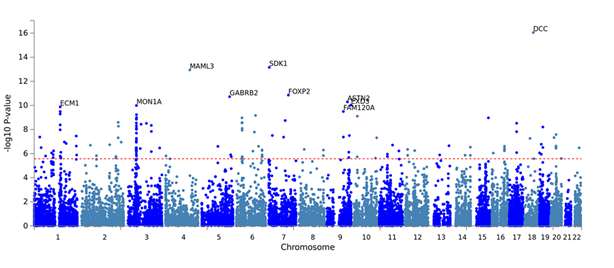

Supplement: S2 Fig — Results of the MAGMA gene-based test results implemented via FUMA are shown, with the SNPs with the top 10 most-significant gene associations (by Bonferroni-corrected gene-based test p value) labelled. Significance (a Bonferroni-corrected p-value of less than ~6 on the -log10 scale) is indicated by the dashed red line. (TIF) [file pgen.1008164.s003.tif]

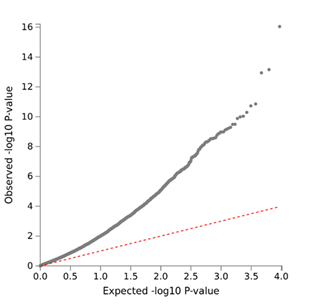

Supplement: S3 Fig — Observed versus expected gene-based test p values on the -log10 scale are shown. (TIF) [file pgen.1008164.s004.tif]

**A** Normal Q-Q Plot

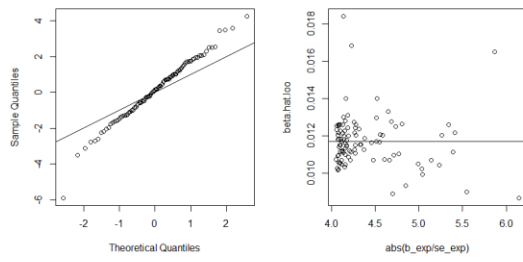

**D** Normal Q-Q Plot

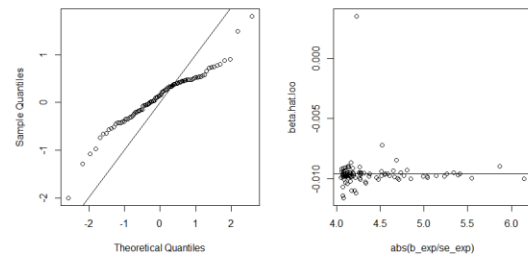

**B** Normal Q-Q Plot

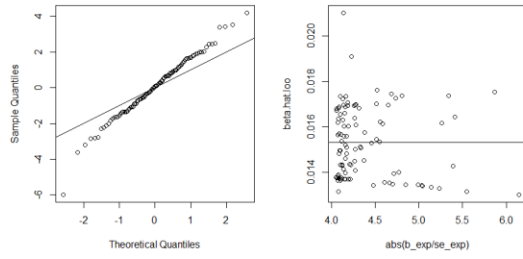

**E** Normal Q-Q Plot

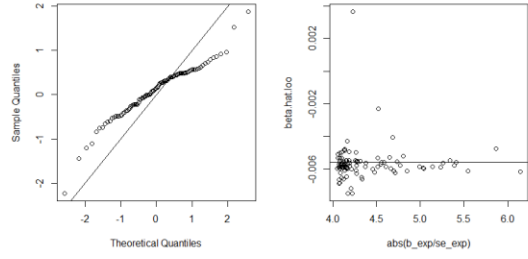

**C** Normal Q-Q Plot

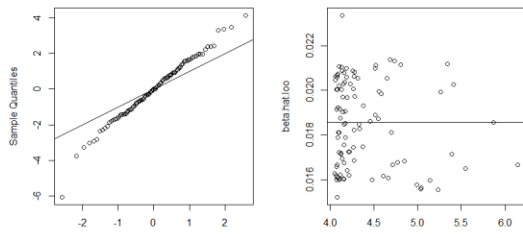

**F** Normal Q-Q Plot

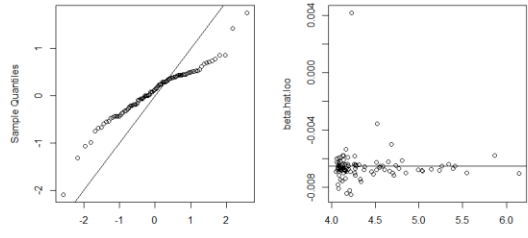

Supplement: S4 Fig — Quantile-Quantile plots (left-hand panels), and leave-one-out beta estimate versus t-value plots (right-hand panels) for each of the six models fitted during MR-RAPS analysis with MDD as the exposure are shown (A-F). (PDF) [file pgen.1008164.s005.pdf]

**A** Normal Q-Q Plot

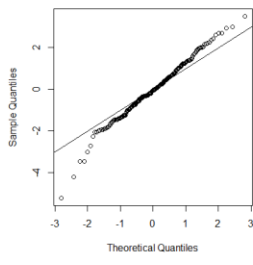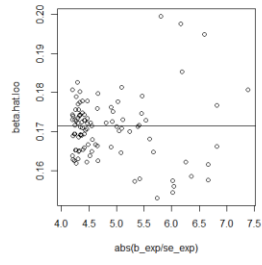

**D** Normal Q-Q Plot

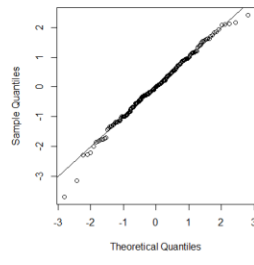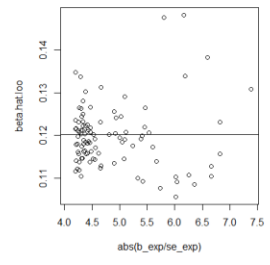

**B** Normal Q-Q Plot

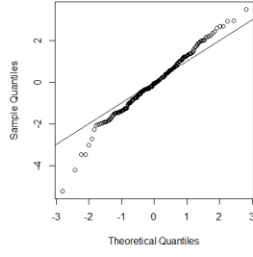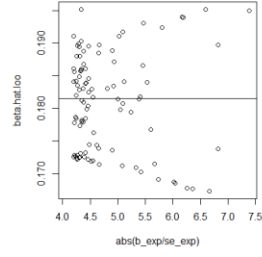

**E** Normal Q-Q Plot

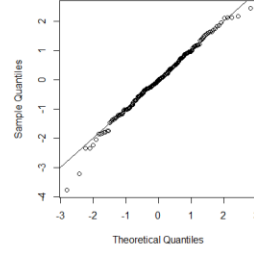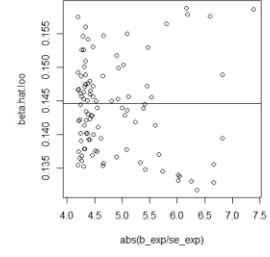

**C** Normal Q-Q Plot

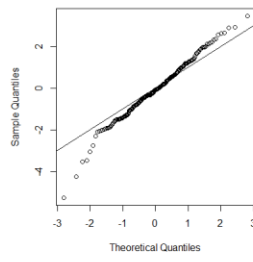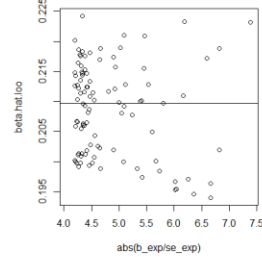

**F** Normal Q-Q Plot

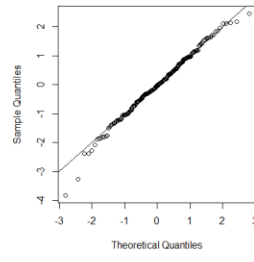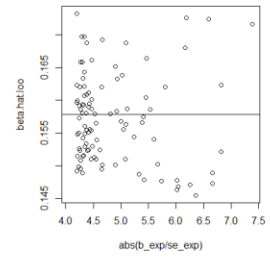

Supplement: S5 Fig — Quantile-Quantile plots (left-hand panels), and leave-one-out beta estimate versus t-value plots (right-hand panels) for each of the six models fitted during MR-RAPS analysis with MCP as the exposure are shown (A-F). (PDF) [file pgen.1008164.s006.pdf]
