# Supplementary material for: Genome-wide association study of multisite chronic pain in UK Biobank
Source: PLoS Genet. 2019 Jun 13;15(6):e1008164. doi: 10.1371/journal.pgen.1008164 (PMC6592570; doi:10.1371/journal.pgen.1008164)
Supplement: S6 Fig — Plots of the 46 SNP regions +/- 1 mega-base pairs flanking the region are shown. Mb = mega-base pairs, cM = centimorgans, -log10(p-value) refers to GWAS p value on -log10 scale. Lower panel shows genes in the plotted region. Lead SNP is marked with a purple diamond point and labelled with rsID. (PDF) [file pgen.1008164.s007.pdf]

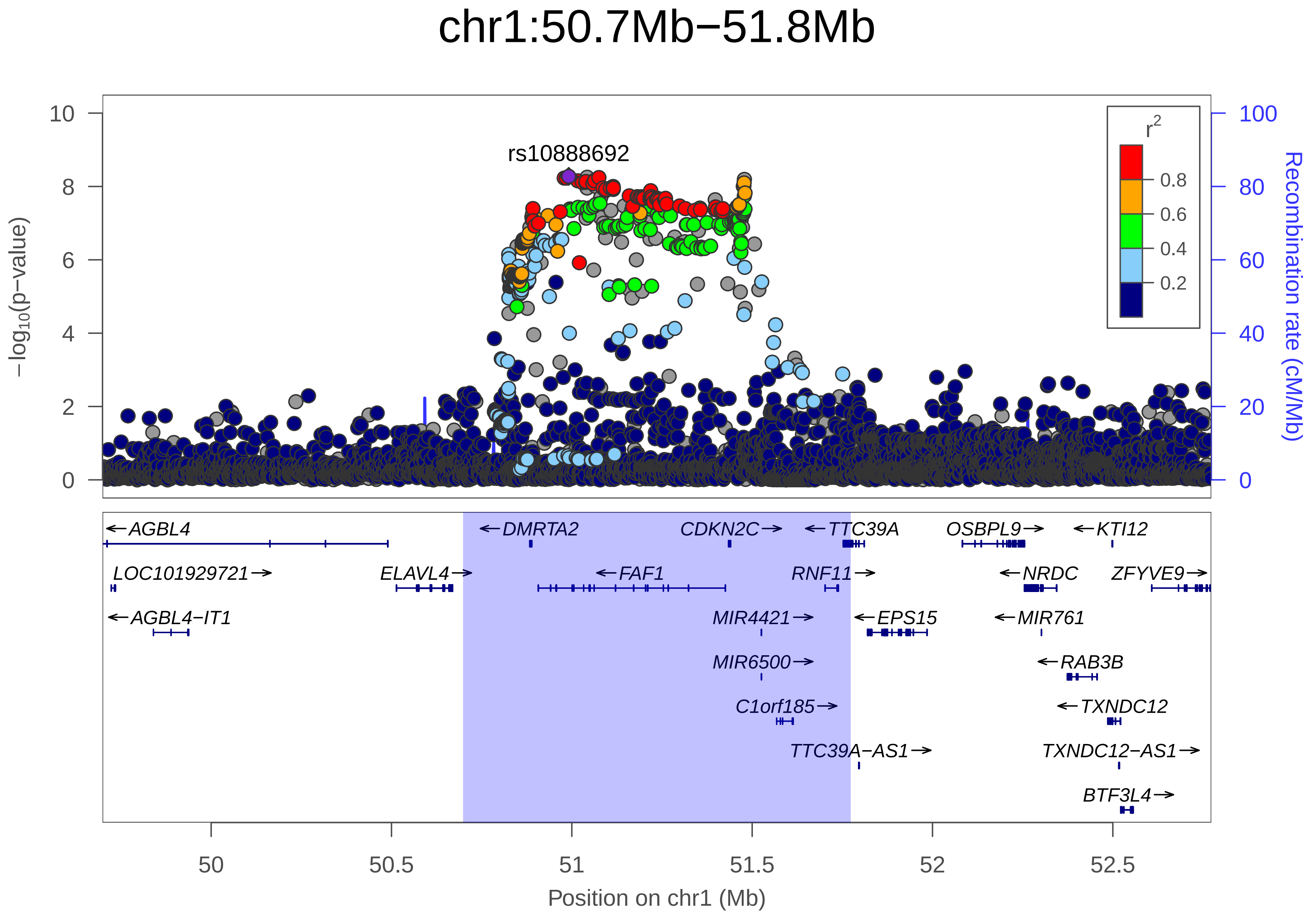
chr1:50.7Mb−51.8Mb
rs10888692
−log10(p−value)
Recombination rate (cM/Mb)
r2
0.8
0.6
0.4
0.2
0
2
4
6
8
10
0
20
40
60
80
100
←AGBL4
LOC101929721→
←AGBL4−IT1
ELAVL4→
←DMRTA2
←FAF1
CDKN2C→
MIR4421→
MIR6500→
C1orf185→
←TTC39A
RNF11→
TTC39A−AS1→
←EPS15
OSBPL9→
←NRDC
←MIR761
←RAB3B
←TXNDC12
TXNDC12−AS1→
BTF3L4→
←KTI12
ZFYVE9→
50
50.5
51
51.5
52
52.5
Position on chr1 (Mb)

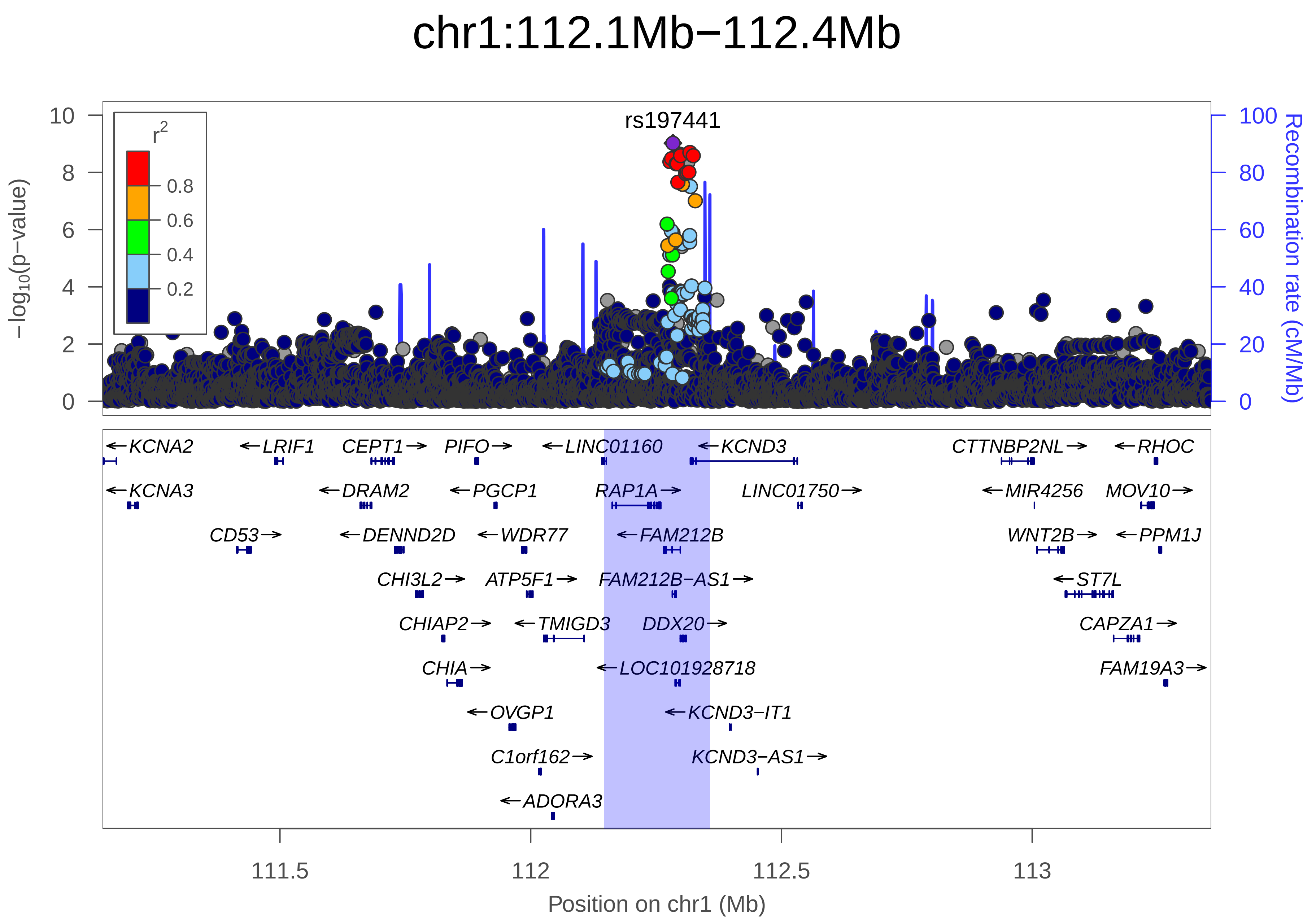

chr1:112.1Mb−112.4Mb
rs197441
r2
0.8
0.6
0.4
0.2
−log10(p−value)
Recombination rate (cM/Mb)
KCNA2
LRIF1
CEPT1
PIFO
LINC01160
KCND3
CTTNBP2NL
RHOC
KCNA3
DRAM2
PGCP1
RAP1A
LINC01750
MIR4256
MOV10
CD53
DENND2D
WDR77
FAM212B
WNT2B
PPM1J
CHI3L2
ATP5F1
FAM212B−AS1
ST7L
CHIAP2
TMIGD3
DDX20
CAPZA1
CHIA
LOC101928718
FAM19A3
OVGP1
KCND3−IT1
C1orf162
KCND3−AS1
ADORA3
111.5
112
112.5
113
Position on chr1 (Mb)

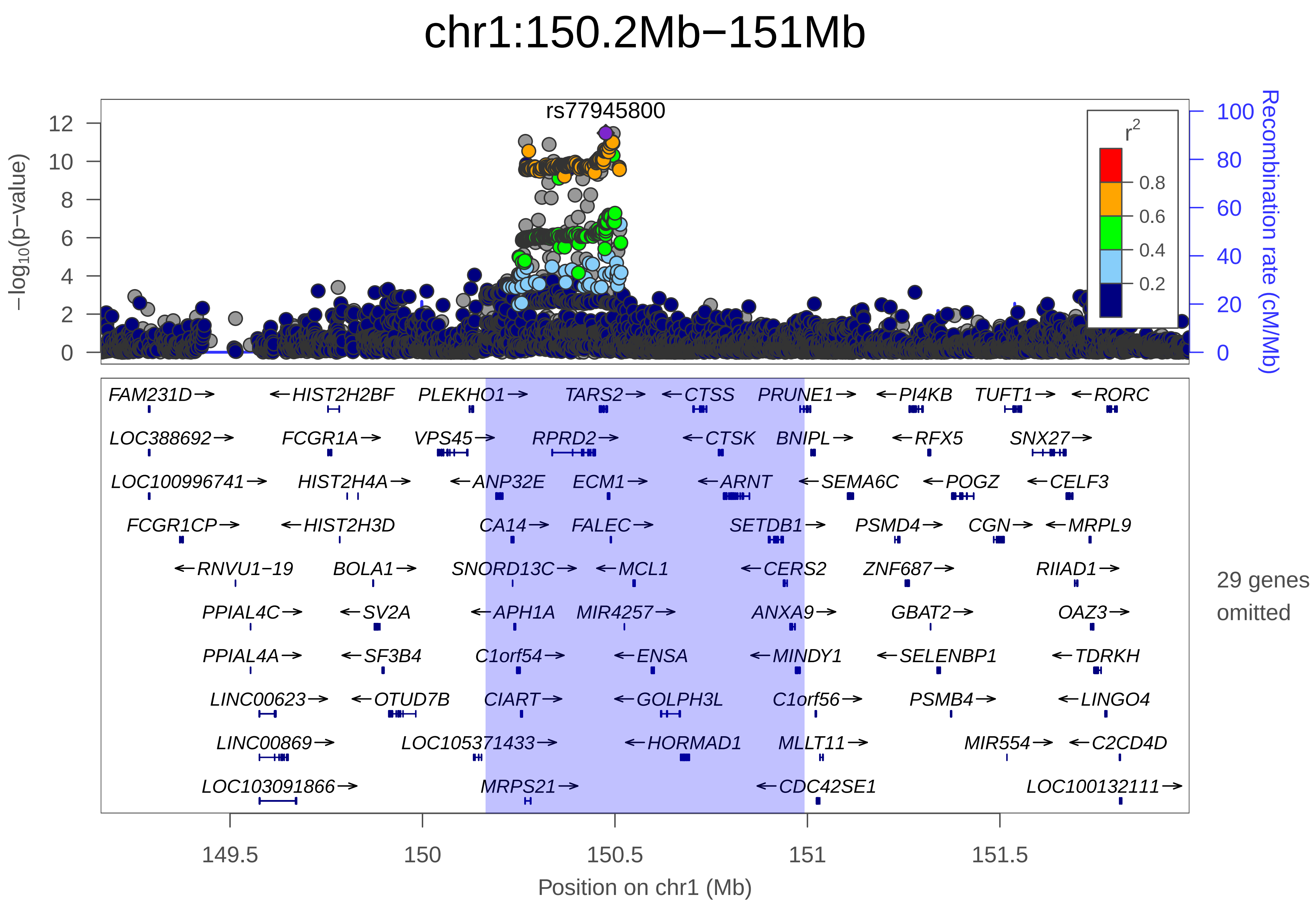
chr1:150.2Mb−151Mb
rs77945800
−log10(p−value)
Recombination rate (cM/Mb)
r2
0.8
0.6
0.4
0.2
FAM231D→ ←HIST2H2BF PLEKHO1→ TARS2→ ←CTSS PRUNE1→ ←PI4KB TUFT1→ ←RORC
LOC388692→ FCGR1A→ VPS45→ RPRD2→ ←CTSK BNIPL→ ←RFX5 SNX27→
LOC100996741→ HIST2H4A→ ←ANP32E ECM1→ ←ARNT ←SEMA6C ←POGZ ←CELF3
FCGR1CP→ ←HIST2H3D CA14→ FALEC→ SETDB1→ PSMD4→ CGN→ ←MRPL9
←RNVU1−19 BOLA1→ SNORD13C→ ←MCL1 ←CERS2 ZNF687→ RIIAD1→
PPIAL4C→ ←SV2A ←APH1A MIR4257→ ANXA9→ GBAT2→ OAZ3→
PPIAL4A→ ←SF3B4 C1orf54→ ←ENSA ←MINDY1 ←SELENBP1 ←TDRKH
LINC00623→ ←OTUD7B CIART→ ←GOLPH3L C1orf56→ PSMB4→ ←LINGO4
LINC00869→ LOC105371433→ ←HORMAD1 MLLT11→ MIR554→ ←C2CD4D
LOC103091866→ MRPS21→ ←CDC42SE1 LOC100132111→
29 genes omitted
149.5
150
150.5
151
151.5
Position on chr1 (Mb)

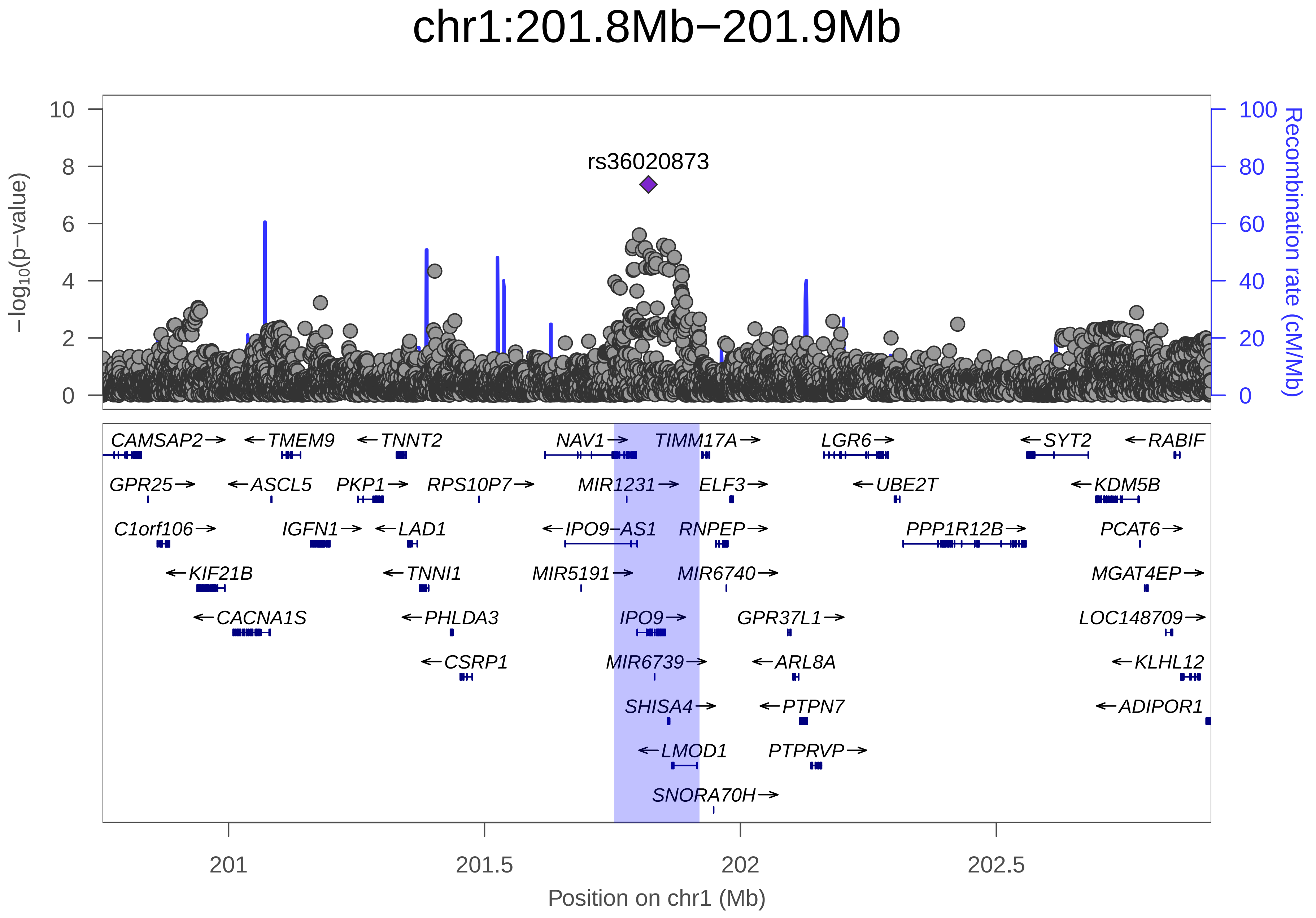

chr1:201.8Mb−201.9Mb
rs36020873
−log10(p−value)
Recombination rate (cM/Mb)
0
2
4
6
8
10
0
20
40
60
80
100
CAMSAP2→
←TMEM9
←TNNT2
NAV1→
TIMM17A→
LGR6→
←SYT2
←RABIF
GPR25→
←ASCL5
PKP1→
RPS10P7→
MIR1231→
ELF3→
←UBE2T
←KDM5B
C1orf106→
IGFN1→
←LAD1
←IPO9−AS1
RNPEP→
PPP1R12B→
PCAT6→
←KIF21B
←TNNI1
MIR5191→
MIR6740→
MGAT4EP→
←CACNA1S
←PHLDA3
IPO9→
GPR37L1→
LOC148709→
←CSRP1
MIR6739→
←ARL8A
←KLHL12
SHISA4→
←PTPN7
←ADIPOR1
←LMOD1
PTPRVP→
SNORA70H→
201
201.5
202
202.5
Position on chr1 (Mb)

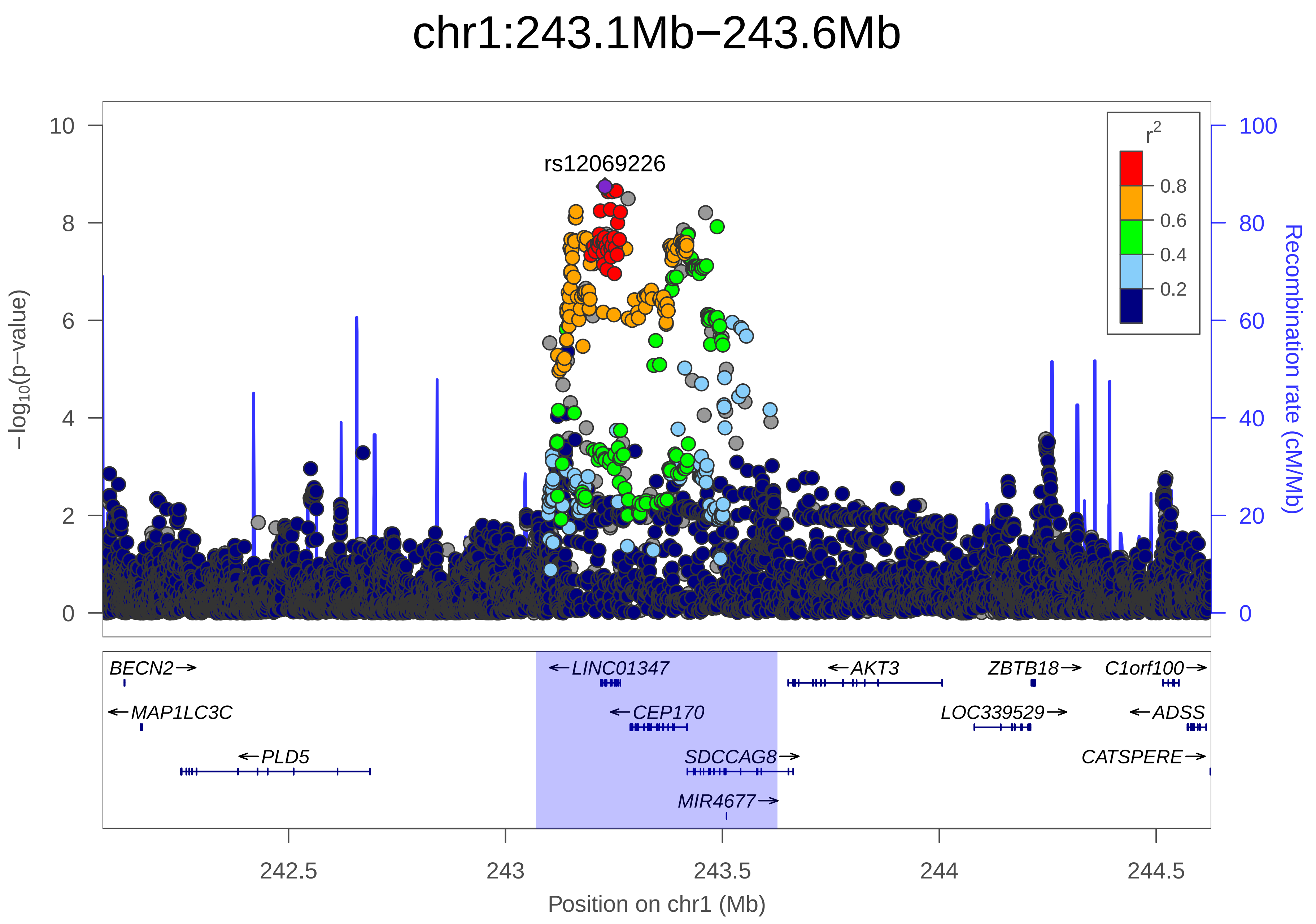

chr1:243.1Mb−243.6Mb
rs12069226
r2
0.8
0.6
0.4
0.2
−log10(p−value)
Recombination rate (cM/Mb)
BECN2
MAP1LC3C
PLD5
LINC01347
CEP170
SDCCAG8
MIR4677
AKT3
ZBTB18
LOC339529
C1orf100
ADSS
CATSPERE
Position on chr1 (Mb)

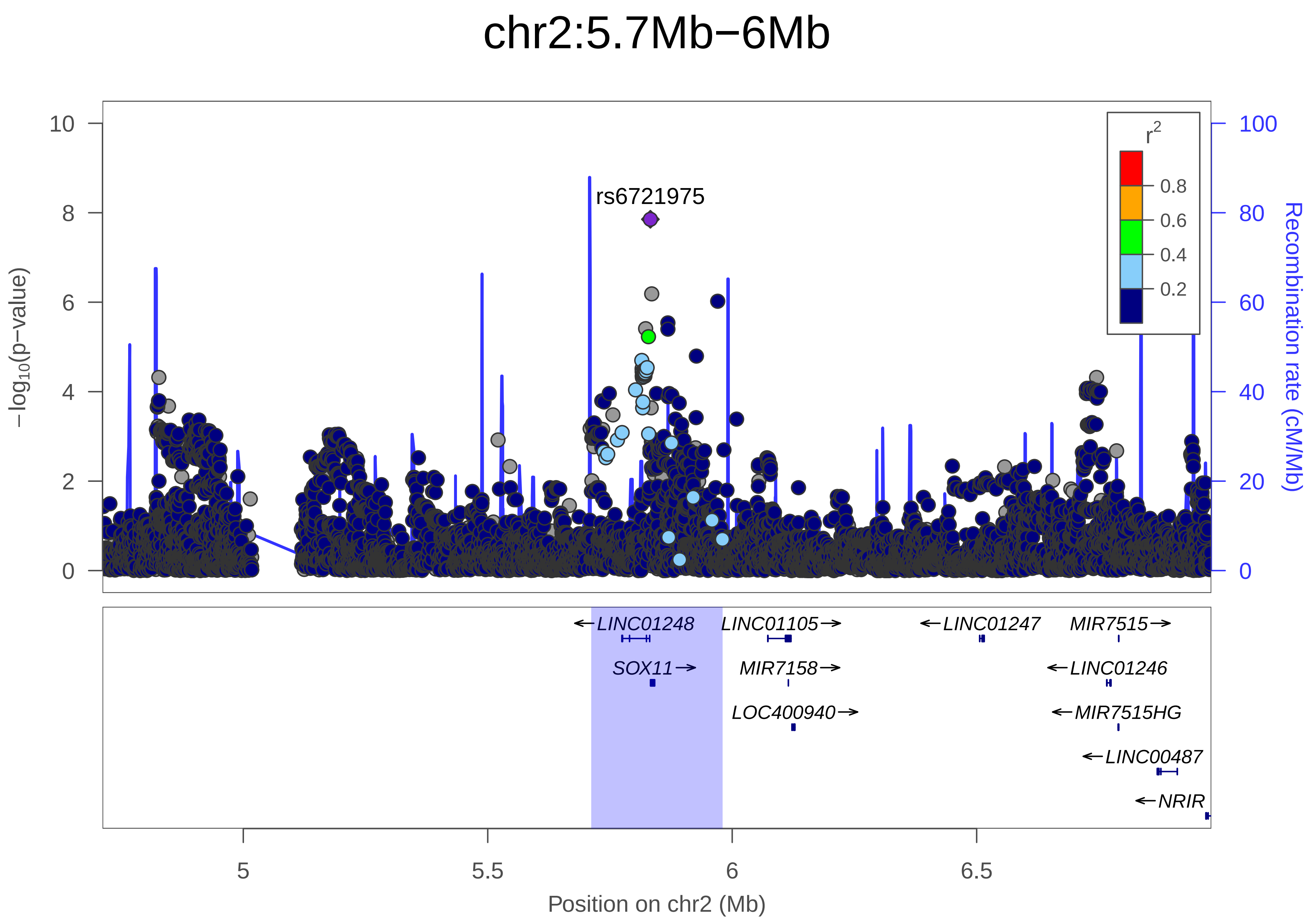

chr2:5.7Mb−6Mb
rs6721975
−log10(p−value)
Recombination rate (cM/Mb)
r2
0.8
0.6
0.4
0.2
10
8
6
4
2
0
100
80
60
40
20
0
LINC01248
LINC01105
LINC01247
MIR7515
SOX11
MIR7158
LINC01246
LOC400940
MIR7515HG
LINC00487
NRIR
5
5.5
6
6.5
Position on chr2 (Mb)

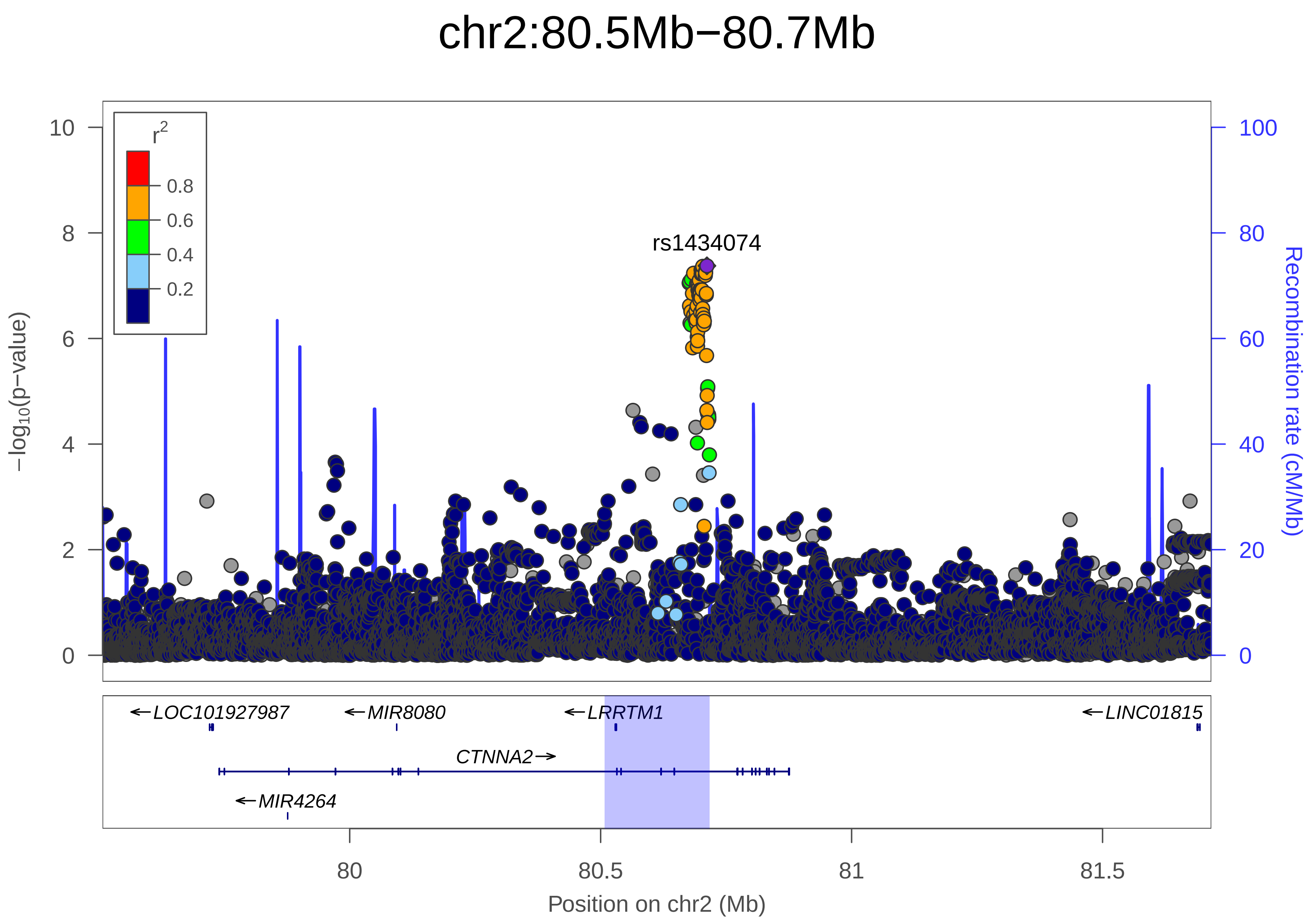
chr2:80.5Mb−80.7Mb
r2
0.8
0.6
0.4
0.2
rs1434074
−log10(p−value)
Recombination rate (cM/Mb)
10
8
6
4
2
0
100
80
60
40
20
0
←LOC101927987
←MIR8080
←LRRTM1
←LINC01815
CTNNA2→
←MIR4264
80
80.5
81
81.5
Position on chr2 (Mb)

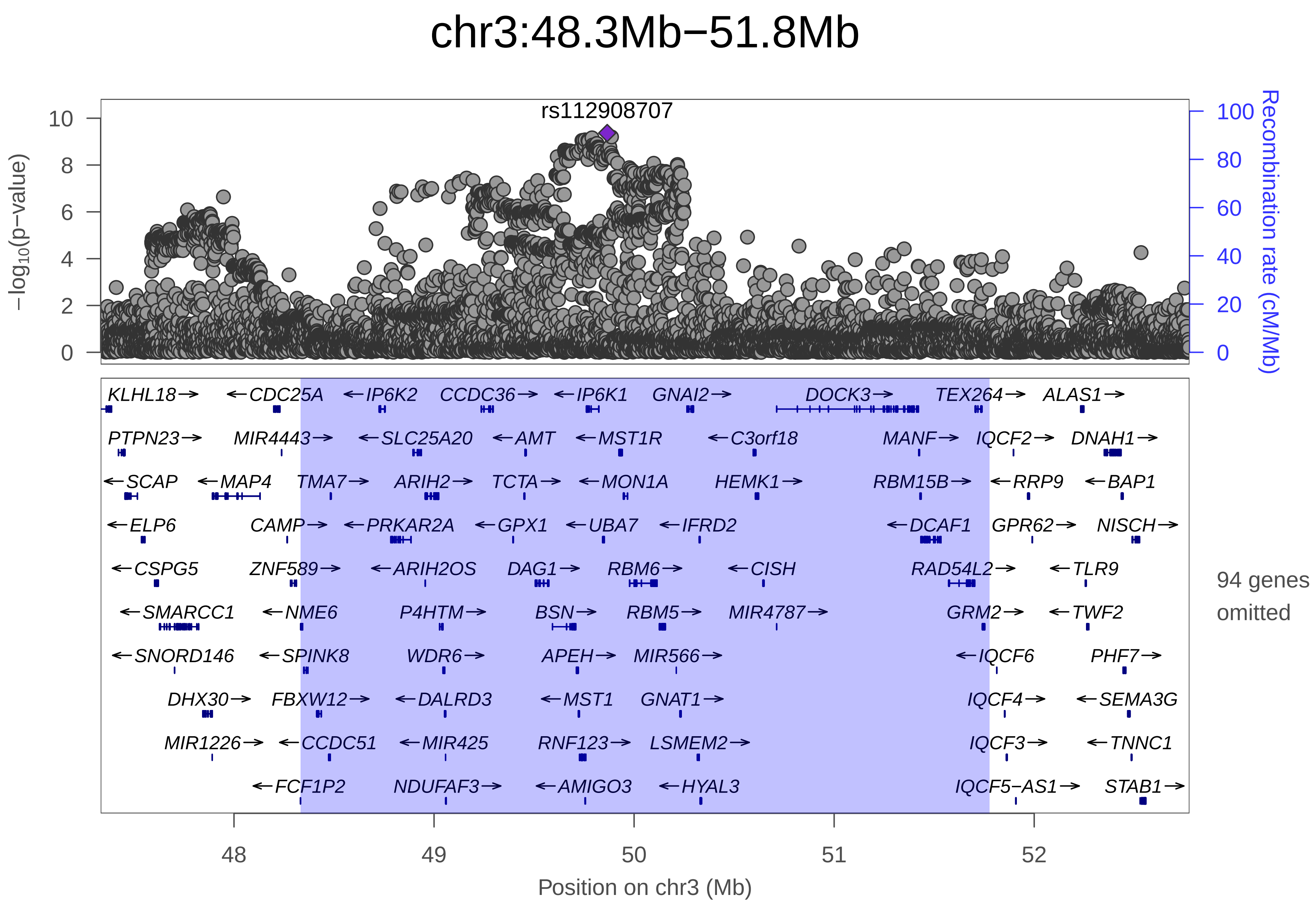

chr3:48.3Mb−51.8Mb
rs112908707
−log10(p−value)
Recombination rate (cM/Mb)
KLHL18→ ←CDC25A ←IP6K2 CCDC36→ ←IP6K1 GNAI2→ DOCK3→ TEX264→ ALAS1→
PTPN23→ MIR4443→ ←SLC25A20 ←AMT ←MST1R ←C3orf18 MANF→ IQCF2→ DNAH1→
←SCAP ←MAP4 TMA7→ ARIH2→ TCTA→ ←MON1A HEMK1→ RBM15B→ ←RRP9 ←BAP1
←ELP6 CAMP→ ←PRKAR2A ←GPX1 ←UBA7 ←IFRD2 ←DCAF1 GPR62→ NISCH→
←CSPG5 ZNF589→ ←ARIH2OS DAG1→ RBM6→ ←CISH RAD54L2→ ←TLR9
←SMARCC1 ←NME6 P4HTM→ BSN→ RBM5→ MIR4787→ GRM2→ ←TWF2
←SNORD146 ←SPINK8 WDR6→ APEH→ MIR566→ ←IQCF6 PHF7→
DHX30→ FBXW12→ ←DALRD3 ←MST1 GNAT1→ IQCF4→ ←SEMA3G
MIR1226→ ←CCDC51 ←MIR425 RNF123→ LSMEM2→ IQCF3→ ←TNNC1
←FCF1P2 NDUFAF3→ ←AMIGO3 ←HYAL3 IQCF5−AS1→ STAB1→
94 genes omitted
Position on chr3 (Mb)

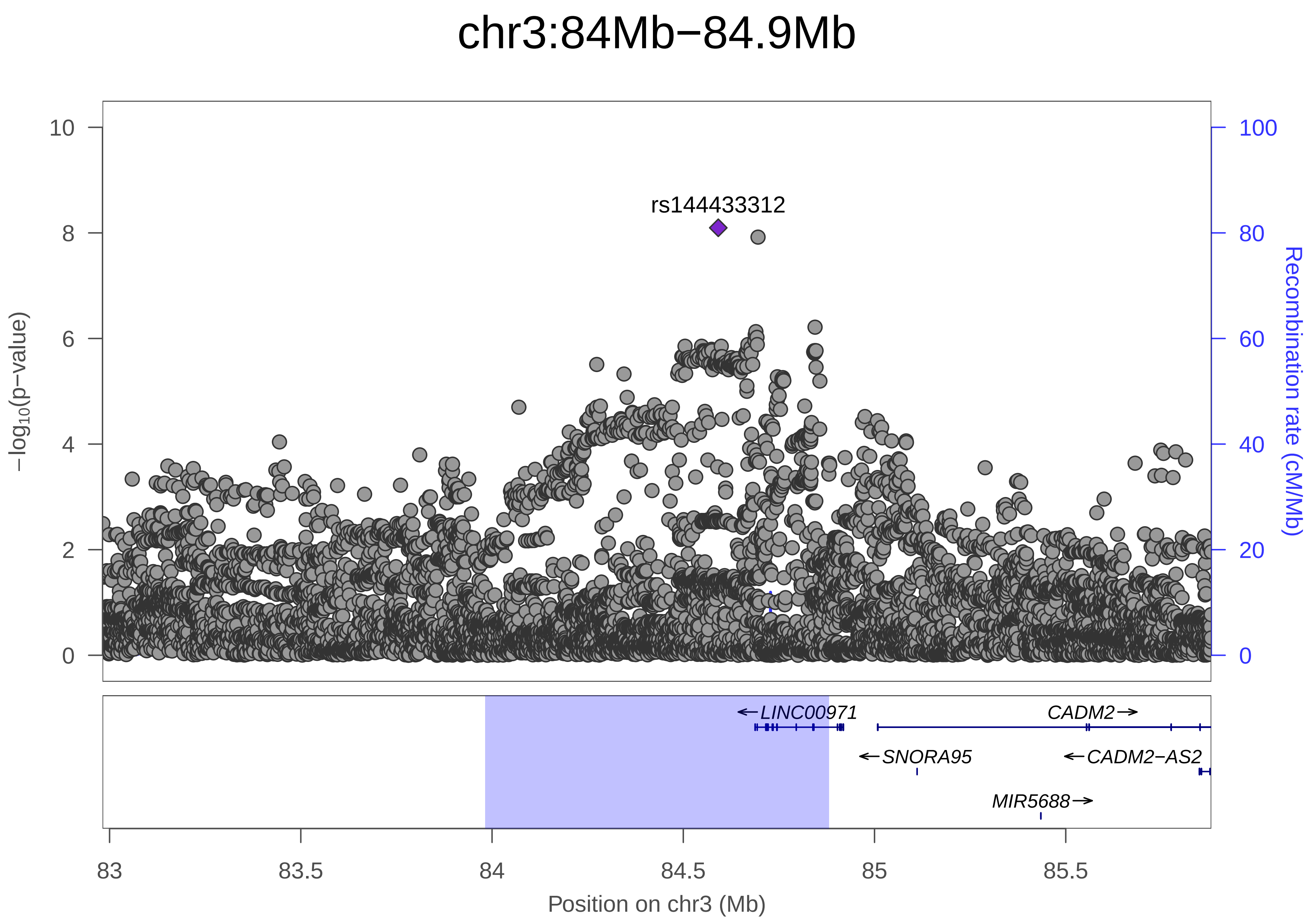

chr3:84Mb−84.9Mb
rs144433312
−log10(p−value)
Recombination rate (cM/Mb)
10
8
6
4
2
0
100
80
60
40
20
0
←LINC00971
CADM2→
←SNORA95
←CADM2−AS2
MIR5688→
83
83.5
84
84.5
85
85.5
Position on chr3 (Mb)

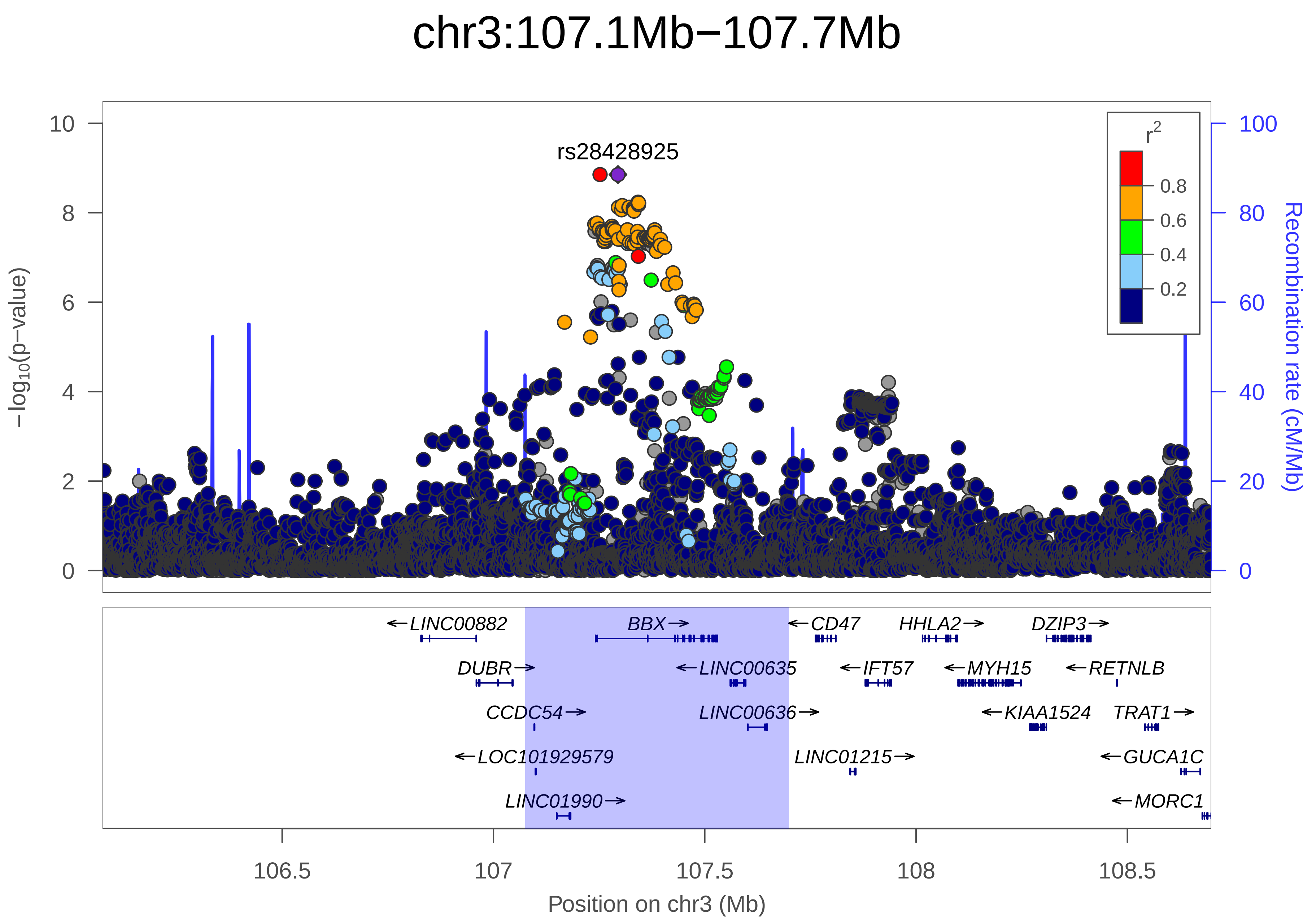

chr3:107.1Mb−107.7Mb
rs28428925
r2
0.8
0.6
0.4
0.2
−log10(p−value)
Recombination rate (cM/Mb)
0
2
4
6
8
10
0
20
40
60
80
100
LINC00882
BBX
CD47
HHLA2
DZIP3
DUBR
LINC00635
IFT57
MYH15
RETNLB
CCDC54
LINC00636
KIAA1524
TRAT1
LOC101929579
LINC01215
GUCA1C
LINC01990
MORC1
106.5
107
107.5
108
108.5
Position on chr3 (Mb)

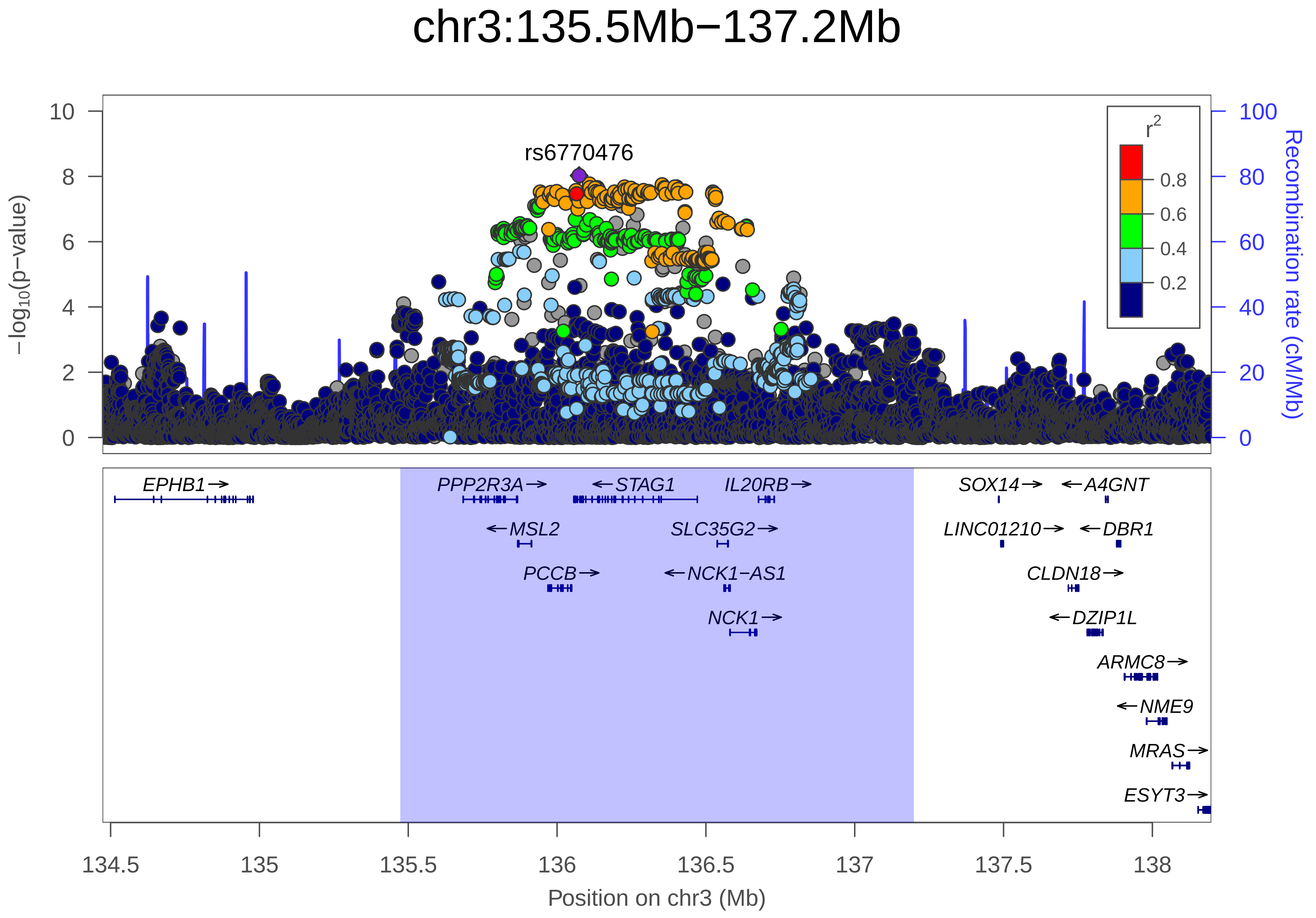
chr3:135.5Mb−137.2Mb
rs6770476
−log10(p−value)
Recombination rate (cM/Mb)
r2
0.8
0.6
0.4
0.2
EPHB1→
PPP2R3A→
←STAG1
IL20RB→
SOX14→
←A4GNT
←MSL2
SLC35G2→
LINC01210→
←DBR1
PCCB→
←NCK1−AS1
CLDN18→
NCK1→
←DZIP1L
ARMC8→
←NME9
MRAS→
ESYT3→
Position on chr3 (Mb)

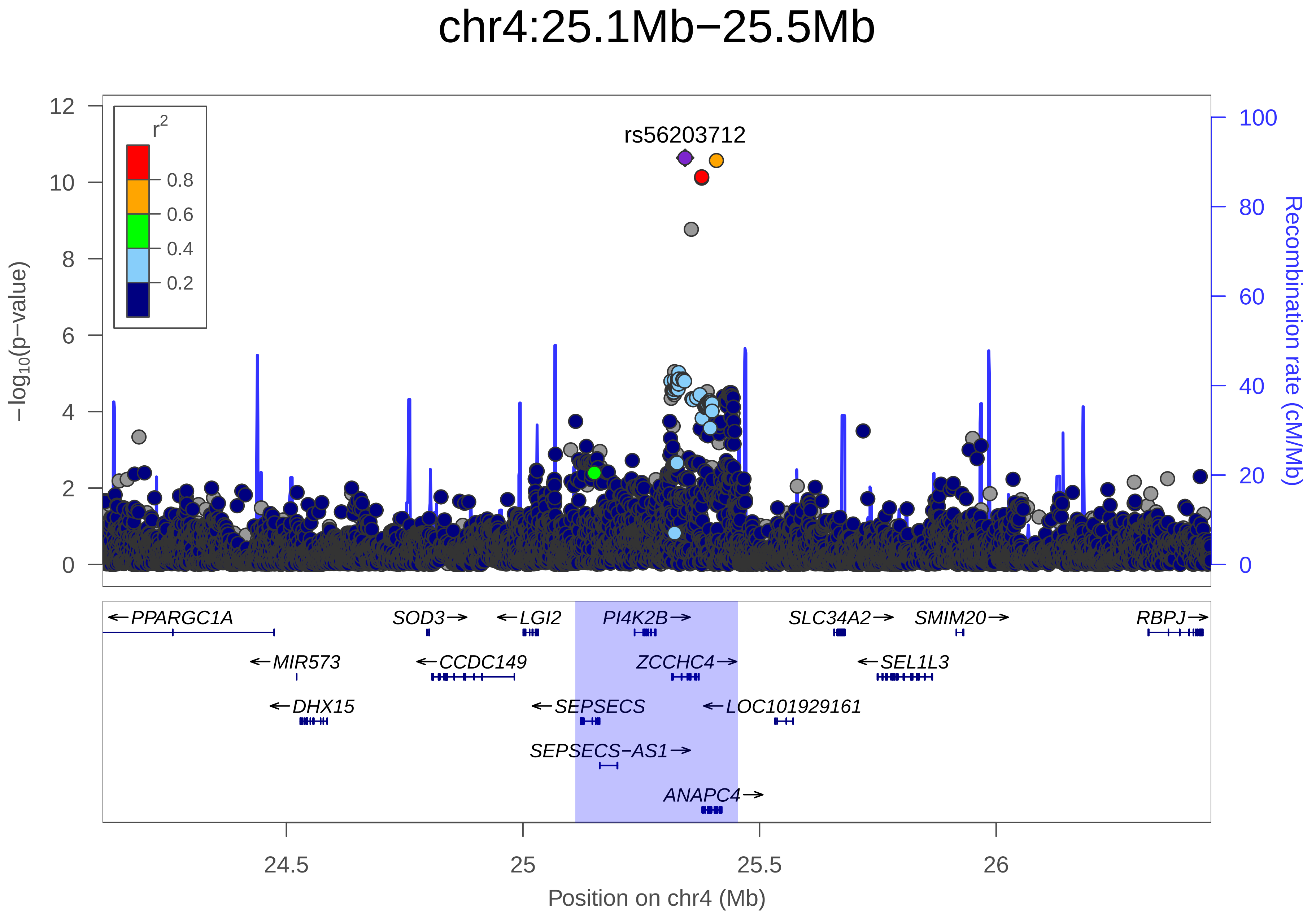

chr4:25.1Mb−25.5Mb
rs56203712
r2
0.8
0.6
0.4
0.2
12
10
8
6
4
2
0
−log10(p−value)
100
80
60
40
20
0
Recombination rate (cM/Mb)
PPARGC1A
SOD3
LGI2
PI4K2B
SLC34A2
SMIM20
RBPJ
MIR573
CCDC149
ZCCHC4
SEL1L3
DHX15
SEPSECS
LOC101929161
SEPSECS−AS1
ANAPC4
24.5
25
25.5
26
Position on chr4 (Mb)

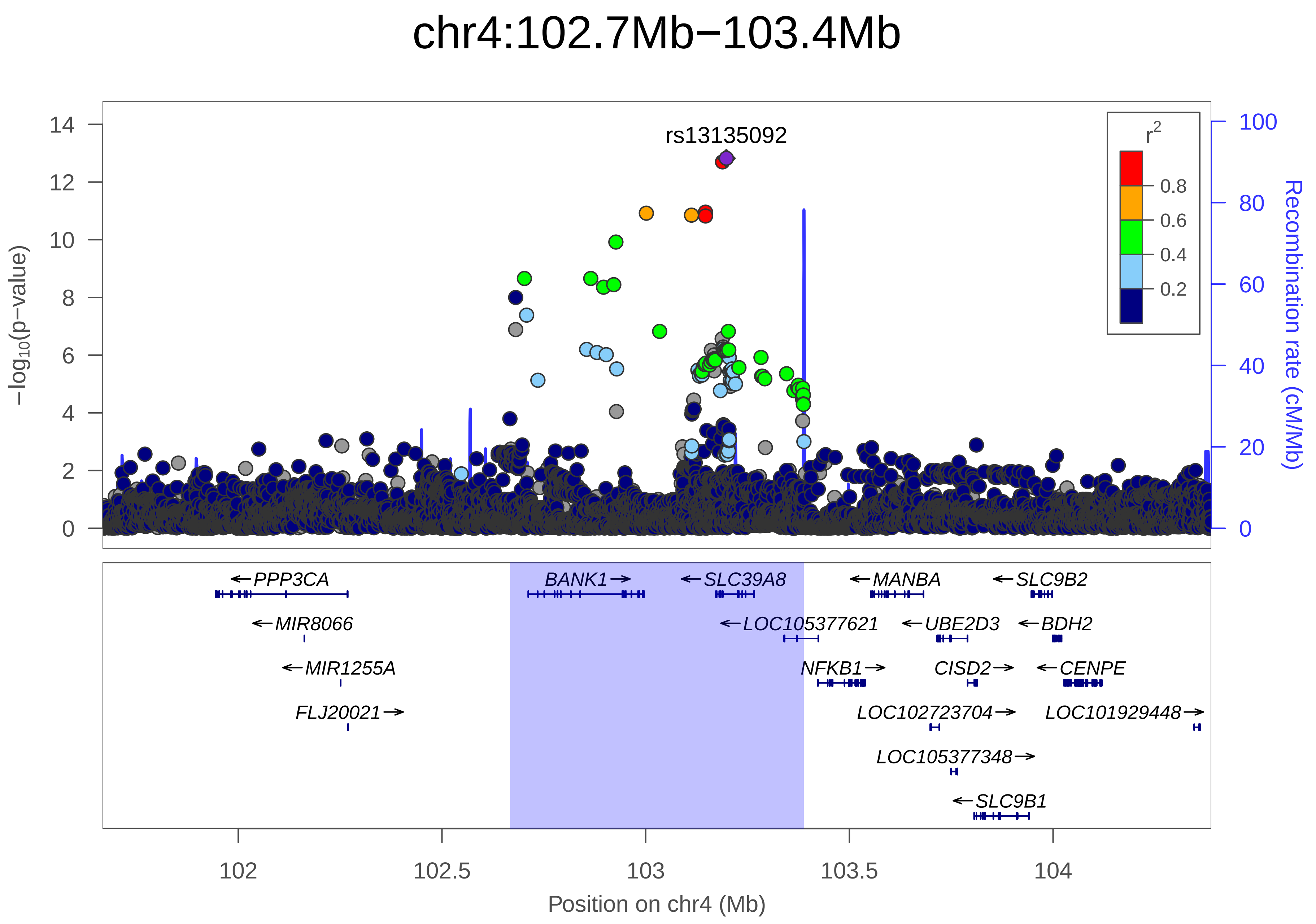

chr4:102.7Mb−103.4Mb
rs13135092
r2
0.8
0.6
0.4
0.2
−log10(p−value)
Recombination rate (cM/Mb)
14
12
10
8
6
4
2
0
100
80
60
40
20
0
PPP3CA
BANK1
SLC39A8
MANBA
SLC9B2
MIR8066
LOC105377621
UBE2D3
BDH2
MIR1255A
NFKB1
CISD2
CENPE
FLJ20021
LOC102723704
LOC101929448
LOC105377348
SLC9B1
102
102.5
103
103.5
104
Position on chr4 (Mb)

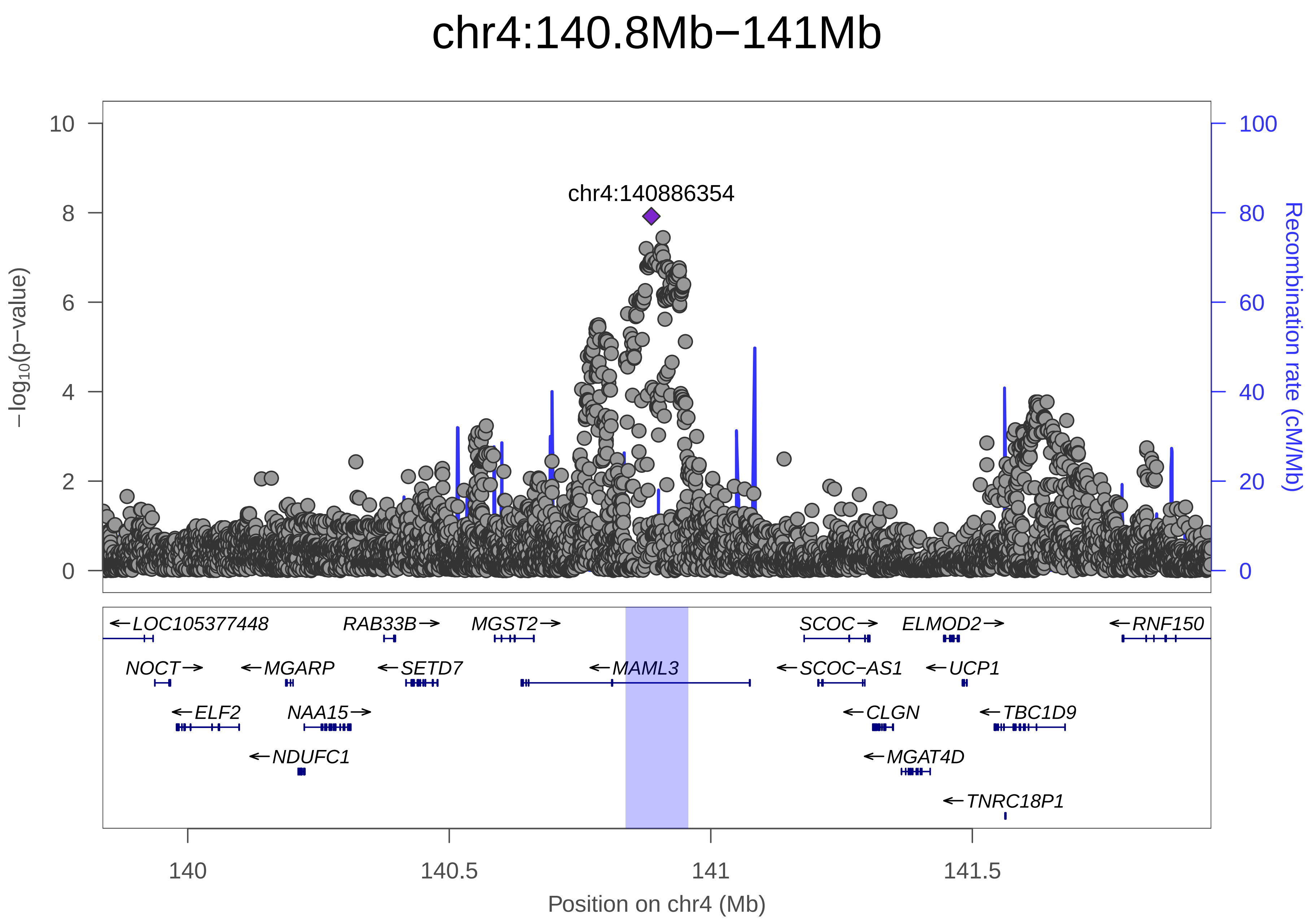

chr4:140.8Mb−141Mb
chr4:140886354
−log10(p−value)
Recombination rate (cM/Mb)
0
2
4
6
8
10
0
20
40
60
80
100
LOC105377448
RAB33B
MGST2
SCOC
ELMOD2
RNF150
NOCT
MGARP
SETD7
MAML3
SCOC−AS1
UCP1
ELF2
NAA15
CLGN
TBC1D9
NDUFC1
MGAT4D
TNRC18P1
140
140.5
141
141.5
Position on chr4 (Mb)

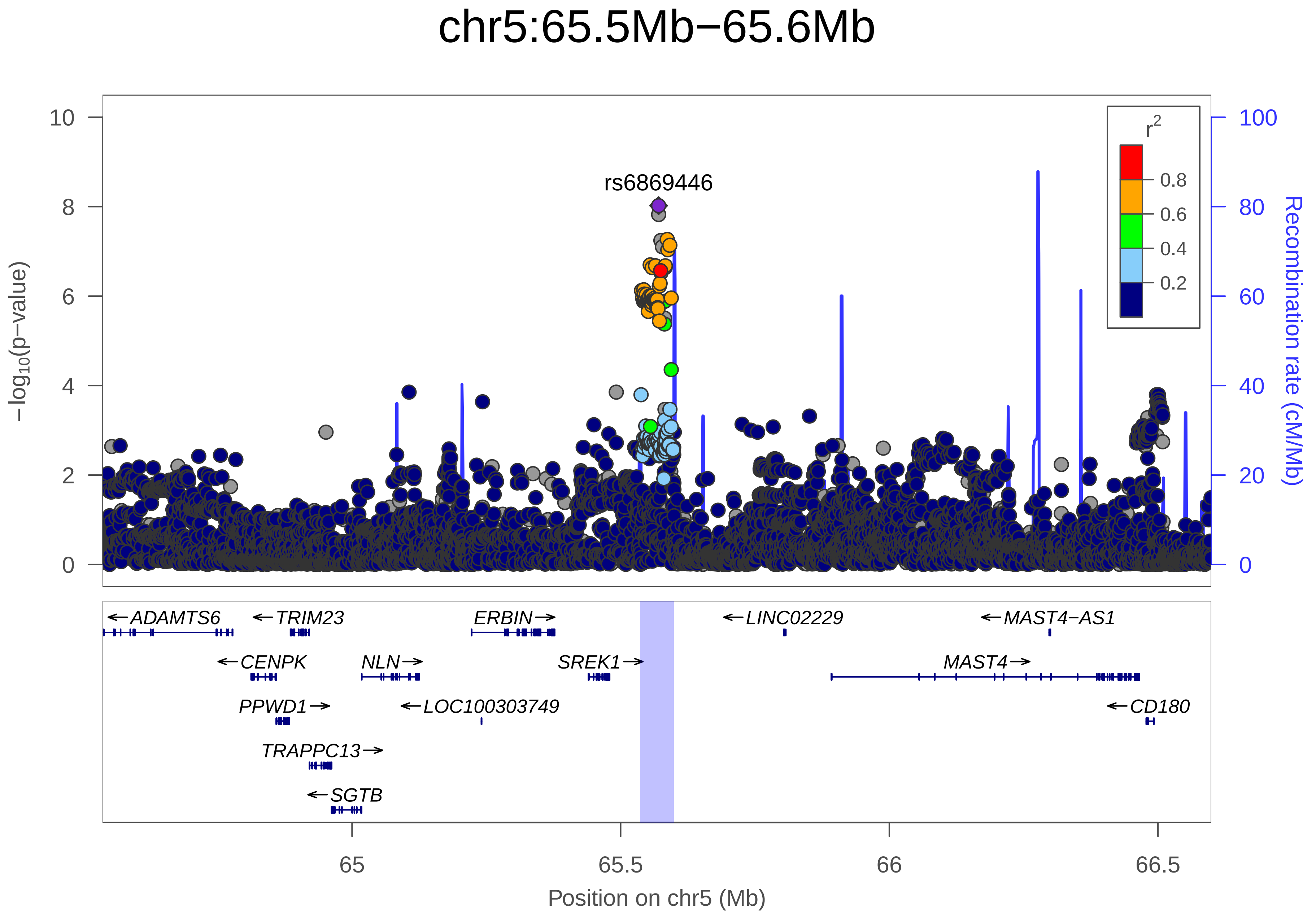
chr5:65.5Mb−65.6Mb
rs6869446
r2
0.8
0.6
0.4
0.2
−log10(p−value)
Recombination rate (cM/Mb)
Position on chr5 (Mb)
ADAMTS6
TRIM23
ERBIN
LINC02229
MAST4−AS1
CENPK
NLN
SREK1
MAST4
PPWD1
LOC100303749
CD180
TRAPPC13
SGTB

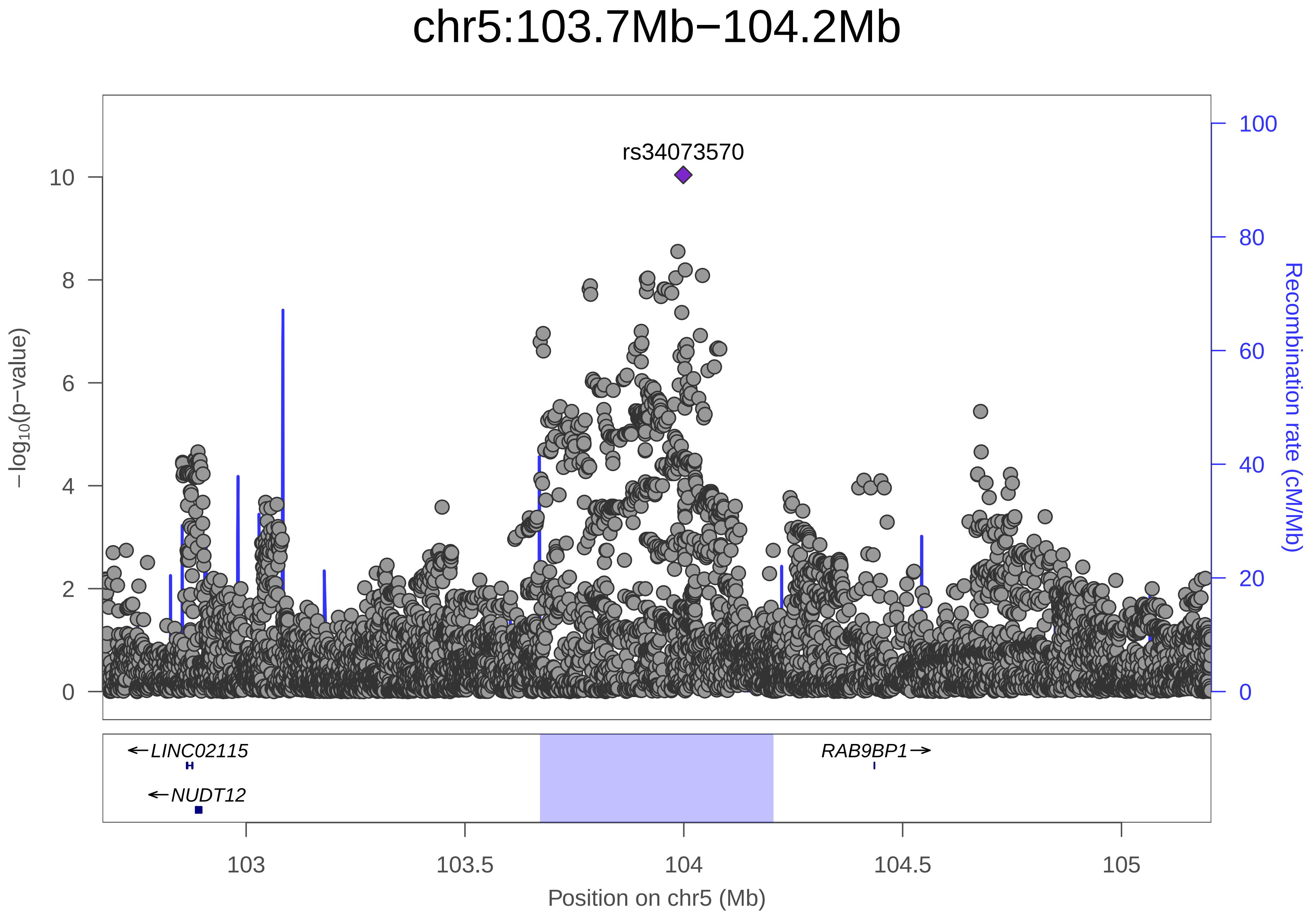

chr5:103.7Mb−104.2Mb
rs34073570
−log10(p−value)
Recombination rate (cM/Mb)
10
8
6
4
2
0
100
80
60
40
20
0
←LINC02115
←NUDT12
RAB9BP1→
103
103.5
104
104.5
105
Position on chr5 (Mb)

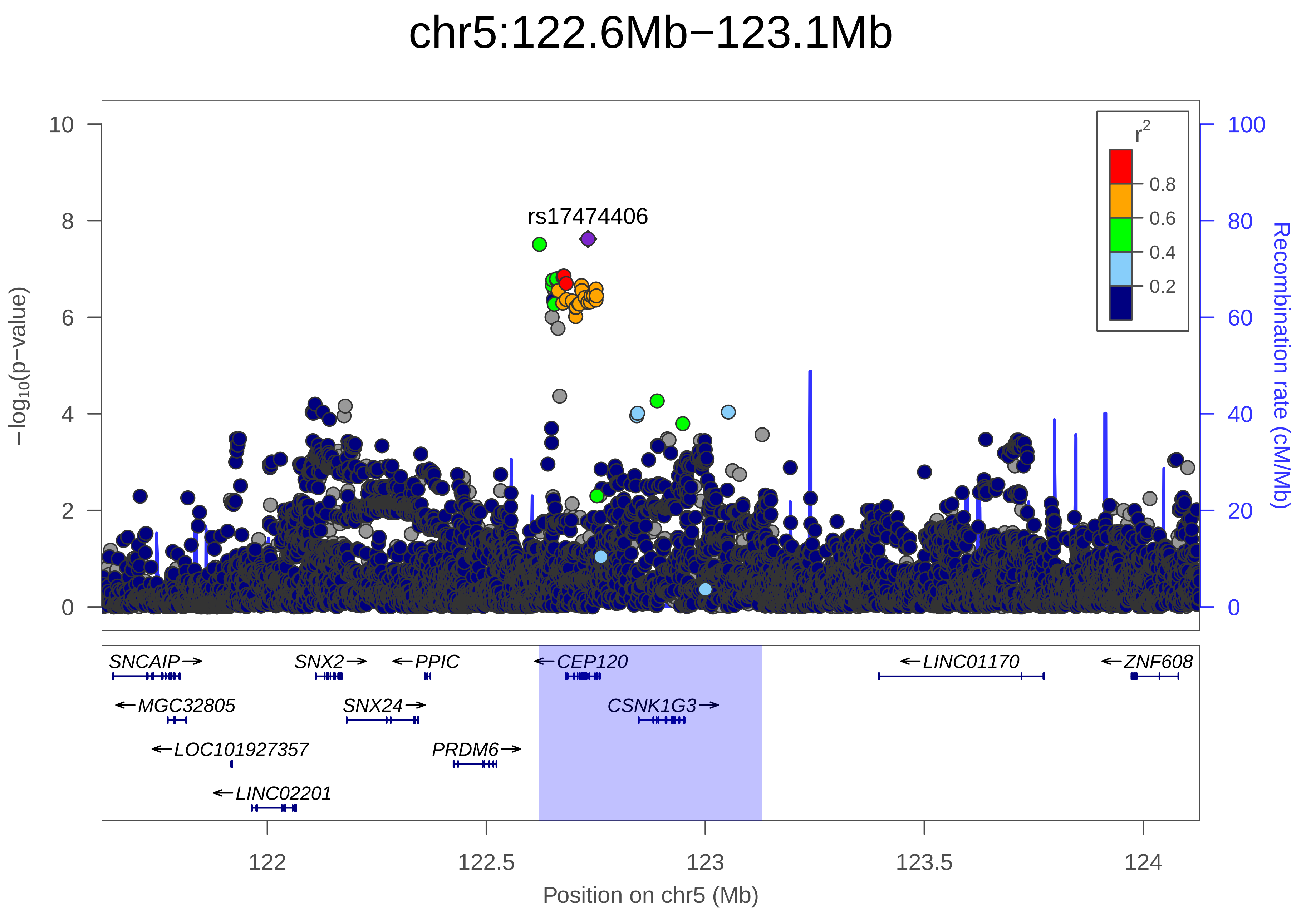
chr5:122.6Mb−123.1Mb
rs17474406
−log10(p−value)
Recombination rate (cM/Mb)
r2
0.8
0.6
0.4
0.2
0
2
4
6
8
10
0
20
40
60
80
100
SNCAIP
MGC32805
LOC101927357
LINC02201
SNX2
SNX24
PPIC
PRDM6
CEP120
CSNK1G3
LINC01170
ZNF608
122
122.5
123
123.5
124
Position on chr5 (Mb)

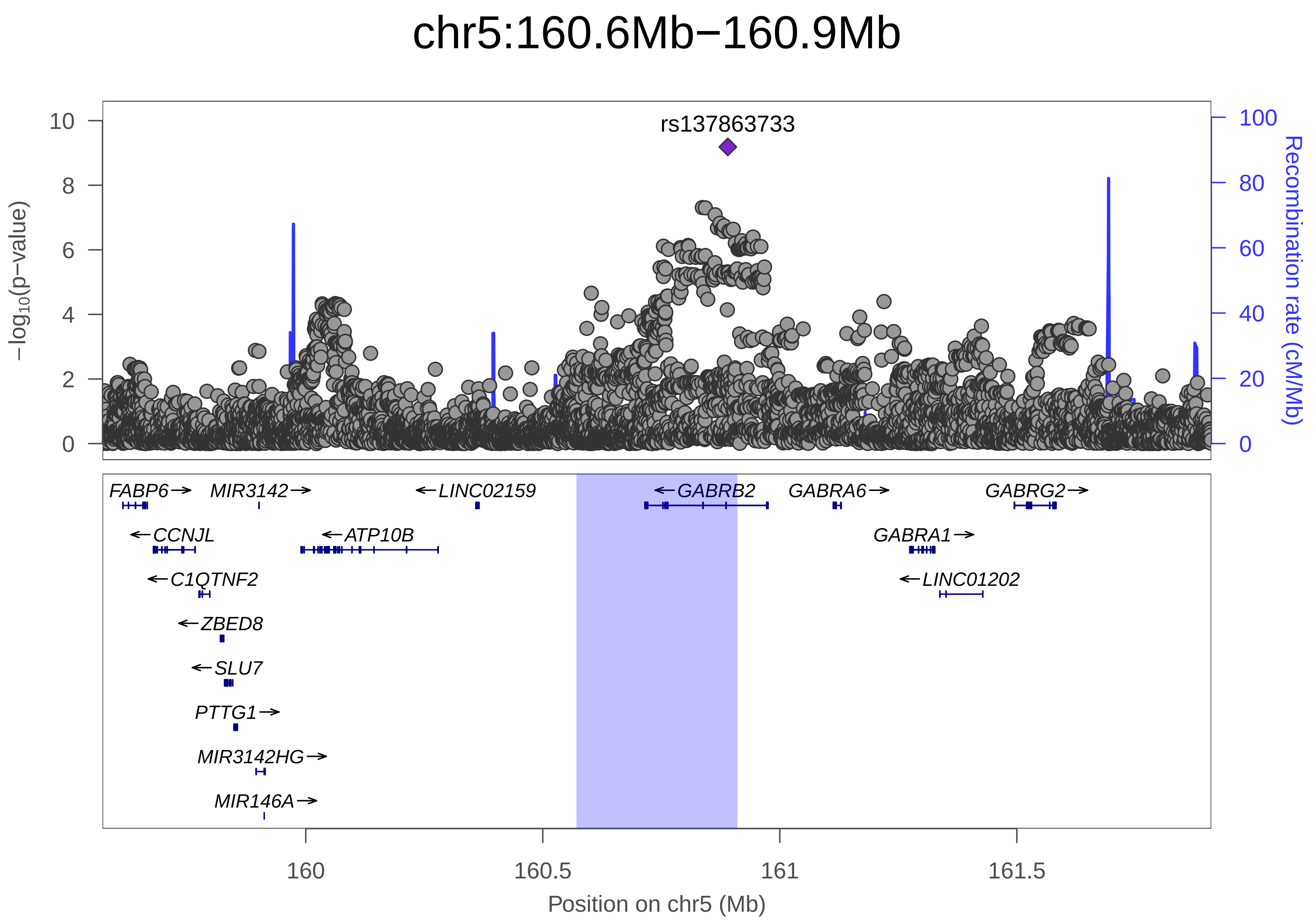
chr5:160.6Mb−160.9Mb
rs137863733
−log10(p−value)
Recombination rate (cM/Mb)
0
2
4
6
8
10
0
20
40
60
80
100
FABP6
MIR3142
LINC02159
GABRB2
GABRA6
GABRG2
CCNJL
ATP10B
GABRA1
C1QTNF2
LINC01202
ZBED8
SLU7
PTTG1
MIR3142HG
MIR146A
160
160.5
161
161.5
Position on chr5 (Mb)

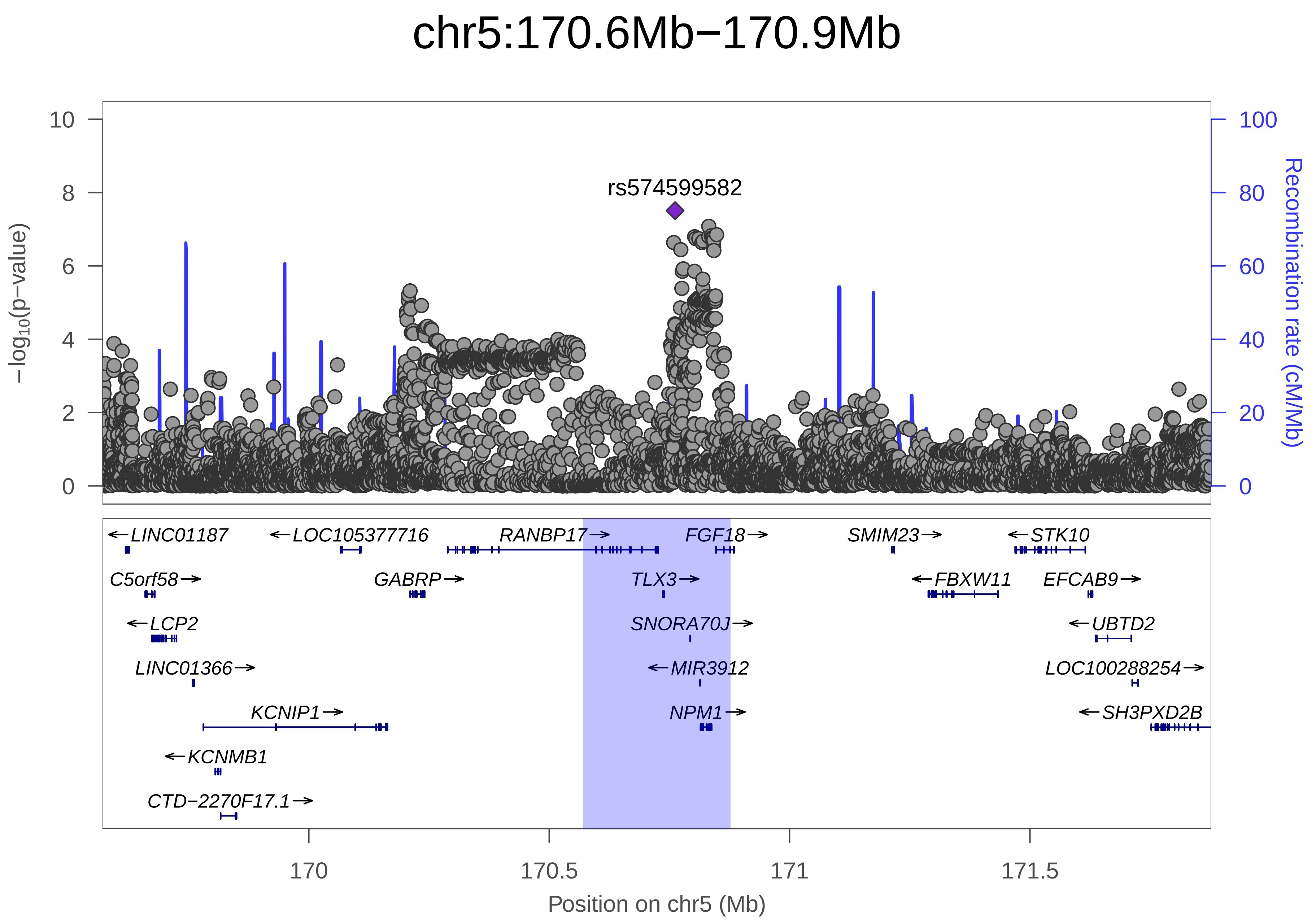

chr5:170.6Mb−170.9Mb
rs574599582
−log10(p−value)
Recombination rate (cM/Mb)
10
8
6
4
2
0
100
80
60
40
20
0
LINC01187
LOC105377716
RANBP17
FGF18
SMIM23
STK10
C5orf58
GABRP
TLX3
FBXW11
EFCAB9
LCP2
SNORA70J
UBTD2
LINC01366
MIR3912
LOC100288254
KCNIP1
NPM1
SH3PXD2B
KCNMB1
CTD−2270F17.1
170
170.5
171
171.5
Position on chr5 (Mb)

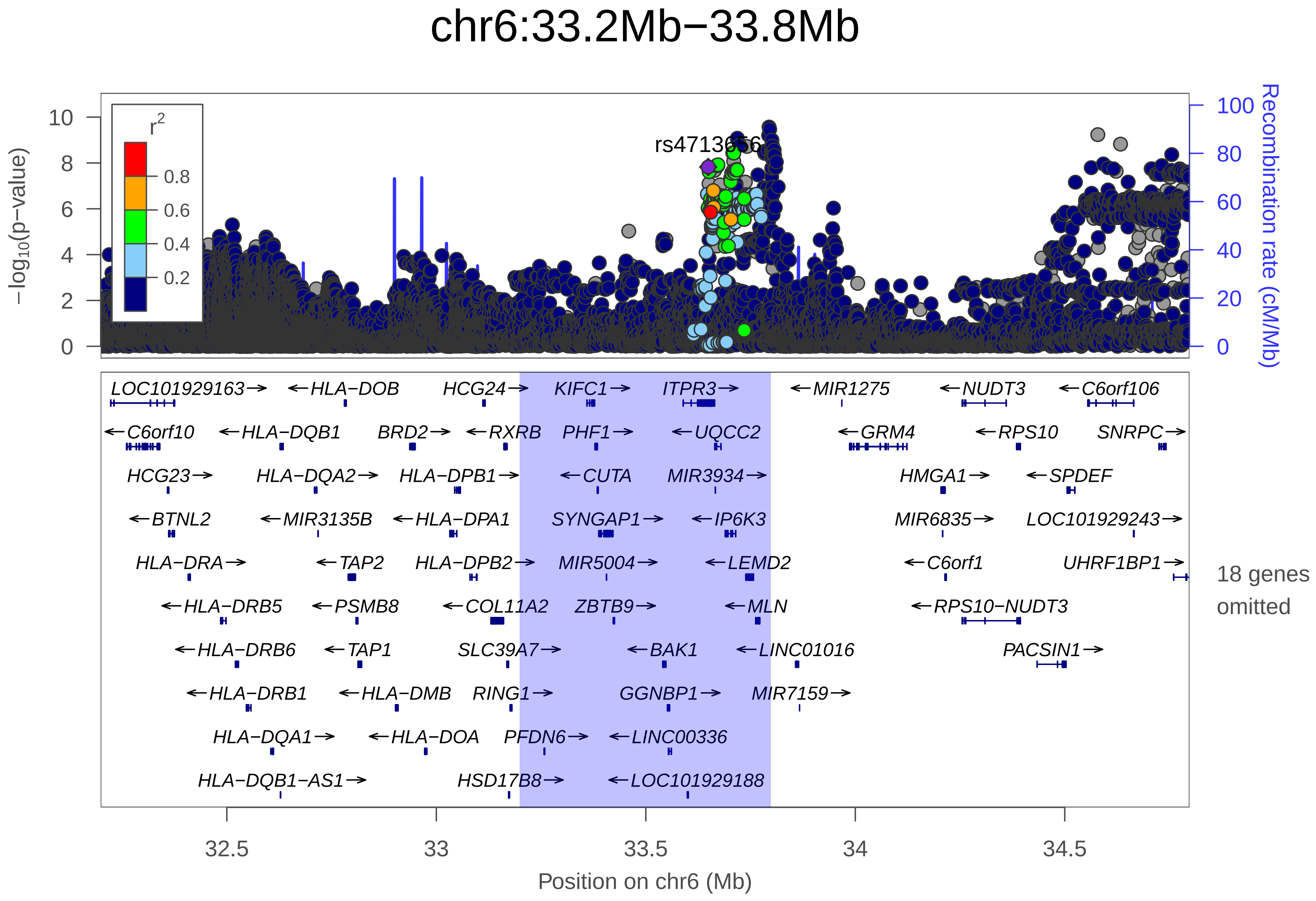

chr6:33.2Mb−33.8Mb
rs4713656
r2
0.8
0.6
0.4
0.2
−log10(p−value)
Recombination rate (cM/Mb)
LOC101929163→ ←HLA−DOB HCG24→ KIFC1→ ITPR3→ ←MIR1275 ←NUDT3 ←C6orf106
←C6orf10 ←HLA−DQB1 BRD2→ ←RXRB PHF1→ ←UQCC2 ←GRM4 ←RPS10 SNRPC→
HCG23→ HLA−DQA2→ HLA−DPB1→ ←CUTA MIR3934→ HMGA1→ ←SPDEF
←BTNL2 ←MIR3135B ←HLA−DPA1 SYNGAP1→ ←IP6K3 MIR6835→ LOC101929243→
HLA−DRA→ ←TAP2 HLA−DPB2→ MIR5004→ ←LEMD2 ←C6orf1 UHRF1BP1→
←HLA−DRB5 ←PSMB8 ←COL11A2 ZBTB9→ ←MLN ←RPS10−NUDT3
←HLA−DRB6 ←TAP1 SLC39A7→ ←BAK1 ←LINC01016 PACSIN1→
←HLA−DRB1 ←HLA−DMB RING1→ GGNBP1→ MIR7159→
HLA−DQA1→ ←HLA−DOA PFDN6→ ←LINC00336
HLA−DQB1−AS1→ HSD17B8→ ←LOC101929188
18 genes omitted
32.5
33
33.5
34
34.5
Position on chr6 (Mb)

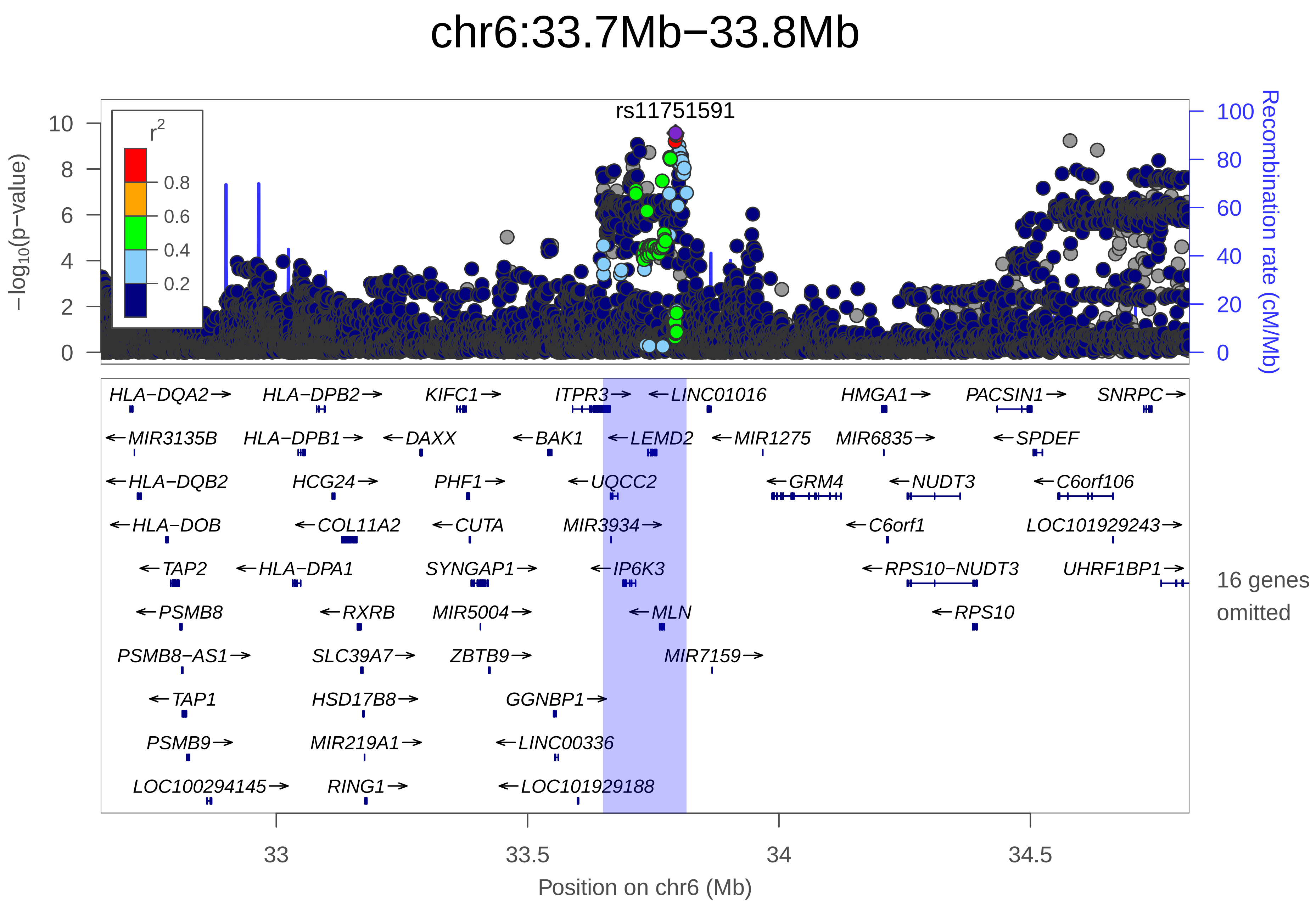

chr6:33.7Mb−33.8Mb
rs11751591
r2
0.8
0.6
0.4
0.2
−log10(p−value)
Recombination rate (cM/Mb)
HLA−DQA2→
HLA−DPB2→
KIFC1→
ITPR3→
←LINC01016
HMGA1→
PACSIN1→
SNRPC→
←MIR3135B
HLA−DPB1→
←DAXX
←BAK1
←LEMD2
←MIR1275
MIR6835→
←SPDEF
←HLA−DQB2
HCG24→
PHF1→
←UQCC2
←GRM4
←NUDT3
←C6orf106
←HLA−DOB
←COL11A2
←CUTA
MIR3934→
←C6orf1
LOC101929243→
←TAP2
←HLA−DPA1
SYNGAP1→
←IP6K3
←RPS10−NUDT3
UHRF1BP1→
16 genes omitted
←PSMB8
←RXRB
MIR5004→
←MLN
←RPS10
PSMB8−AS1→
SLC39A7→
ZBTB9→
MIR7159→
←TAP1
HSD17B8→
GGNBP1→
PSMB9→
MIR219A1→
←LINC00336
LOC100294145→
RING1→
←LOC101929188
Position on chr6 (Mb)

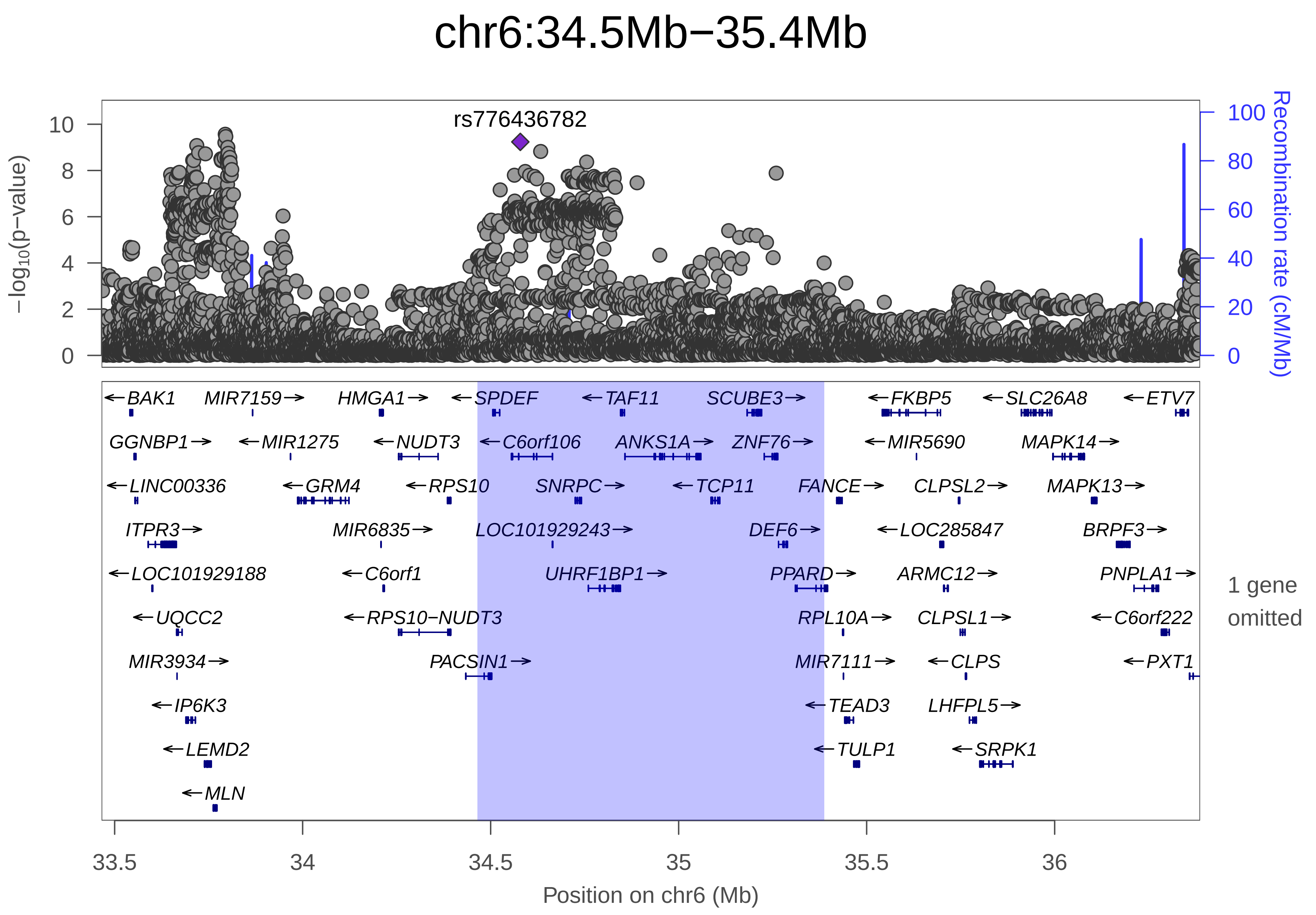
chr6:34.5Mb−35.4Mb
rs776436782
−log10(p−value)
Recombination rate (cM/Mb)
0
2
4
6
8
10
0
20
40
60
80
100
←BAK1
MIR7159→
HMGA1→
←SPDEF
←TAF11
SCUBE3→
←FKBP5
←SLC26A8
←ETV7
GGNBP1→
←MIR1275
←NUDT3
←C6orf106
ANKS1A→
ZNF76→
←MIR5690
MAPK14→
←LINC00336
←GRM4
←RPS10
SNRPC→
←TCP11
FANCE→
CLPSL2→
MAPK13→
ITPR3→
MIR6835→
LOC101929243→
DEF6→
←LOC285847
BRPF3→
←LOC101929188
←C6orf1
UHRF1BP1→
PPARD→
ARMC12→
PNPLA1→
1 gene omitted
←UQCC2
←RPS10−NUDT3
RPL10A→
CLPSL1→
←C6orf222
MIR3934→
PACSIN1→
MIR7111→
←CLPS
←PXT1
←IP6K3
←TEAD3
LHFPL5→
←LEMD2
←TULP1
←SRPK1
←MLN
33.5
34
34.5
35
35.5
36
Position on chr6 (Mb)

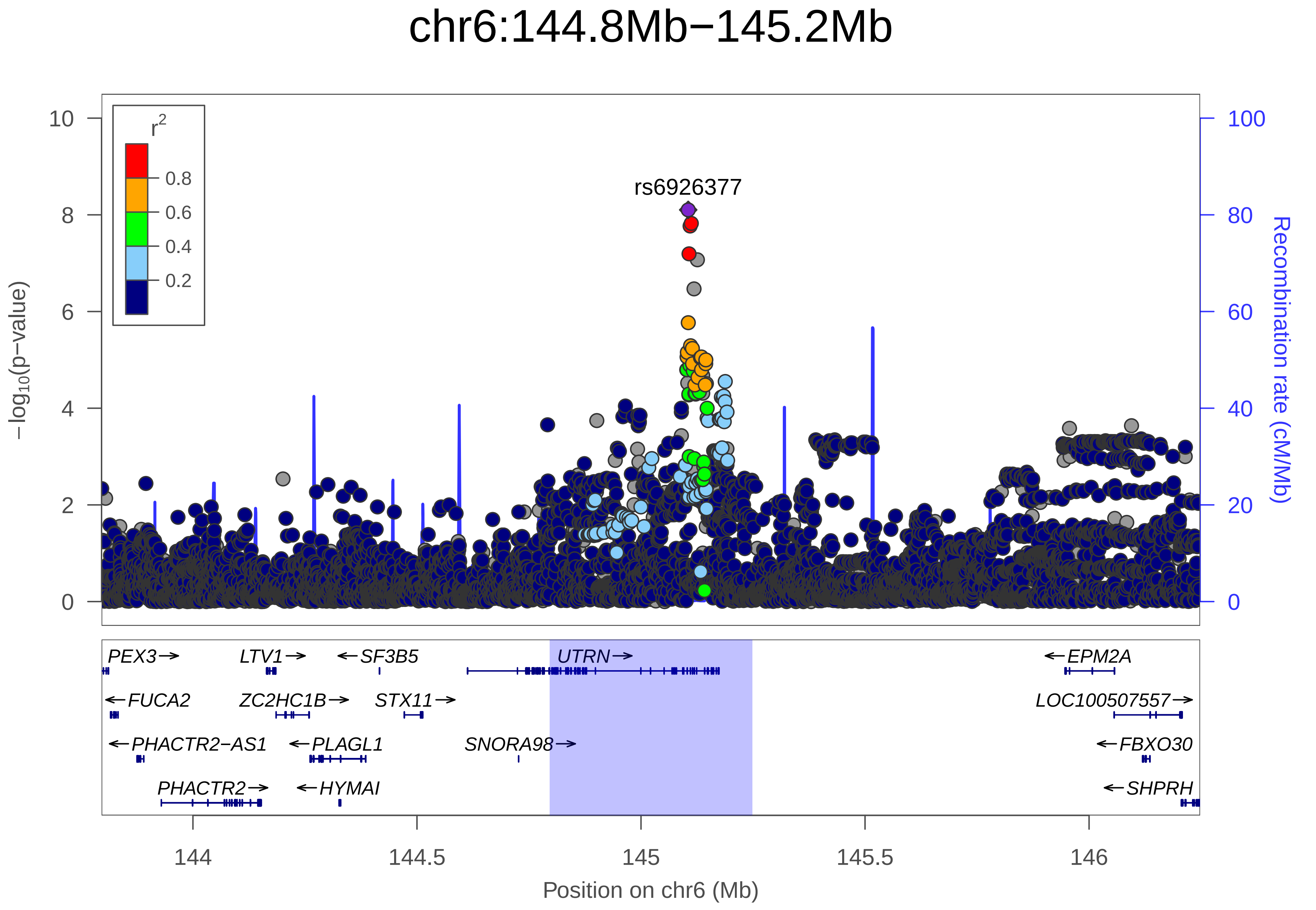

chr6:144.8Mb−145.2Mb
r2
0.8
0.6
0.4
0.2
rs6926377
−log10(p−value)
Recombination rate (cM/Mb)
PEX3→
LTV1→
←SF3B5
UTRN→
←EPM2A
←FUCA2
ZC2HC1B→
STX11→
LOC100507557→
←PHACTR2−AS1
←PLAGL1
SNORA98→
←FBXO30
PHACTR2→
←HYMAI
←SHPRH
144
144.5
145
145.5
146
Position on chr6 (Mb)

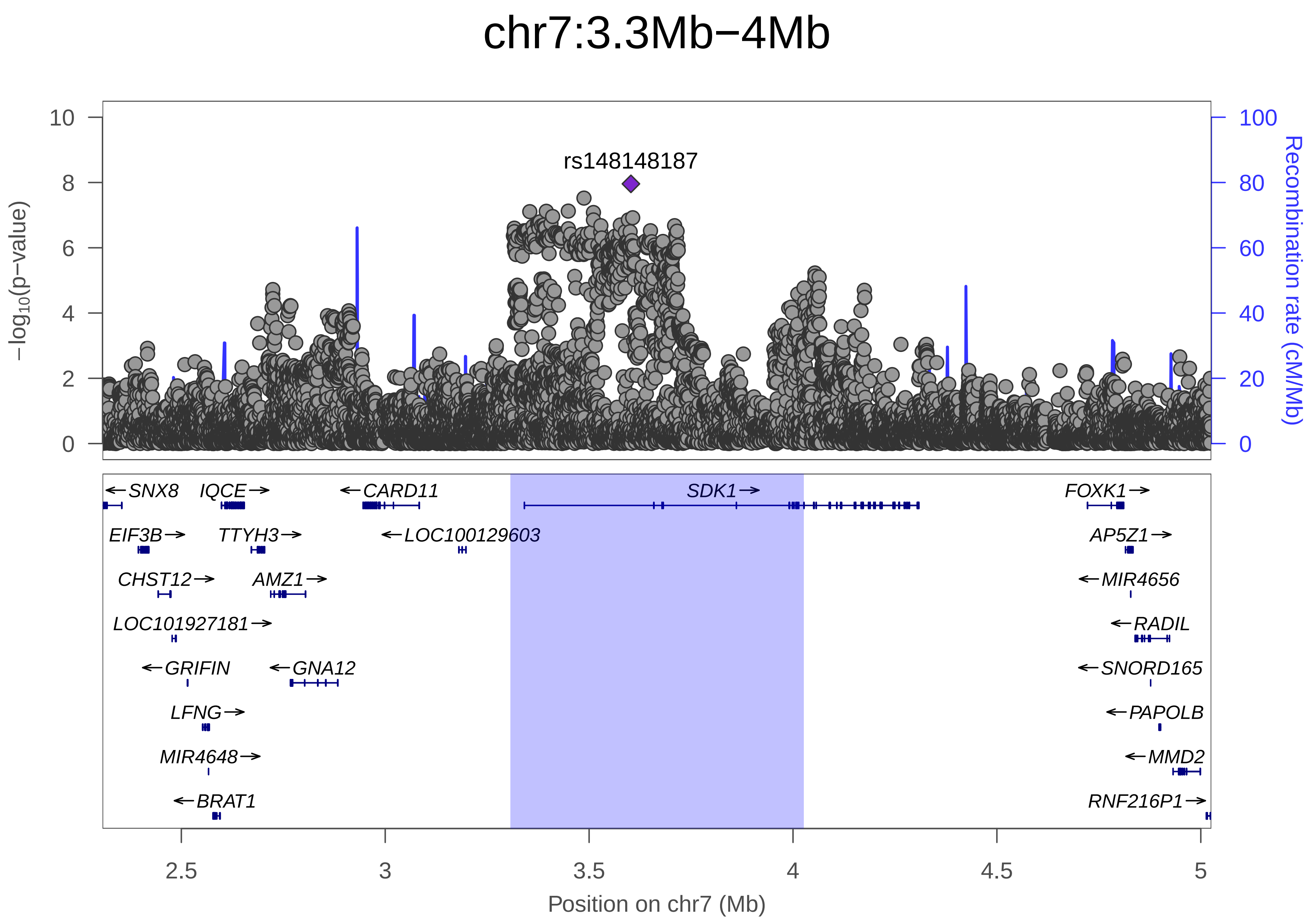

chr7:3.3Mb−4Mb
rs148148187
−log10(p−value)
Recombination rate (cM/Mb)
SNX8
IQCE
CARD11
SDK1
FOXK1
EIF3B
TTYH3
LOC100129603
AP5Z1
CHST12
AMZ1
MIR4656
LOC101927181
RADIL
GRIFIN
GNA12
SNORD165
LFNG
PAPOLB
MIR4648
MMD2
BRAT1
RNF216P1
Position on chr7 (Mb)

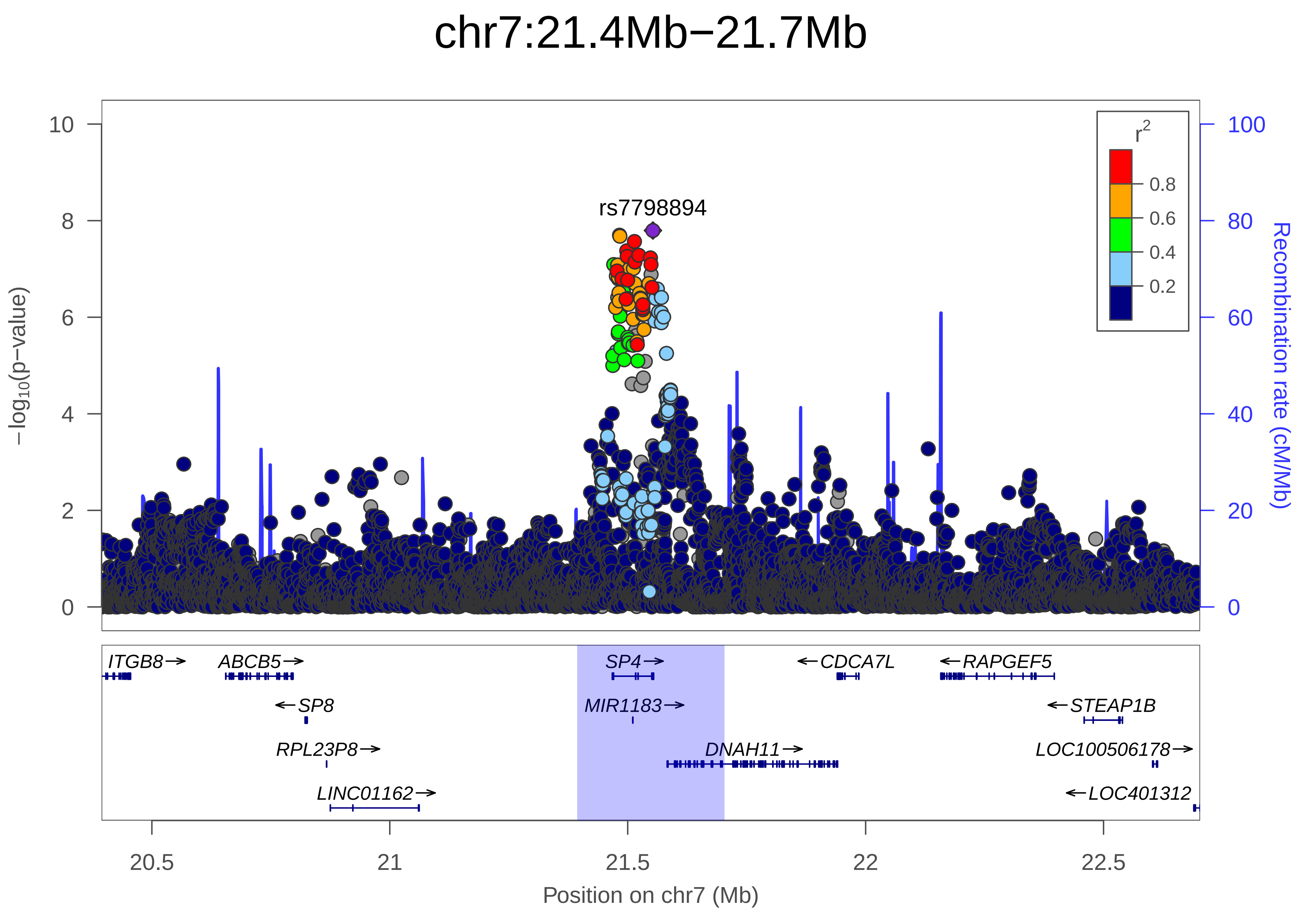

chr7:21.4Mb−21.7Mb
rs7798894
r2
0.8
0.6
0.4
0.2
10
8
6
4
2
0
−log10(p−value)
100
80
60
40
20
0
Recombination rate (cM/Mb)
ITGB8→
ABCB5→
←SP8
RPL23P8→
LINC01162→
SP4→
MIR1183→
DNAH11→
←CDCA7L
←RAPGEF5
←STEAP1B
LOC100506178→
←LOC401312
20.5
21
21.5
22
22.5
Position on chr7 (Mb)

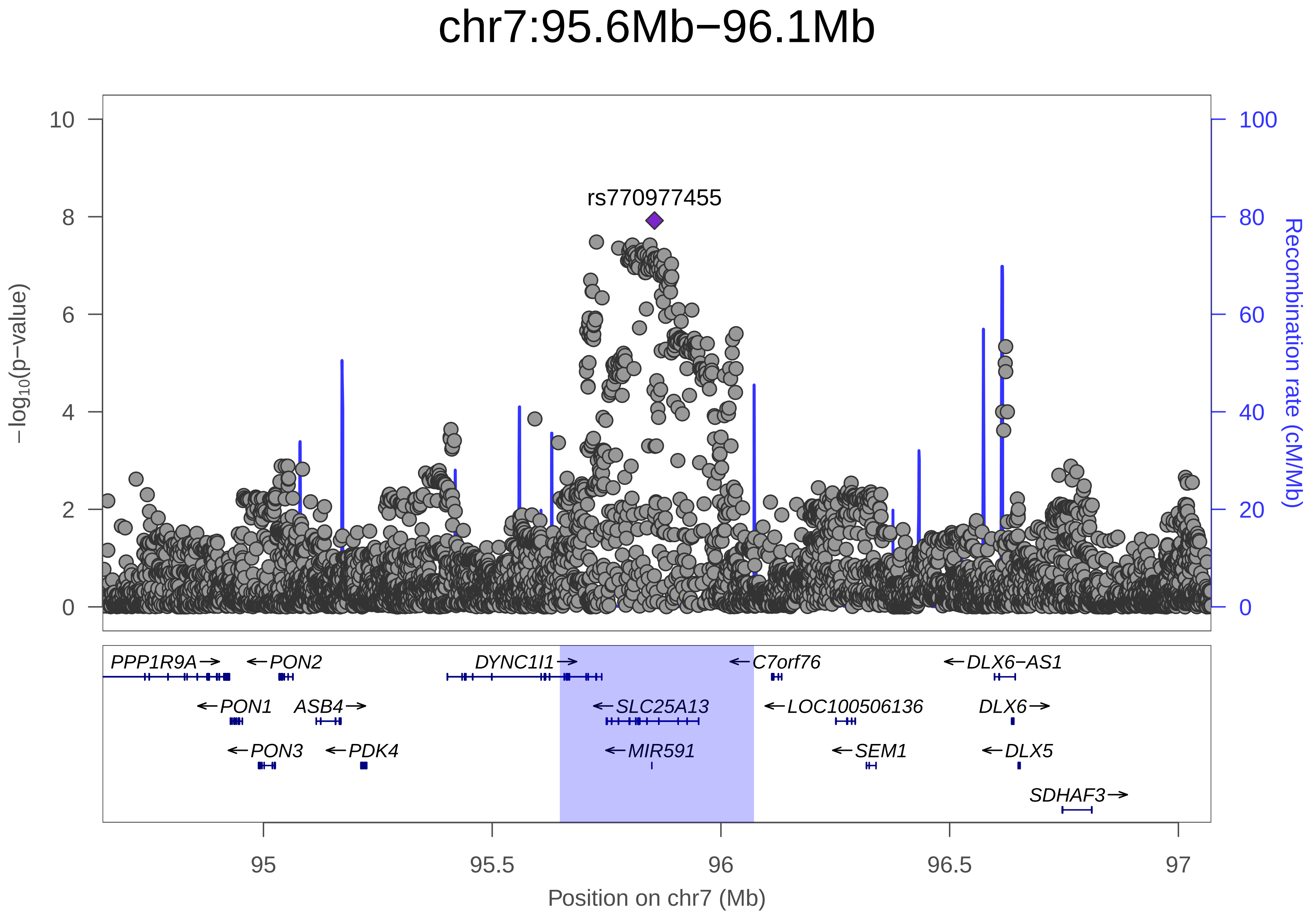

chr7:95.6Mb−96.1Mb
rs770977455
−log10(p−value)
Recombination rate (cM/Mb)
0
2
4
6
8
10
0
20
40
60
80
100
PPP1R9A→
←PON2
DYNC1I1→
←C7orf76
←DLX6−AS1
←PON1
ASB4→
←SLC25A13
←LOC100506136
DLX6→
←PON3
←PDK4
←MIR591
←SEM1
←DLX5
SDHAF3→
95
95.5
96
96.5
97
Position on chr7 (Mb)

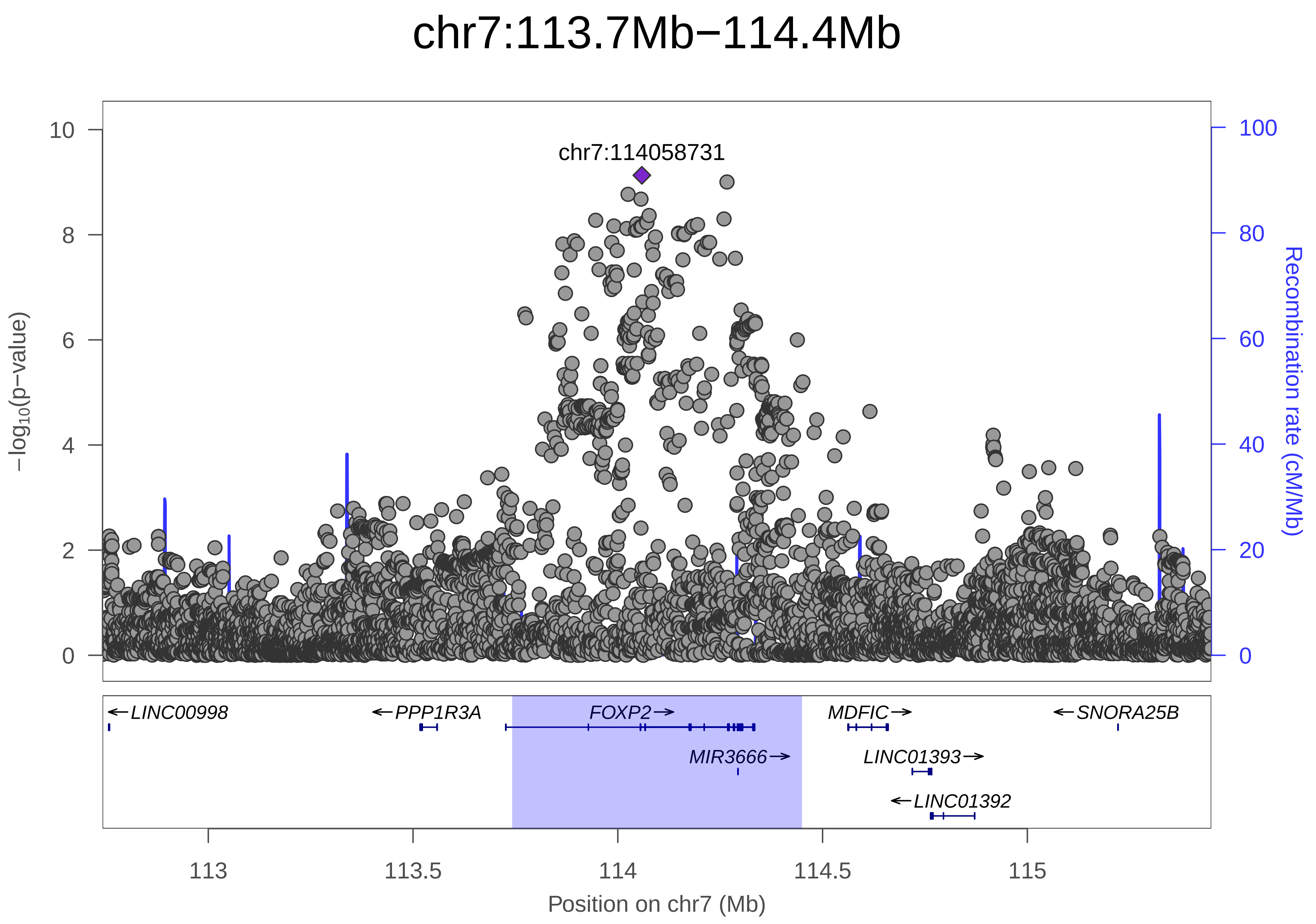
chr7:113.7Mb−114.4Mb
chr7:114058731
−log10(p−value)
Recombination rate (cM/Mb)
10
8
6
4
2
0
100
80
60
40
20
0
LINC00998
PPP1R3A
FOXP2
MIR3666
MDFIC
LINC01393
LINC01392
SNORA25B
113
113.5
114
114.5
115
Position on chr7 (Mb)

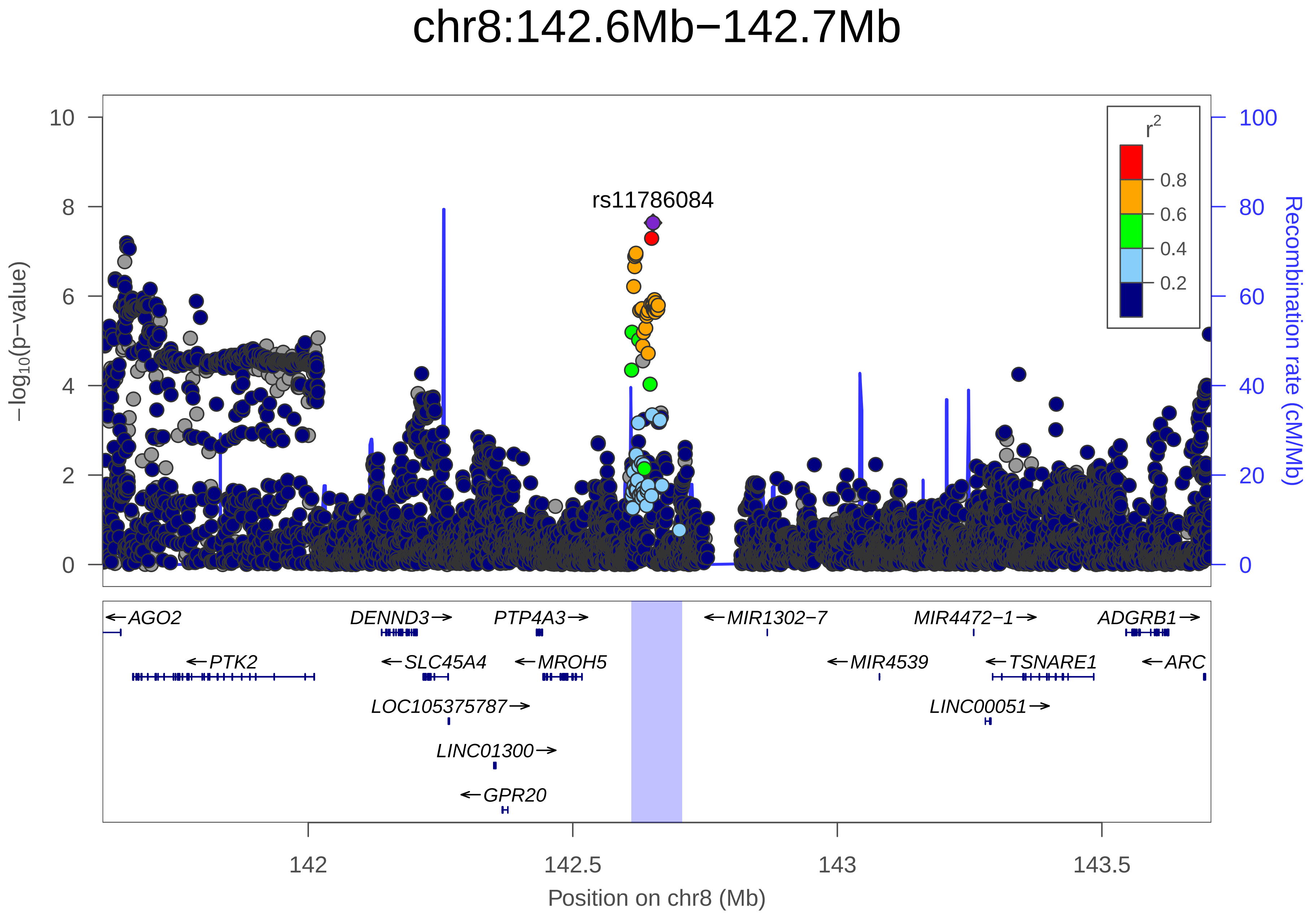
chr8:142.6Mb−142.7Mb
rs11786084
r2
0.8
0.6
0.4
0.2
−log10(p−value)
Recombination rate (cM/Mb)
0
2
4
6
8
10
0
20
40
60
80
100
←AGO2
←PTK2
DENND3→
←SLC45A4
LOC105375787→
LINC01300→
←GPR20
PTP4A3→
←MROH5
←MIR1302−7
←MIR4539
MIR4472−1→
←TSNARE1
LINC00051→
ADGRB1→
←ARC
142
142.5
143
143.5
Position on chr8 (Mb)

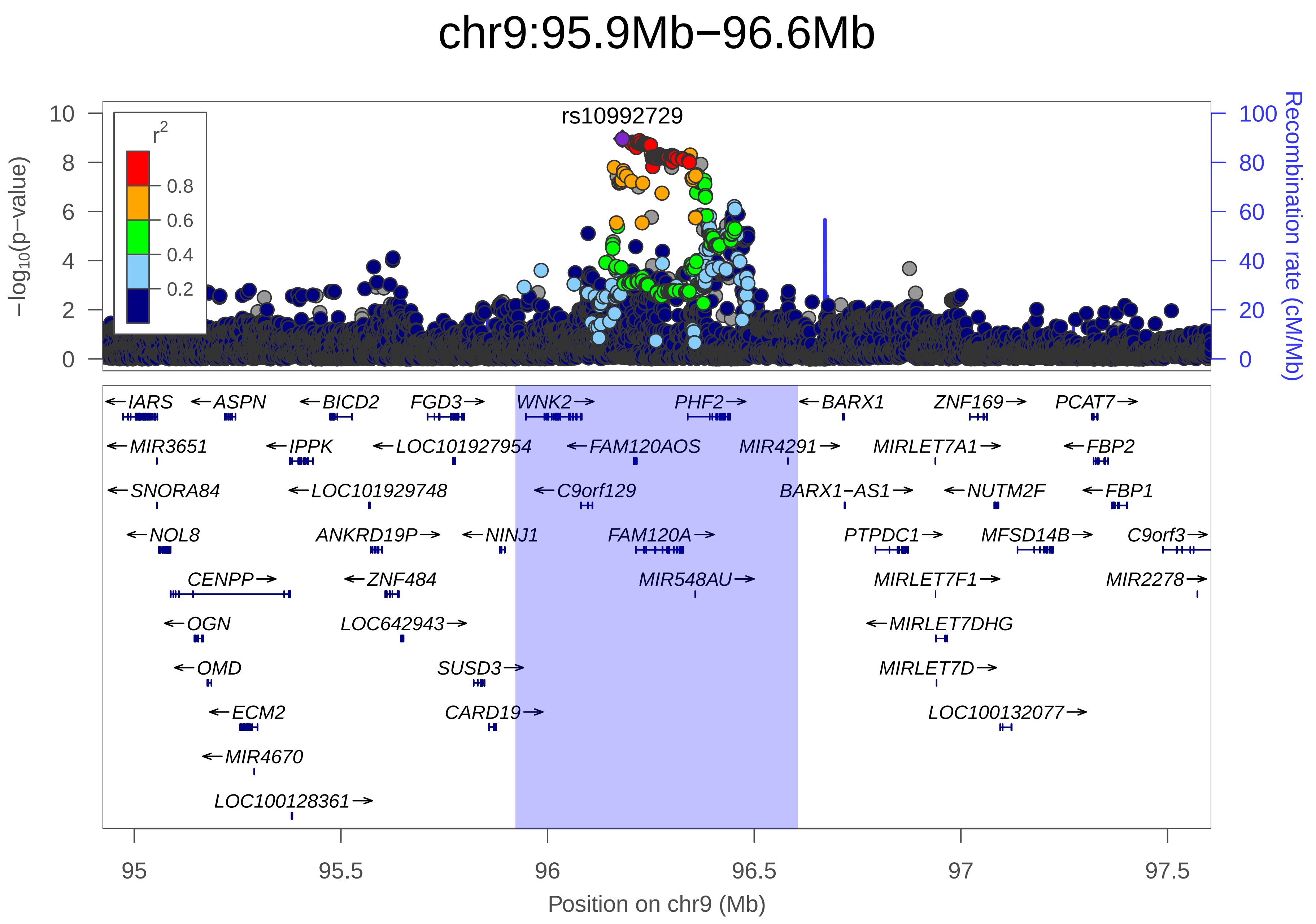
chr9:95.9Mb−96.6Mb
rs10992729
r2
0.8
0.6
0.4
0.2
10
8
6
4
2
0
−log10(p−value)
100
80
60
40
20
0
Recombination rate (cM/Mb)
←IARS
←ASPN
←BICD2
FGD3→
WNK2→
PHF2→
←BARX1
ZNF169→
PCAT7→
←MIR3651
←IPPK
←LOC101927954
←FAM120AOS
MIR4291→
MIRLET7A1→
←FBP2
←SNORA84
←LOC101929748
←C9orf129
BARX1−AS1→
←NUTM2F
←FBP1
←NOL8
ANKRD19P→
←NINJ1
FAM120A→
PTPDC1→
MFSD14B→
C9orf3→
CENPP→
←ZNF484
MIR548AU→
MIRLET7F1→
MIR2278→
←OGN
LOC642943→
←MIRLET7DHG
←OMD
SUSD3→
MIRLET7D→
←ECM2
CARD19→
LOC100132077→
←MIR4670
LOC100128361→
95
95.5
96
96.5
97
97.5
Position on chr9 (Mb)

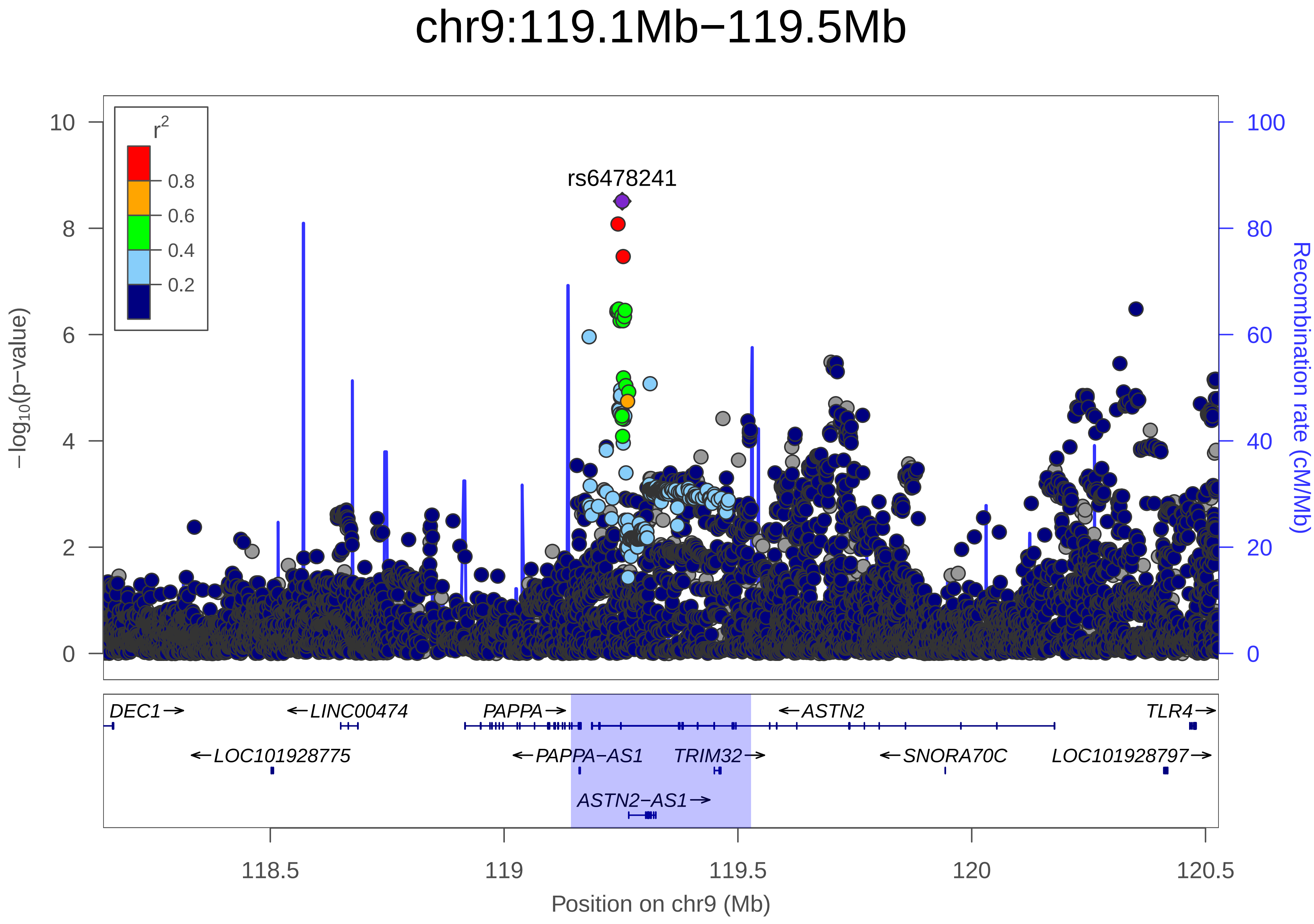
chr9:119.1Mb−119.5Mb
rs6478241
r2
0.8
0.6
0.4
0.2
−log10(p−value)
Recombination rate (cM/Mb)
0
2
4
6
8
10
0
20
40
60
80
100
DEC1→
←LINC00474
PAPPA→
←ASTN2
TLR4→
←LOC101928775
←PAPPA−AS1
TRIM32→
←SNORA70C
LOC101928797→
ASTN2−AS1→
118.5
119
119.5
120
120.5
Position on chr9 (Mb)

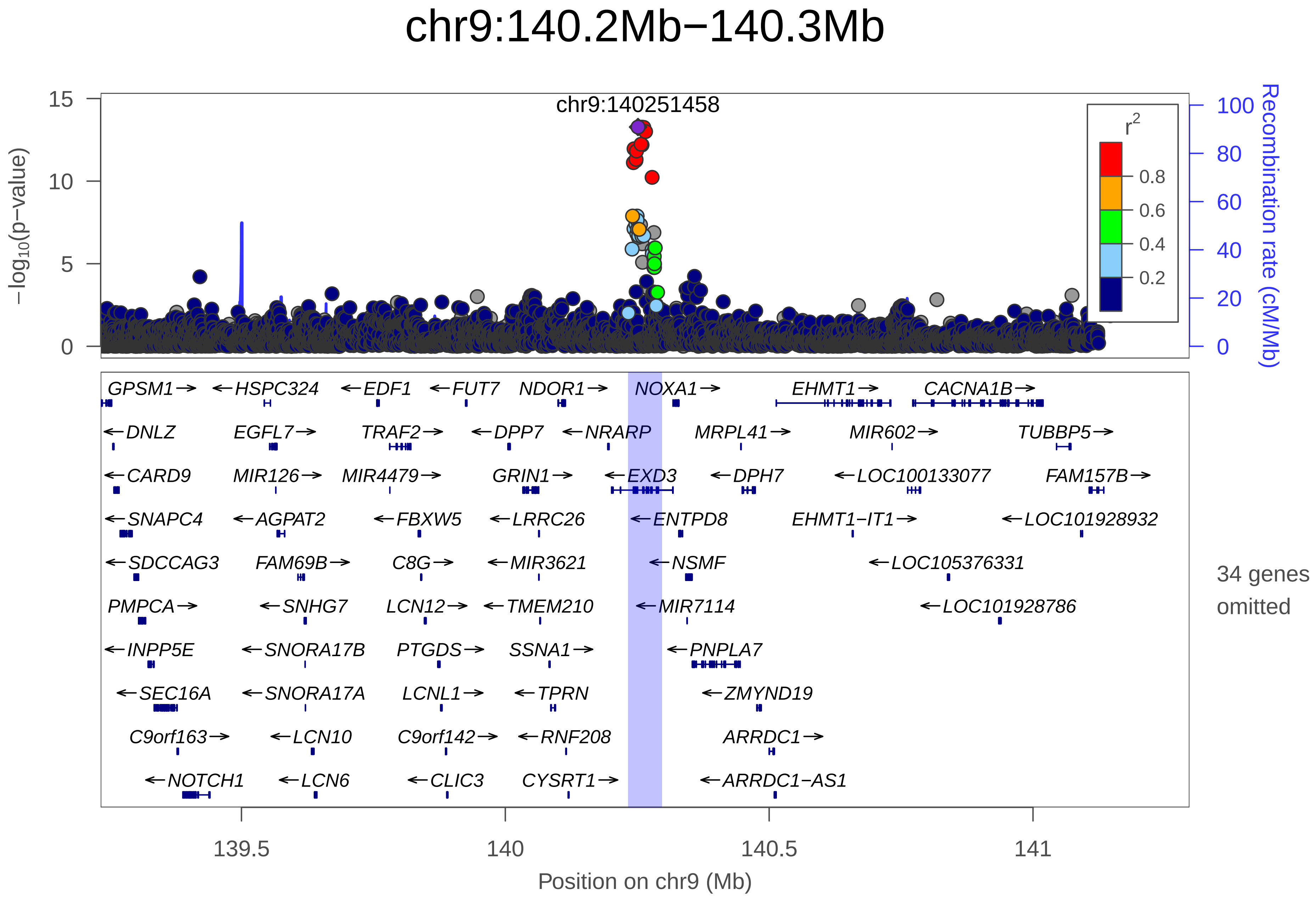

chr9:140.2Mb−140.3Mb
chr9:140251458
−log10(p−value)
Recombination rate (cM/Mb)
r2
0.8
0.6
0.4
0.2
15
10
5
0
100
80
60
40
20
0
GPSM1→
←HSPC324
←EDF1
←FUT7
NDOR1→
NOXA1→
EHMT1→
CACNA1B→
←DNLZ
EGFL7→
TRAF2→
←DPP7
←NRARP
MRPL41→
MIR602→
TUBBP5→
←CARD9
MIR126→
MIR4479→
GRIN1→
←EXD3
←DPH7
←LOC100133077
FAM157B→
←SNAPC4
←AGPAT2
←FBXW5
←LRRC26
←ENTPD8
EHMT1−IT1→
←LOC101928932
←SDCCAG3
FAM69B→
C8G→
←MIR3621
←NSMF
←LOC105376331
PMPCA→
←SNHG7
LCN12→
←TMEM210
←MIR7114
←LOC101928786
←INPP5E
←SNORA17B
PTGDS→
SSNA1→
←PNPLA7
←SEC16A
←SNORA17A
LCNL1→
←TPRN
←ZMYND19
C9orf163→
←LCN10
C9orf142→
←RNF208
ARRDC1→
←NOTCH1
←LCN6
←CLIC3
CYSRT1→
←ARRDC1−AS1
34 genes omitted
139.5
140
140.5
141
Position on chr9 (Mb)

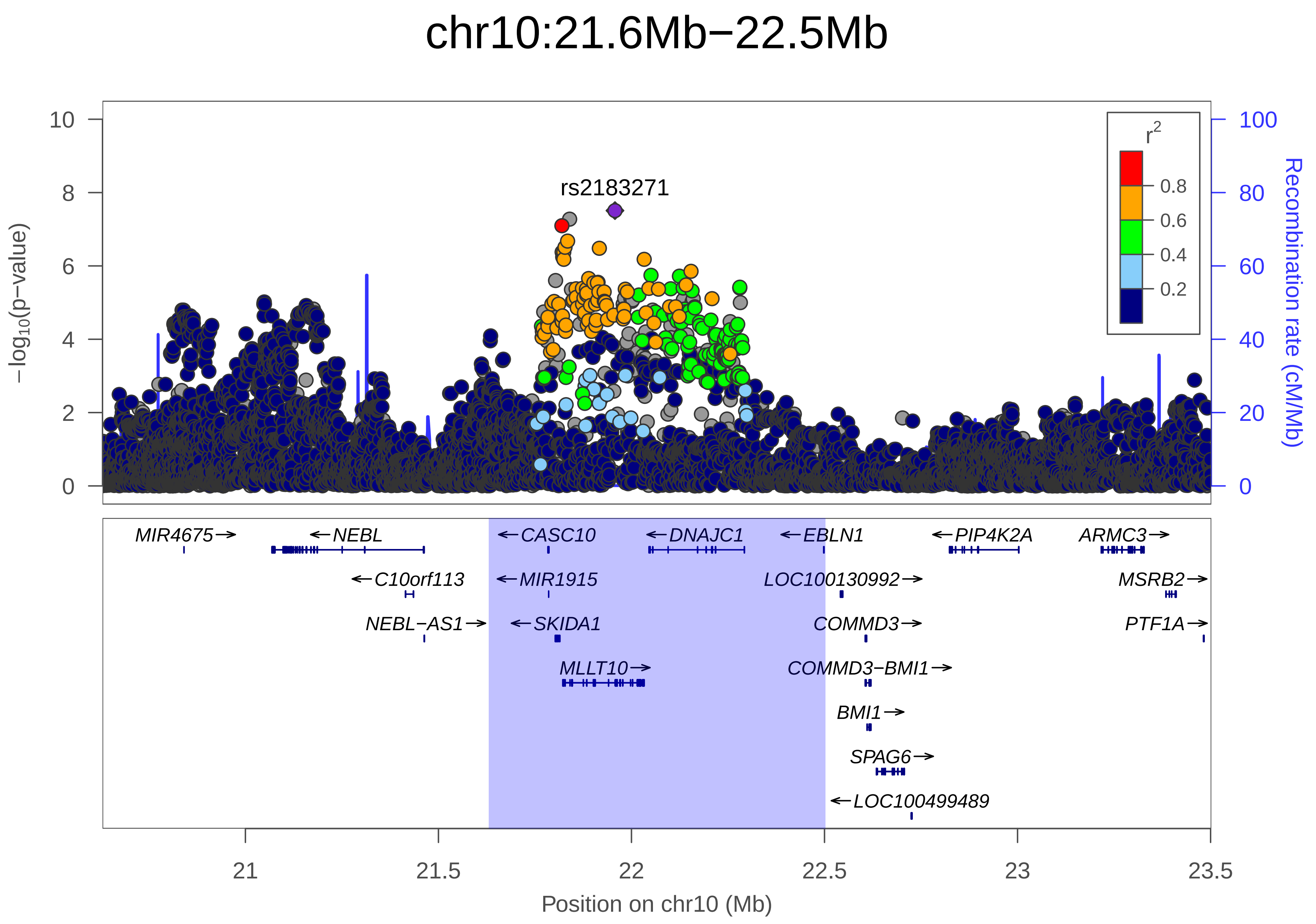
chr10:21.6Mb−22.5Mb
rs2183271
$-\log_{10}$(p−value)
Recombination rate (cM/Mb)
r^2
0.8
0.6
0.4
0.2
MIR4675→
←NEBL
←CASC10
←DNAJC1
←EBLN1
←PIP4K2A
ARMC3→
←C10orf113
←MIR1915
LOC100130992→
MSRB2→
NEBL−AS1→
←SKIDA1
COMMD3→
PTF1A→
MLLT10→
COMMD3−BMI1→
BMI1→
SPAG6→
←LOC100499489
Position on chr10 (Mb)

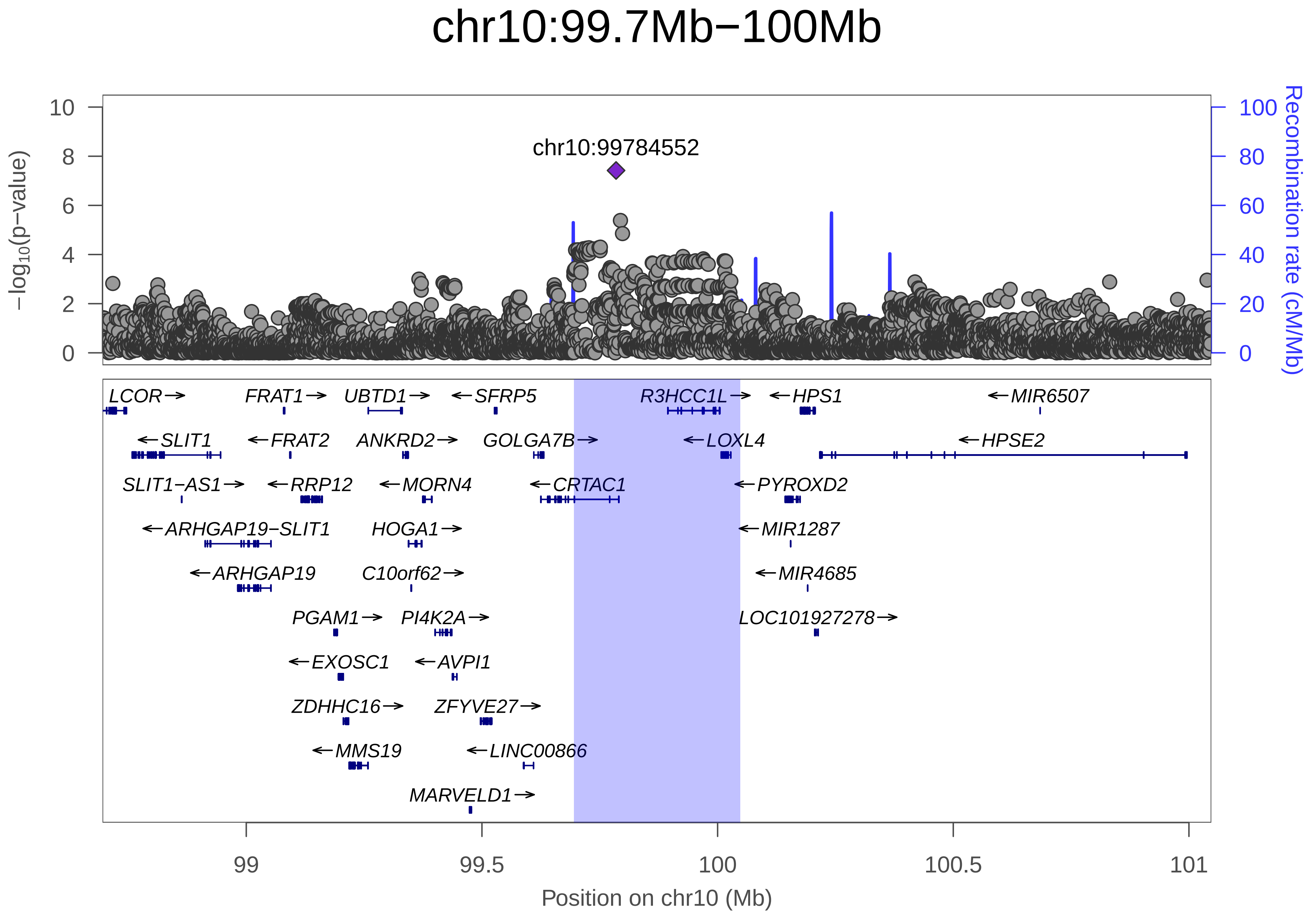

chr10:99.7Mb−100Mb
chr10:99784552
−log10(p−value)
Recombination rate (cM/Mb)
0
2
4
6
8
10
0
20
40
60
80
100
LCOR→
FRAT1→
UBTD1→
←SFRP5
R3HCC1L→
←HPS1
←MIR6507
←SLIT1
←FRAT2
ANKRD2→
GOLGA7B→
←LOXL4
←HPSE2
SLIT1−AS1→
←RRP12
←MORN4
←CRTAC1
←PYROXD2
←ARHGAP19−SLIT1
HOGA1→
←MIR1287
←ARHGAP19
C10orf62→
←MIR4685
PGAM1→
PI4K2A→
LOC101927278→
←EXOSC1
←AVPI1
ZDHHC16→
ZFYVE27→
←MMS19
←LINC00866
MARVELD1→
99
99.5
100
100.5
101
Position on chr10 (Mb)

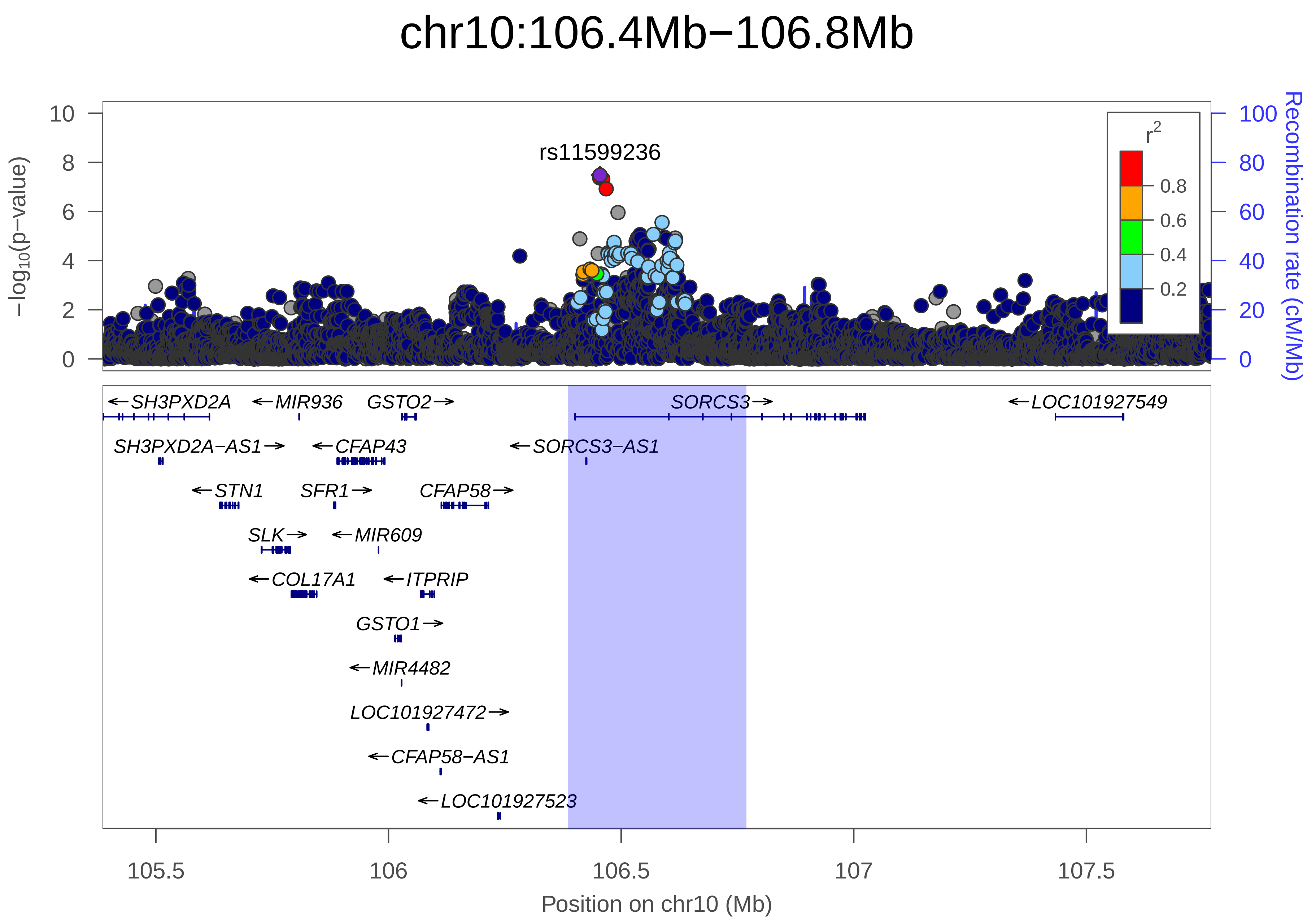

chr10:106.4Mb−106.8Mb
rs11599236
−log10(p−value)
Recombination rate (cM/Mb)
r2
0.8
0.6
0.4
0.2
0
2
4
6
8
10
0
20
40
60
80
100
SH3PXD2A
MIR936
GSTO2
SORCS3
LOC101927549
SH3PXD2A−AS1
CFAP43
SORCS3−AS1
STN1
SFR1
CFAP58
SLK
MIR609
COL17A1
ITPRIP
GSTO1
MIR4482
LOC101927472
CFAP58−AS1
LOC101927523
105.5
106
106.5
107
107.5
Position on chr10 (Mb)

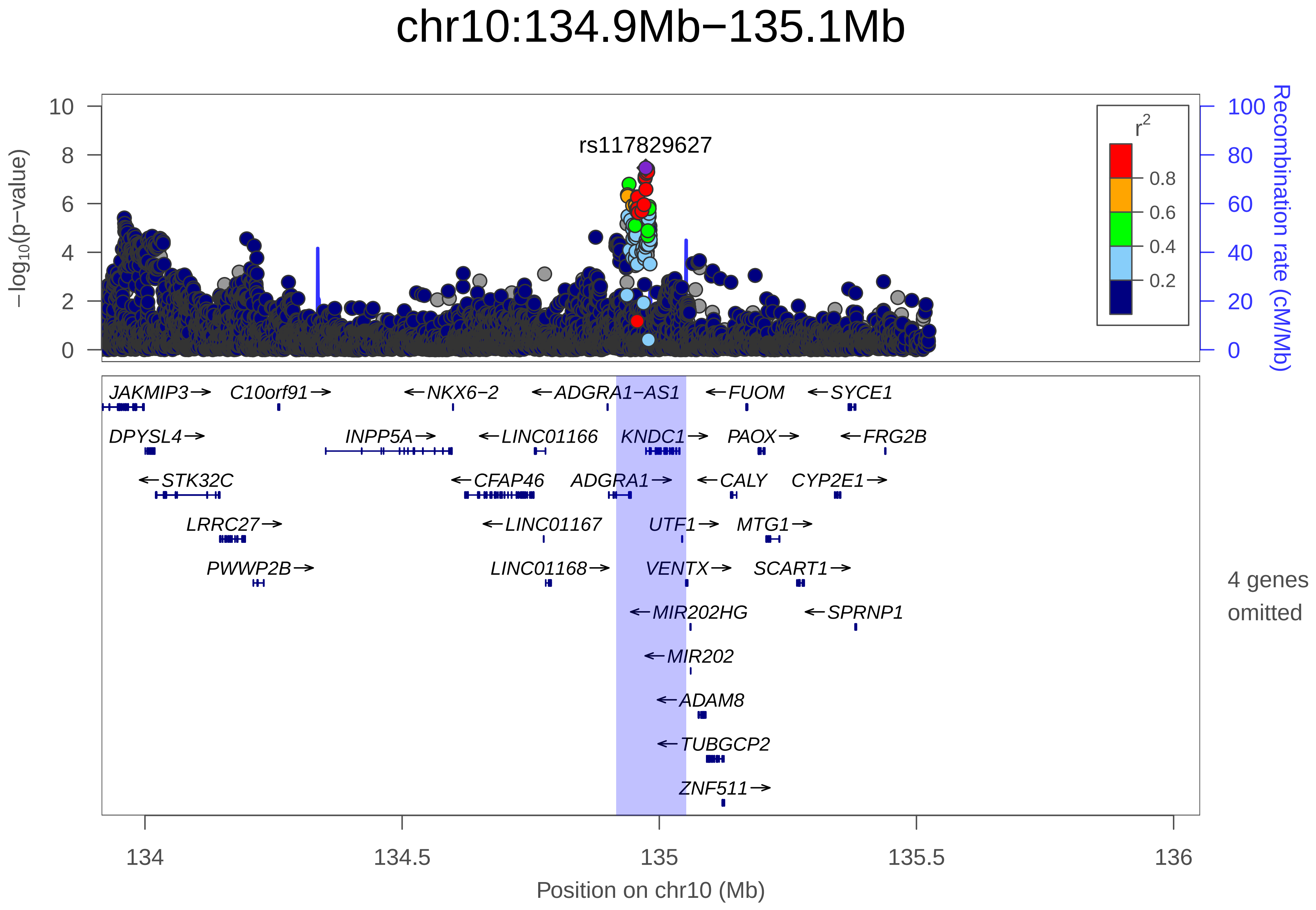

chr10:134.9Mb−135.1Mb
rs117829627
−log10(p−value)
Recombination rate (cM/Mb)
r2
0.8
0.6
0.4
0.2
JAKMIP3
C10orf91
NKX6−2
ADGRA1−AS1
FUOM
SYCE1
DPYSL4
INPP5A
LINC01166
KNDC1
PAOX
FRG2B
STK32C
CFAP46
ADGRA1
CALY
CYP2E1
LRRC27
LINC01167
UTF1
MTG1
PWWP2B
LINC01168
VENTX
SCART1
MIR202HG
SPRNP1
MIR202
ADAM8
TUBGCP2
ZNF511
4 genes omitted
134
134.5
135
135.5
136
Position on chr10 (Mb)

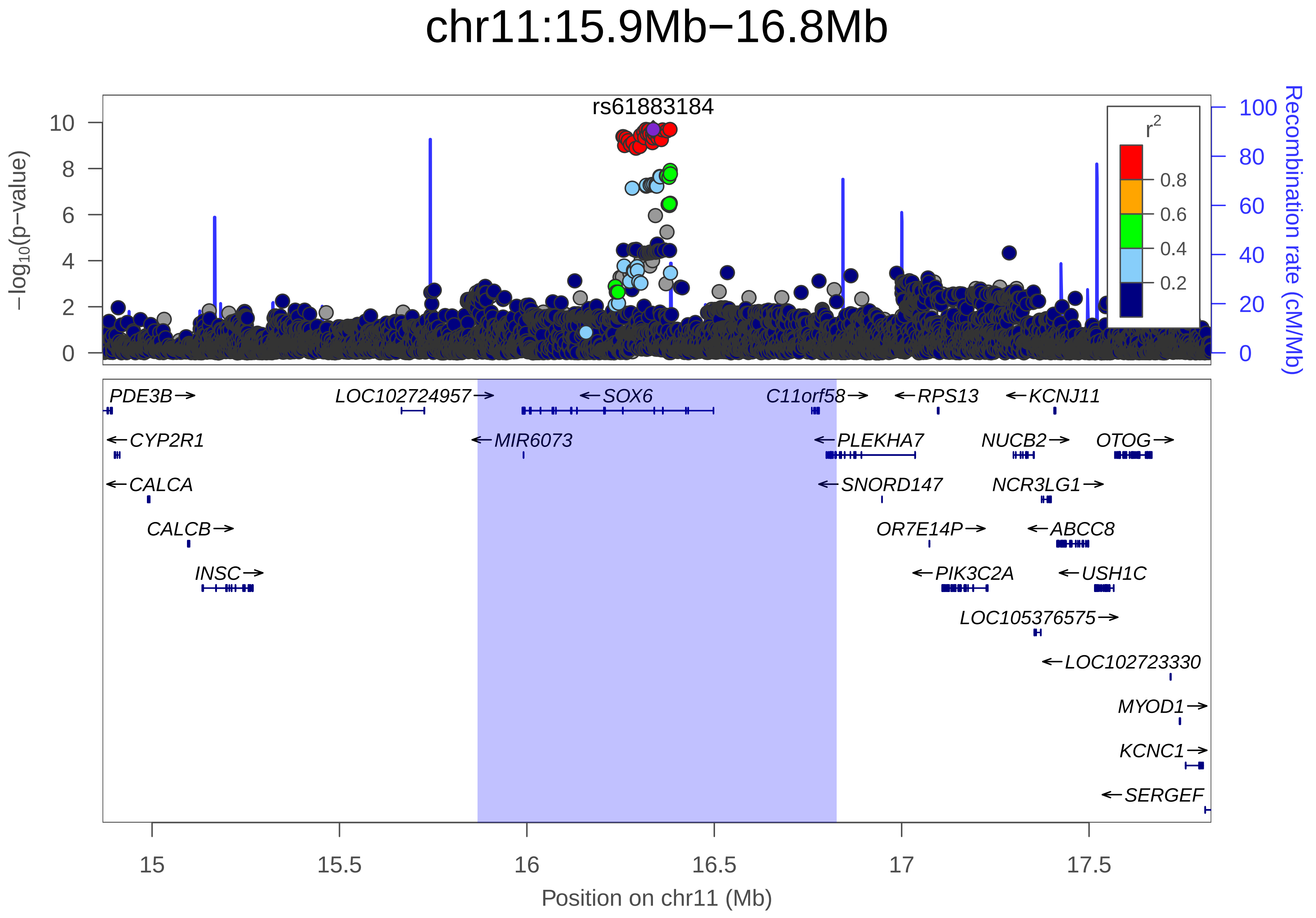

chr11:15.9Mb−16.8Mb
rs61883184
−log10(p−value)
Recombination rate (cM/Mb)
r2
0.8
0.6
0.4
0.2
PDE3B
LOC102724957
SOX6
C11orf58
RPS13
KCNJ11
CYP2R1
MIR6073
PLEKHA7
NUCB2
OTOG
CALCA
SNORD147
NCR3LG1
CALCB
OR7E14P
ABCC8
INSC
PIK3C2A
USH1C
LOC105376575
LOC102723330
MYOD1
KCNC1
SERGEF
Position on chr11 (Mb)

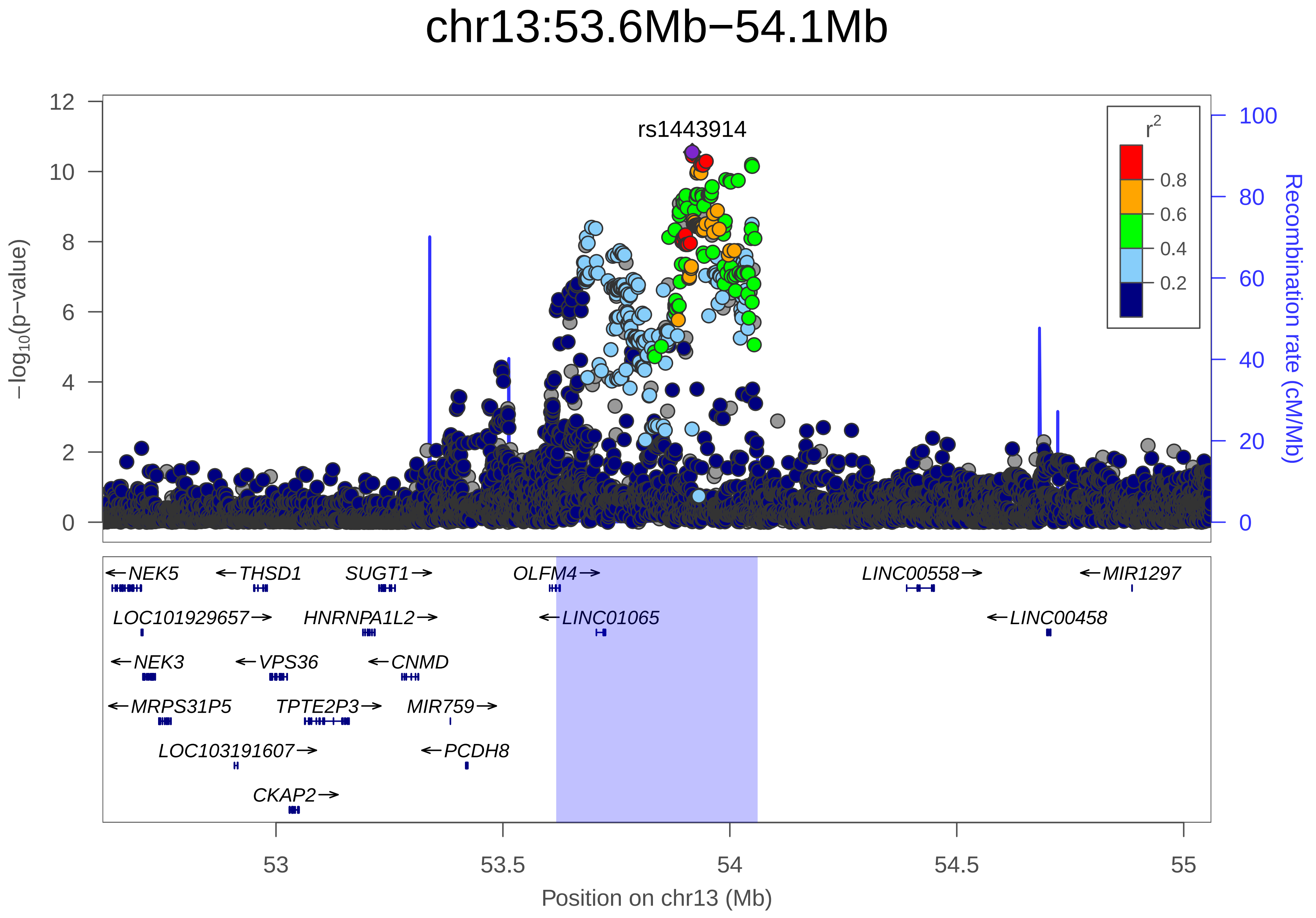
chr13:53.6Mb−54.1Mb
rs1443914
r²
0.8
0.6
0.4
0.2
−log10(p−value)
Recombination rate (cM/Mb)
NEK5
THSD1
SUGT1
OLFM4
LINC00558
MIR1297
LOC101929657
HNRNPA1L2
LINC01065
LINC00458
NEK3
VPS36
CNMD
MRPS31P5
TPTE2P3
MIR759
LOC103191607
PCDH8
CKAP2
Position on chr13 (Mb)

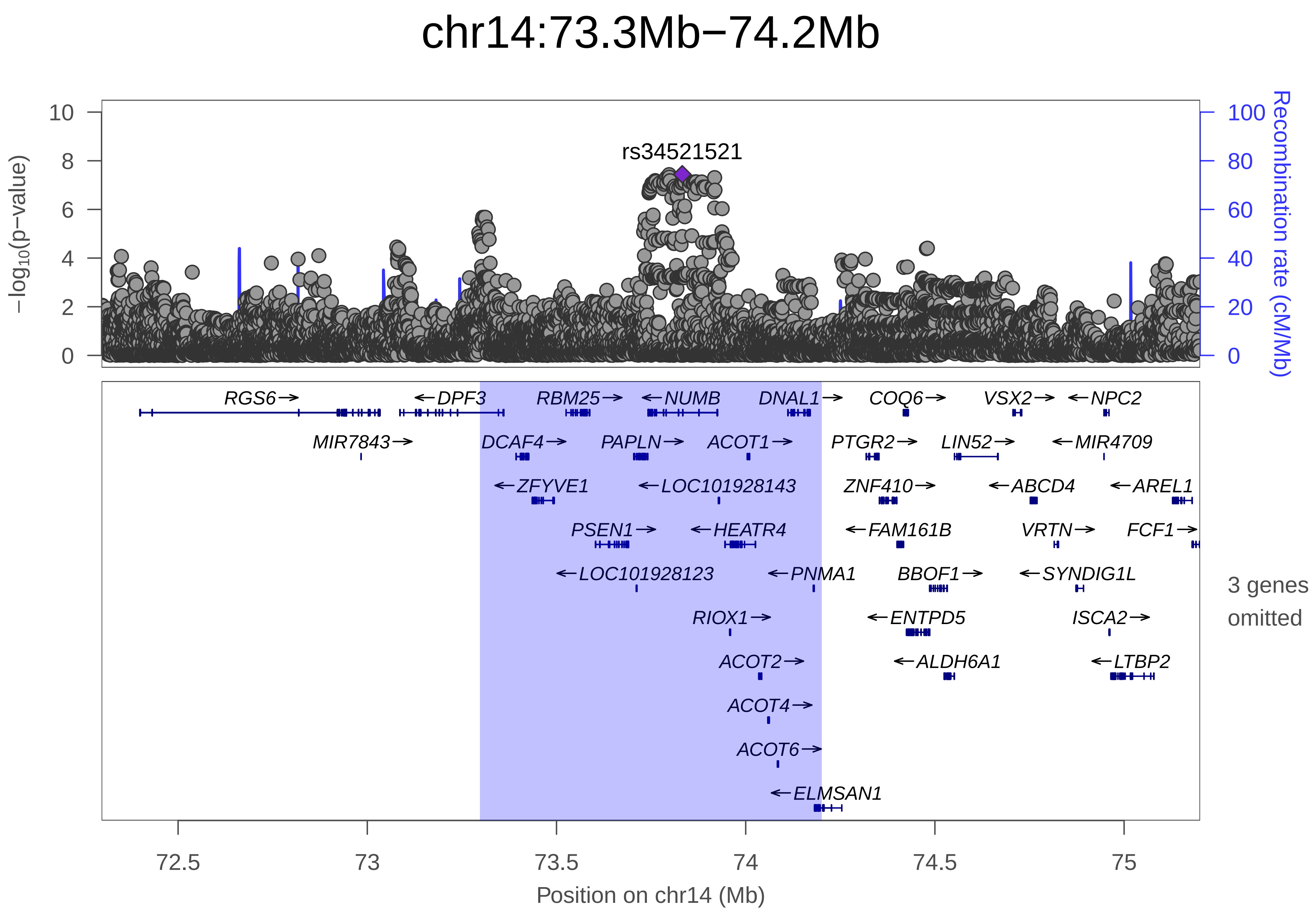

chr14:73.3Mb−74.2Mb
rs34521521
−log10(p−value)
Recombination rate (cM/Mb)
0
2
4
6
8
10
0
20
40
60
80
100
RGS6→
←DPF3
RBM25→
←NUMB
DNAL1→
COQ6→
VSX2→
←NPC2
MIR7843→
DCAF4→
PAPLN→
ACOT1→
PTGR2→
LIN52→
←MIR4709
←ZFYVE1
←LOC101928143
ZNF410→
←ABCD4
←AREL1
PSEN1→
←HEATR4
←FAM161B
VRTN→
FCF1→
←LOC101928123
←PNMA1
BBOF1→
←SYNDIG1L
RIOX1→
←ENTPD5
ISCA2→
ACOT2→
←ALDH6A1
←LTBP2
ACOT4→
ACOT6→
←ELMSAN1
3 genes omitted
72.5
73
73.5
74
74.5
75
Position on chr14 (Mb)

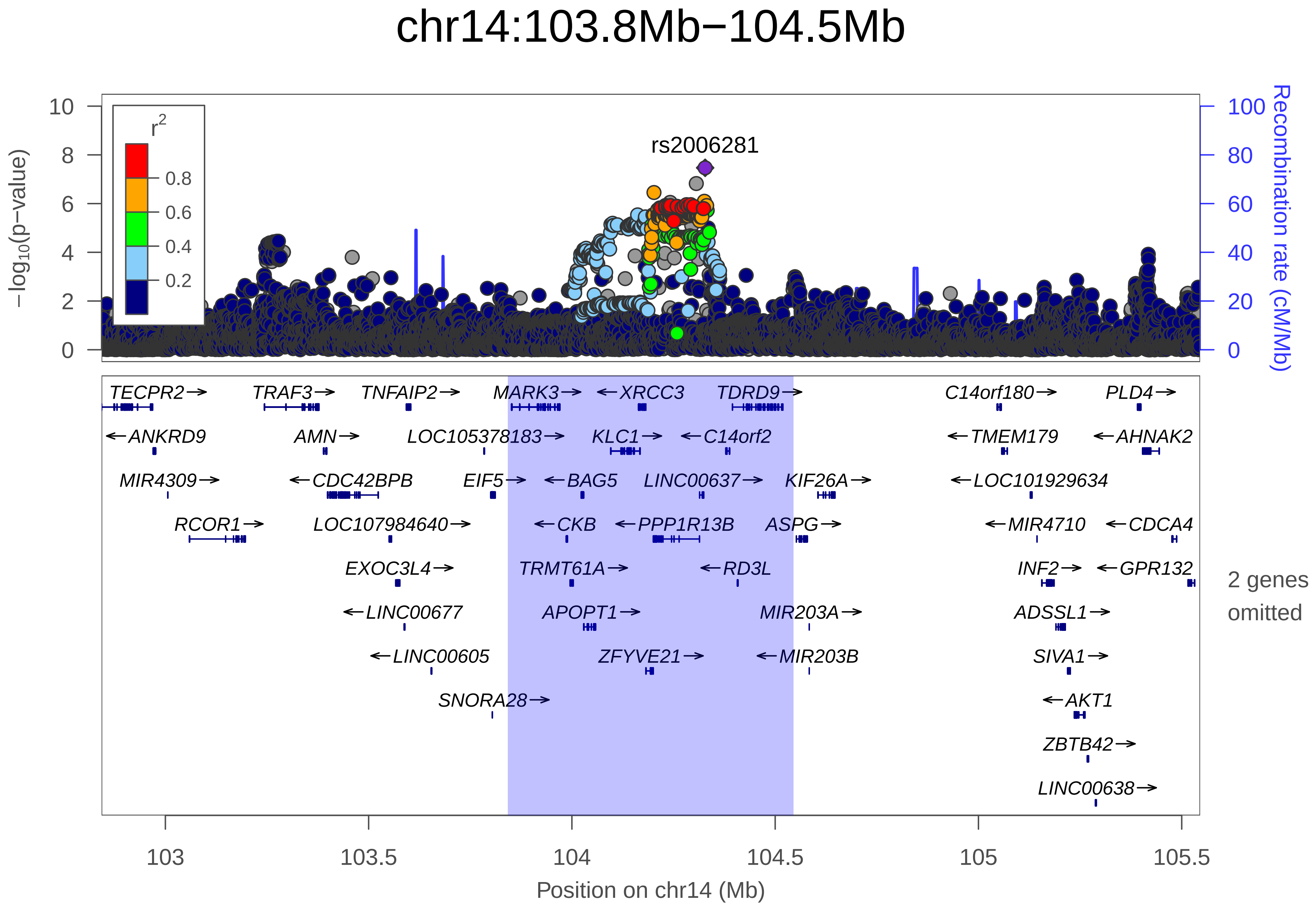

chr14:103.8Mb−104.5Mb
rs2006281
r2
0.8
0.6
0.4
0.2
−log10(p−value)
Recombination rate (cM/Mb)
0
2
4
6
8
10
20
40
60
80
100
TECPR2→
TRAF3→
TNFAIP2→
MARK3→
←XRCC3
TDRD9→
C14orf180→
PLD4→
←ANKRD9
AMN→
LOC105378183→
KLC1→
←C14orf2
←TMEM179
←AHNAK2
MIR4309→
←CDC42BPB
EIF5→
←BAG5
LINC00637→
KIF26A→
←LOC101929634
RCOR1→
LOC107984640→
←CKB
←PPP1R13B
ASPG→
←MIR4710
←CDCA4
EXOC3L4→
TRMT61A→
←RD3L
INF2→
←GPR132
←LINC00677
APOPT1→
MIR203A→
ADSSL1→
←LINC00605
ZFYVE21→
←MIR203B
SIVA1→
SNORA28→
←AKT1
ZBTB42→
LINC00638→
2 genes omitted
103
103.5
104
104.5
105
105.5
Position on chr14 (Mb)

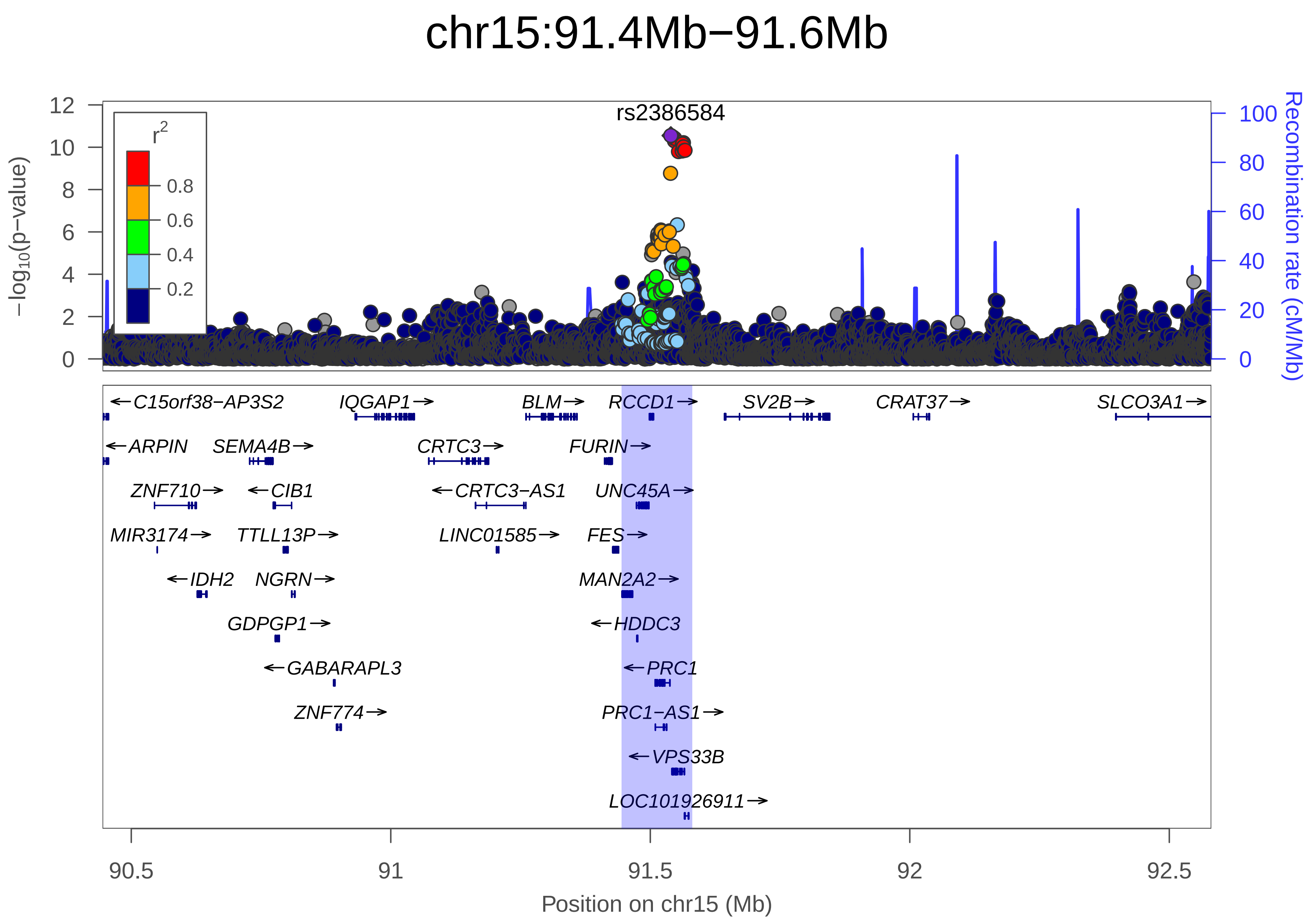

chr15:91.4Mb−91.6Mb
rs2386584
r2
0.8
0.6
0.4
0.2
−log10(p−value)
Recombination rate (cM/Mb)
←C15orf38−AP3S2
IQGAP1→
BLM→
RCCD1→
SV2B→
CRAT37→
SLCO3A1→
←ARPIN
SEMA4B→
CRTC3→
FURIN→
ZNF710→
←CIB1
←CRTC3−AS1
UNC45A→
MIR3174→
TTLL13P→
LINC01585→
FES→
←IDH2
NGRN→
MAN2A2→
GDPGP1→
←HDDC3
←GABARAPL3
←PRC1
ZNF774→
PRC1−AS1→
←VPS33B
LOC101926911→
Position on chr15 (Mb)

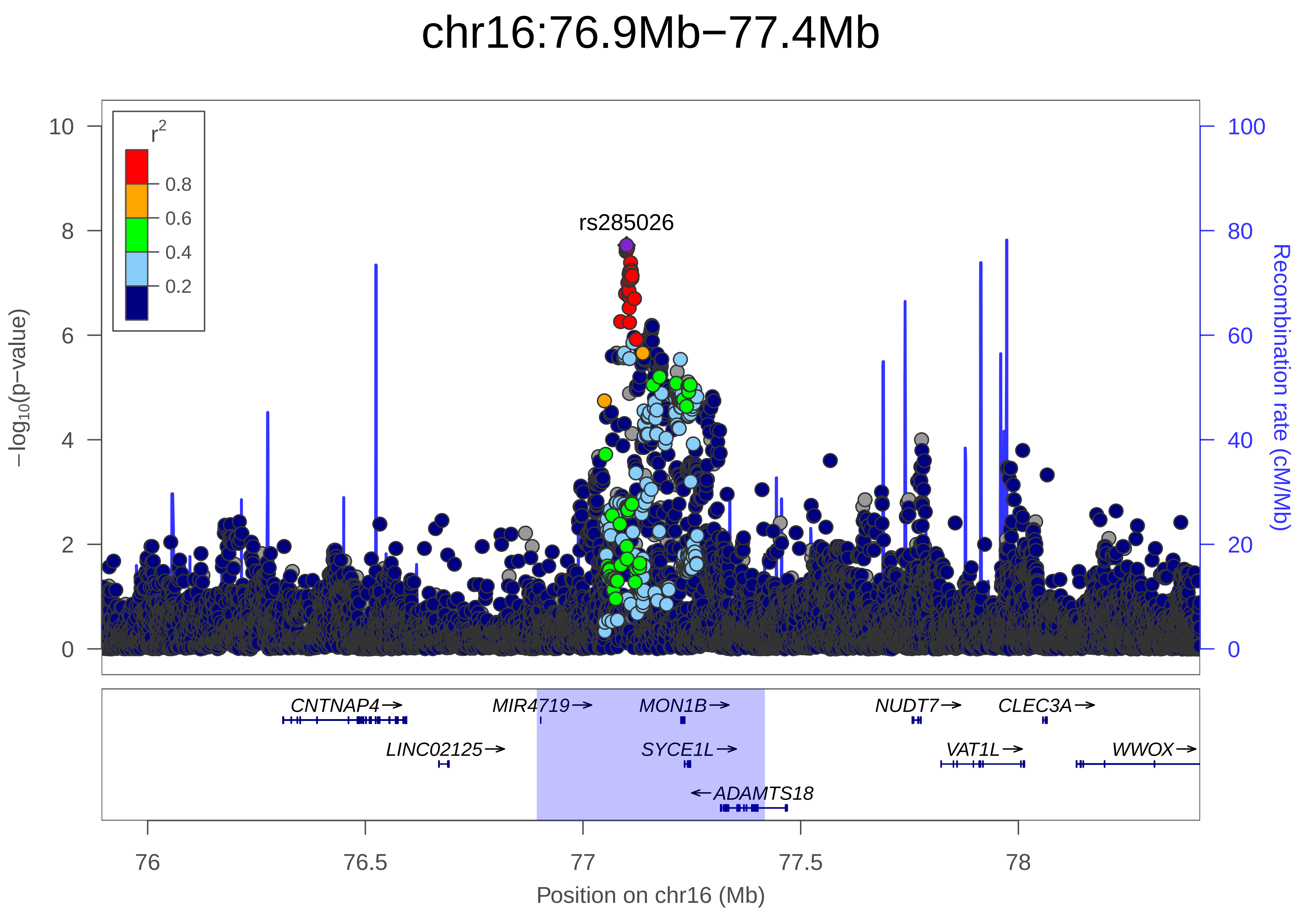

chr16:76.9Mb−77.4Mb
r2
0.8
0.6
0.4
0.2
rs285026
−log10(p−value)
Recombination rate (cM/Mb)
CNTNAP4→
MIR4719→
MON1B→
NUDT7→
CLEC3A→
LINC02125→
SYCE1L→
VAT1L→
WWOX→
←ADAMTS18
Position on chr16 (Mb)

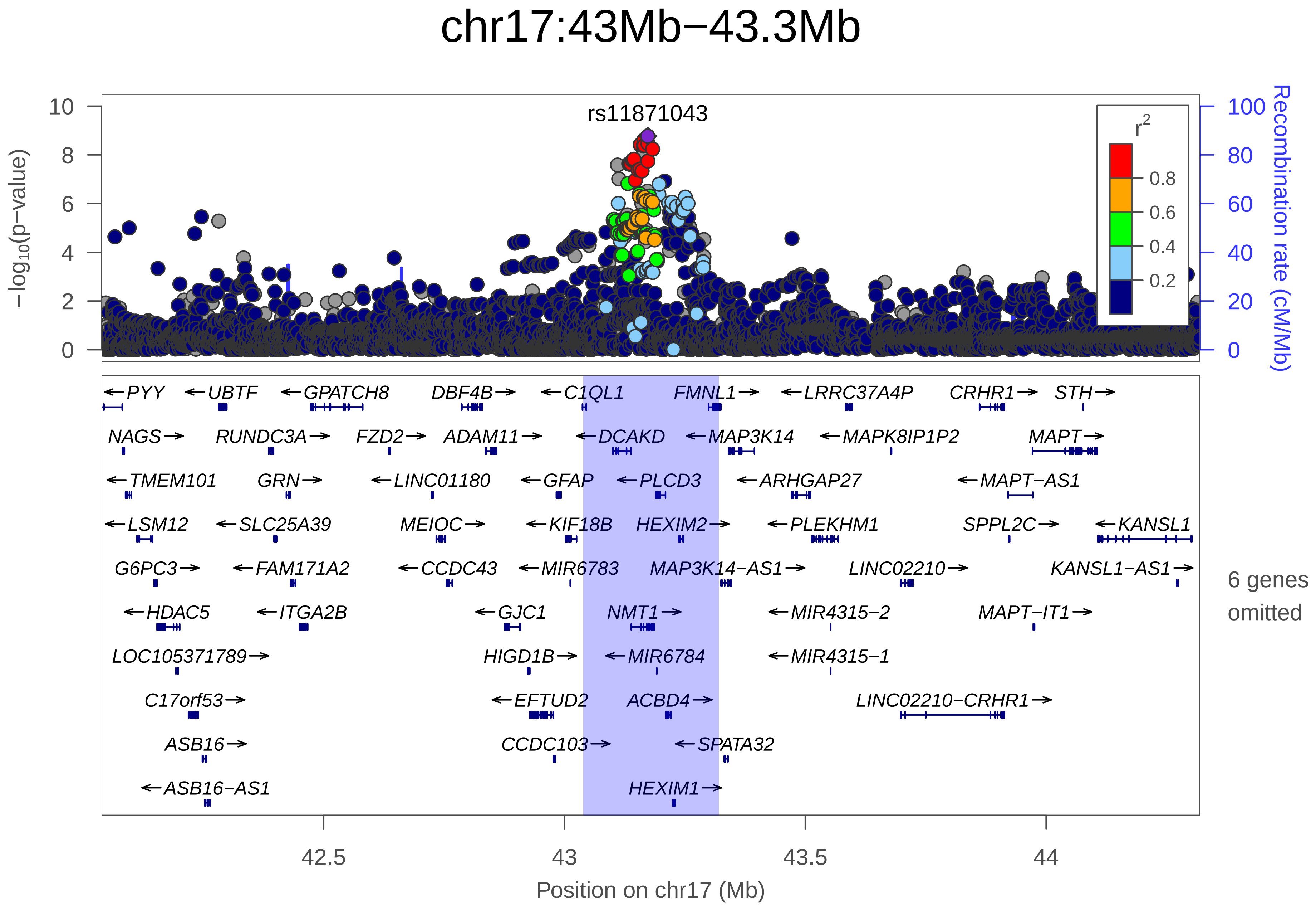
chr17:43Mb−43.3Mb
rs11871043
−log10(p−value)
Recombination rate (cM/Mb)
r2
0.8
0.6
0.4
0.2
←PYY
←UBTF
←GPATCH8
DBF4B→
←C1QL1
FMNL1→
←LRRC37A4P
CRHR1→
STH→
NAGS→
RUNDC3A→
FZD2→
ADAM11→
←DCAKD
←MAP3K14
←MAPK8IP1P2
MAPT→
←TMEM101
GRN→
←LINC01180
←GFAP
←PLCD3
←ARHGAP27
←MAPT−AS1
←LSM12
←SLC25A39
MEIOC→
←KIF18B
HEXIM2→
←PLEKHM1
SPPL2C→
←KANSL1
G6PC3→
←FAM171A2
←CCDC43
←MIR6783
MAP3K14−AS1→
LINC02210→
KANSL1−AS1→
6 genes omitted
←HDAC5
←ITGA2B
←GJC1
NMT1→
←MIR4315−2
MAPT−IT1→
LOC105371789→
HIGD1B→
←MIR6784
←MIR4315−1
C17orf53→
←EFTUD2
ACBD4→
LINC02210−CRHR1→
ASB16→
CCDC103→
←SPATA32
←ASB16−AS1
HEXIM1→
Position on chr17 (Mb)

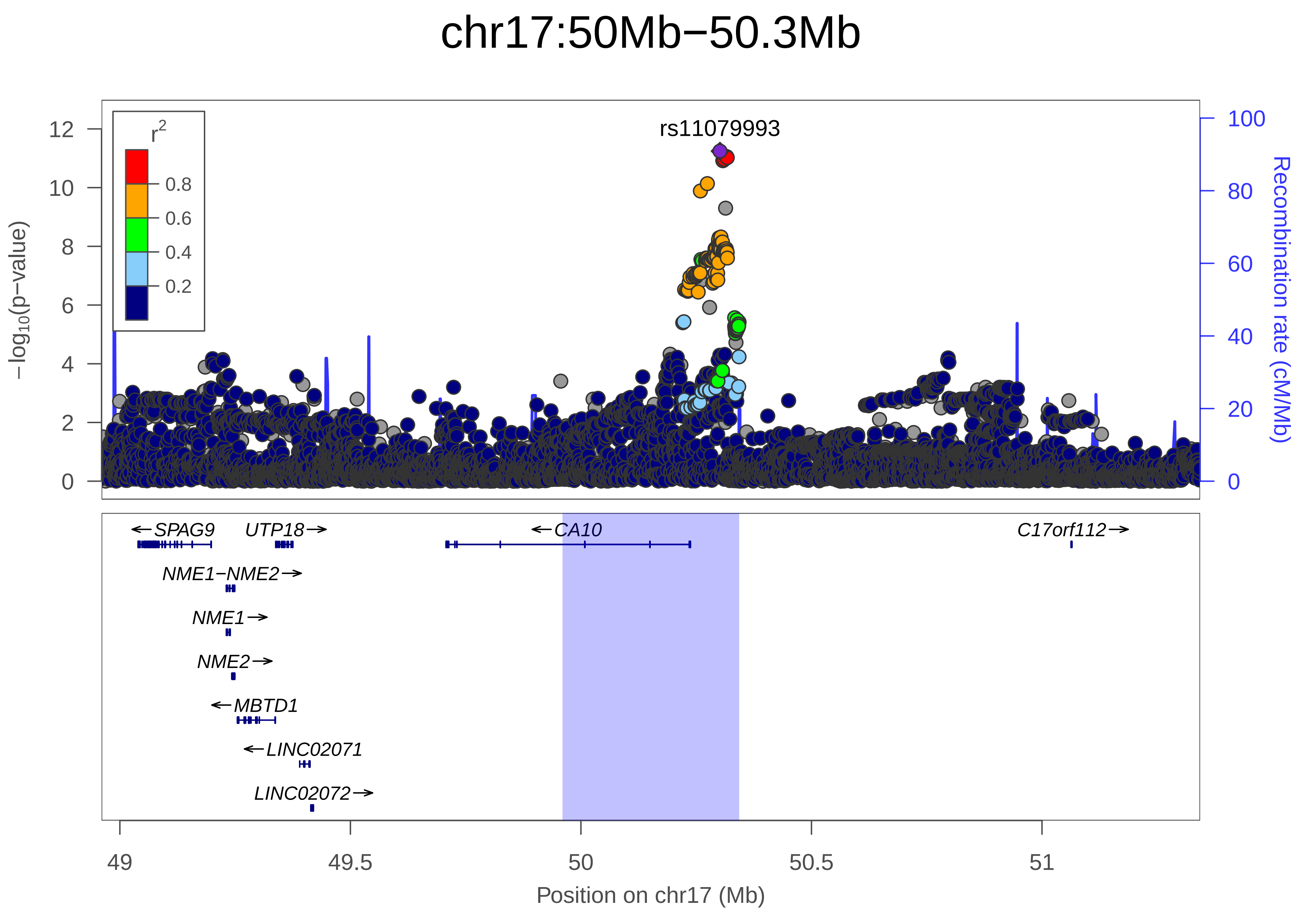
chr17:50Mb−50.3Mb
rs11079993
r2
0.8
0.6
0.4
0.2
−log10(p−value)
Recombination rate (cM/Mb)
SPAG9
UTP18
CA10
C17orf112
NME1−NME2
NME1
NME2
MBTD1
LINC02071
LINC02072
Position on chr17 (Mb)

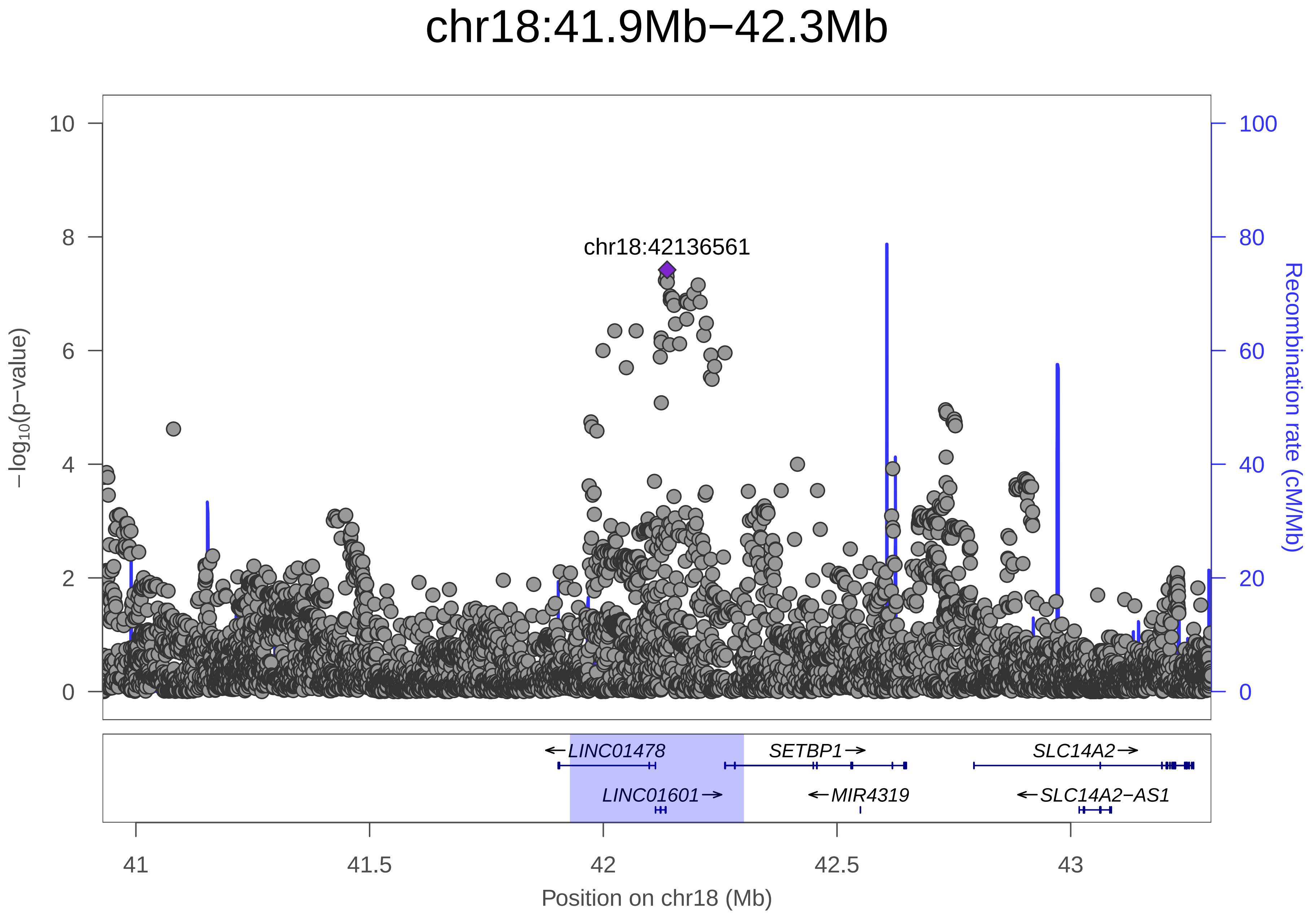
chr18:41.9Mb−42.3Mb
chr18:42136561
−log10(p−value)
Recombination rate (cM/Mb)
0
2
4
6
8
10
0
20
40
60
80
100
LINC01478
SETBP1
SLC14A2
LINC01601
MIR4319
SLC14A2−AS1
41
41.5
42
42.5
43
Position on chr18 (Mb)

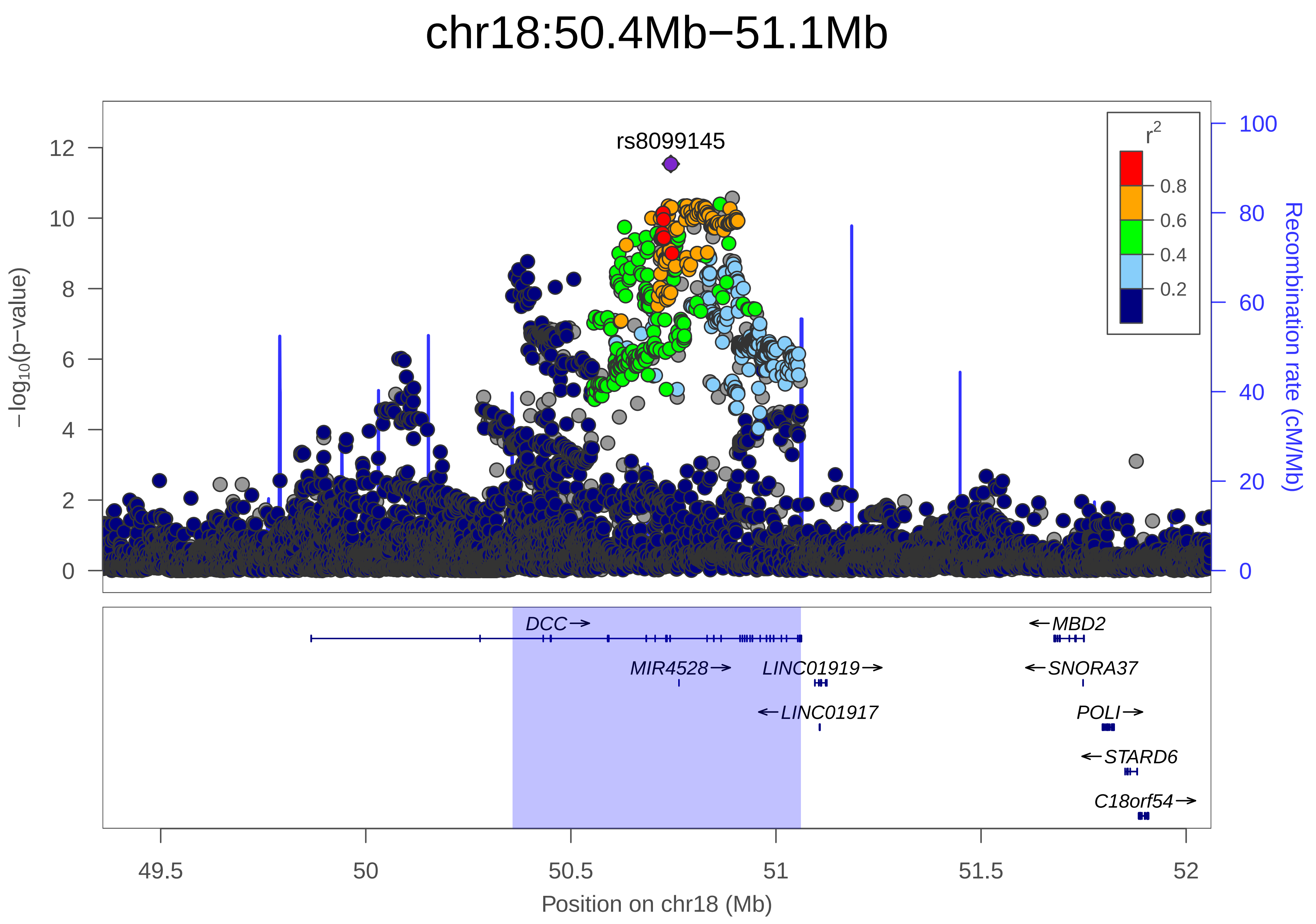

chr18:50.4Mb−51.1Mb
rs8099145
r2
0.8
0.6
0.4
0.2
−log10(p−value)
Recombination rate (cM/Mb)
0
2
4
6
8
10
12
0
20
40
60
80
100
DCC→
MIR4528→
LINC01919→
←LINC01917
←MBD2
←SNORA37
POLI→
←STARD6
C18orf54→
49.5
50
50.5
51
51.5
52
Position on chr18 (Mb)

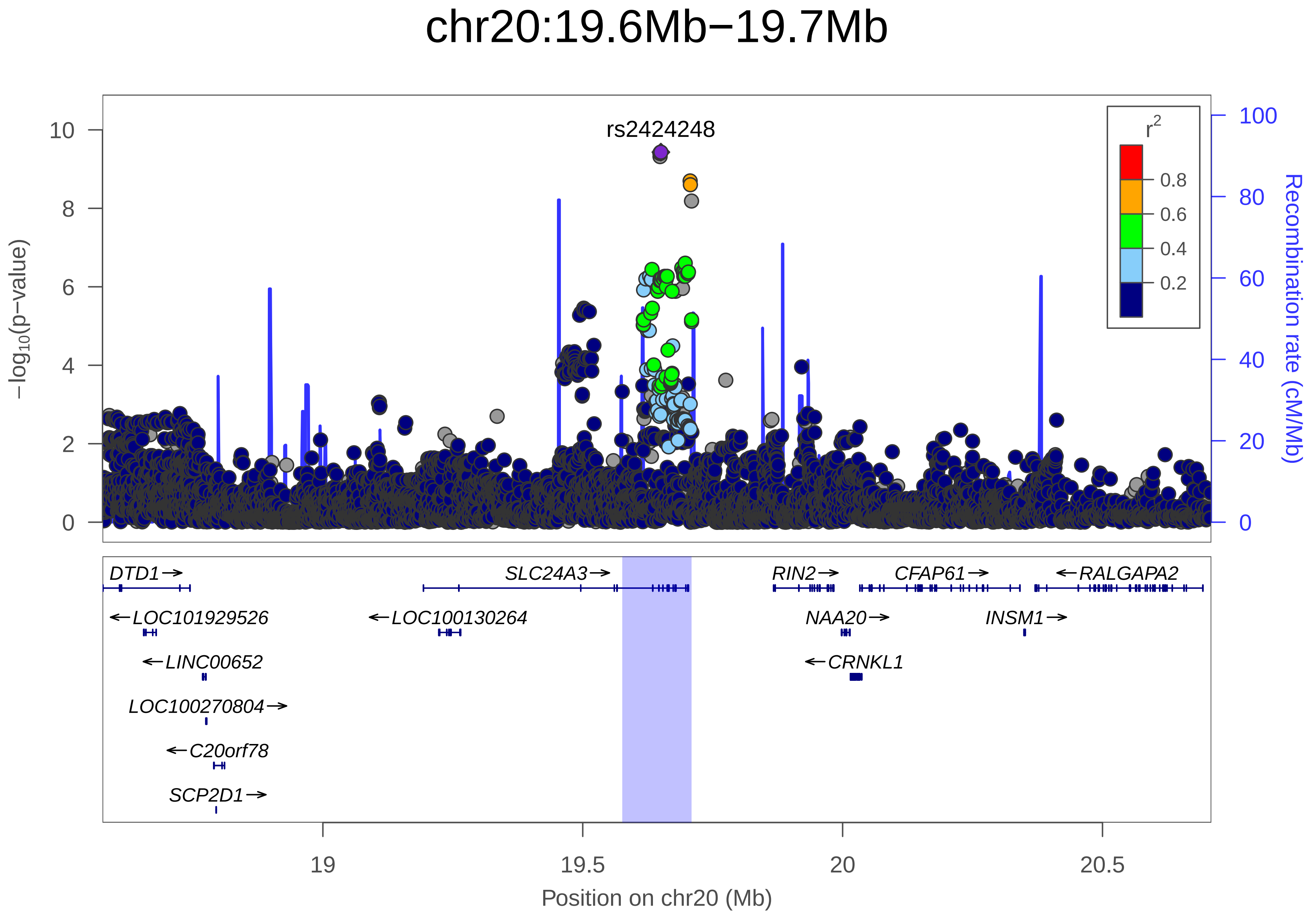
chr20:19.6Mb−19.7Mb
rs2424248
−log10(p−value)
Recombination rate (cM/Mb)
r2
0.8
0.6
0.4
0.2
0
2
4
6
8
10
0
20
40
60
80
100
DTD1→
SLC24A3→
RIN2→
CFAP61→
←RALGAPA2
←LOC101929526
←LOC100130264
NAA20→
INSM1→
←LINC00652
←CRNKL1
LOC100270804→
←C20orf78
SCP2D1→
19
19.5
20
20.5
Position on chr20 (Mb)

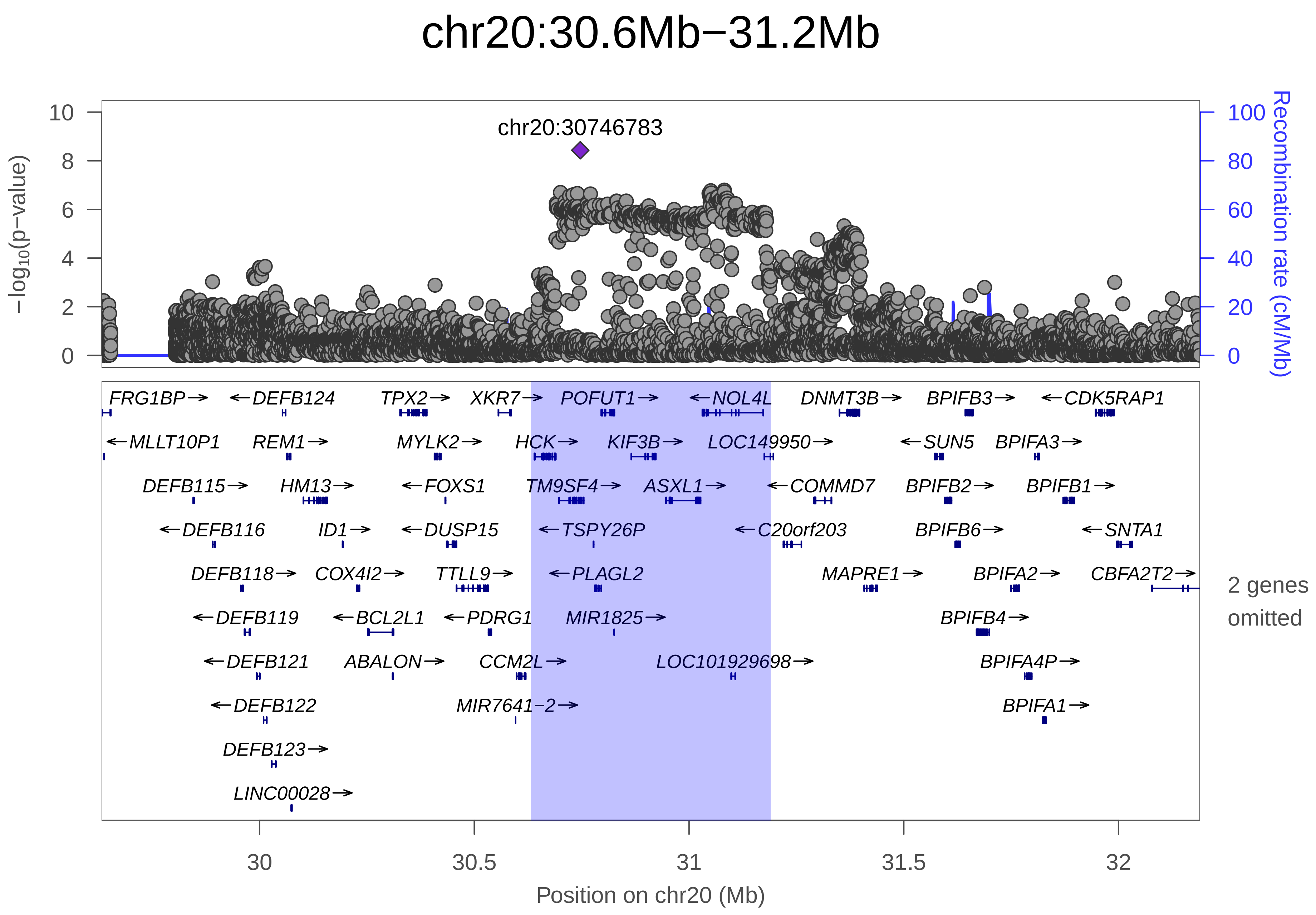
chr20:30.6Mb−31.2Mb
chr20:30746783
−log10(p−value)
Recombination rate (cM/Mb)
FRG1BP→
←DEFB124
TPX2→
XKR7→
POFUT1→
←NOL4L
DNMT3B→
BPIFB3→
←CDK5RAP1
←MLLT10P1
REM1→
MYLK2→
HCK→
KIF3B→
LOC149950→
←SUN5
BPIFA3→
DEFB115→
HM13→
←FOXS1
TM9SF4→
ASXL1→
←COMMD7
BPIFB2→
BPIFB1→
←DEFB116
ID1→
←DUSP15
←TSPY26P
←C20orf203
BPIFB6→
←SNTA1
DEFB118→
COX4I2→
TTLL9→
←PLAGL2
MAPRE1→
BPIFA2→
CBFA2T2→
2 genes omitted
←DEFB119
←BCL2L1
←PDRG1
MIR1825→
BPIFB4→
←DEFB121
ABALON→
CCM2L→
LOC101929698→
BPIFA4P→
←DEFB122
MIR7641−2→
BPIFA1→
DEFB123→
LINC00028→
Position on chr20 (Mb)
