# Supplementary material for: Genome-wide association study of multisite chronic pain in UK Biobank
Source: PLoS Genet. 2019 Jun 13;15(6):e1008164. doi: 10.1371/journal.pgen.1008164 (PMC6592570; doi:10.1371/journal.pgen.1008164)
Supplement: S1 Table — Six different regression models fitted during MR-RAPS analysis and their corresponding S1 or S2 Figs label (A-F) are shown. L2 = L2 loss function, huber = Huber loss function, tukey = Tukey loss function. (PDF) [file pgen.1008164.s008.pdf]

Supplementary Table 1: Model Types Fitted by MR-RAPS

Overdispersion	Loss Function	Underlying Model	Results-Section Figure Label
**FALSE**	**l2**	**No systematic pleiotropy, no idiosyncratic pleiotropy**	**A**
**FALSE**	**huber**	**No systematic pleiotropy, idiosyncratic pleiotropy**	**B**
**FALSE**	**tukey**	**No systematic pleiotropy, idiosyncratic pleiotropy**	**C**
**TRUE**	**l2**	**systematic pleiotropy, no idiosyncratic pleiotropy**	**D**
**TRUE**	**huber**	**systematic pleiotropy, idiosyncratic pleiotropy**	**E**
**TRUE**	**tukey**	**systematic pleiotropy, idiosyncratic pleiotropy**	**F**
