# Supplementary material for: Genome-wide association study of multisite chronic pain in UK Biobank
Source: PLoS Genet. 2019 Jun 13;15(6):e1008164. doi: 10.1371/journal.pgen.1008164 (PMC6592570; doi:10.1371/journal.pgen.1008164)
Supplement: S2 Table — Genes of interest as determined via Supplementary Methods. Note that this is distinct from MAGMA gene-based test results (N significant genes there = 113). (DOCX) [file pgen.1008164.s009.docx]

| **Gene Symbol** | **Gene Name** |
| --- | --- |
| *DCC* | Deleted in Colorectal Cancer |
| *SDK1* | Sidekick Cell Adhesion Molecule 1 |
| *MAML3* | Mastermind Like Transcriptional Coactivator 3 |
| *FOXP2* | Forkhead Box P2 |
| *GABRB2* | Gamma-Aminobutyric Acid Receptor, Beta-2 |
| *ASTN2* | Astrotactin 2 |
| *EXD3* | Exonuclease 3’-5’ Domain Containing 3 |
| *MON1A* | MON1 Homolog A, Secretory Trafficking Associated |
| *ECM1* | Extracellular Matrix Protein 1 |
| *FAM120A* | Family With Sequence Similarity 120A |
| *FAF1* | Fas Associated Factor 1 |
| *KCND3* | Potassium Voltage-Gated Channel Subfamily D Member 3 |
| *CTNNA2* | Catenin Alpha 2 |
| *GMPPB* | GDP-Mannose Pyrophosphorylase B |
| *BBX* | BBX, HMG-Box Containing |
| *STAG1* | Stromal Antigen 1 |
| *ANAPC4* | Anaphase Promoting Complex Subunit 4 |
| *SLC39A8* | Solute Carrier Family 39 Member 8 |
| *CEP120* | Centrosomal Protein 120 |
| *UTRN* | Utrophin |
| *SP4* | Sp4 Transcription Factor |
| *DYNC1I1* | Dynein Cytoplasmic 1 Intermediate Chain 1 |
| *Y_RNA* | RNA, Ro-Associated Y3 |
| *MLLT10* | MLLT10, Histone Lysine Methyltransferase DOT1L Cofactor |
| *SORCS3* | Sortilin Related VPS10 Domain Containing Receptor 3 |
| *KNDC1* | Kinase Non-Catalytic C-Lobe Domain Containing 1 |
| *SOX6* | SRY-Box 6 |
| *NUMB* | NUMB, Endocytic Adaptor Protein |
| *PRC1* | Protein Regulator Of Cytokinesis 1 |
| *NMT1* | N-Myristoyltransferase 1 |
| *SLC24A3* | Solute Carrier Family 24 Member 3 |
| *MLN* | Motilin |
| *CA10* | Carbonic Anhydrase 10 |
| *MON1B* | MON1 Homolog B, Secretory Trafficking Associated |

Genes of interest as determined via Supplementary Methods. Note that this is distinct from MAGMA gene-based test results (N significant genes there = 143).
