# Supplementary material for: Genome-wide association study of multisite chronic pain in UK Biobank
Source: PLoS Genet. 2019 Jun 13;15(6):e1008164. doi: 10.1371/journal.pgen.1008164 (PMC6592570; doi:10.1371/journal.pgen.1008164)
Supplement: S3 Table — (DOCX) [file pgen.1008164.s010.docx]

| Trait | Rg | Se | Z | H2 | P_h2_ (FDR) | Source | PMID | Category | P | P (FDR) |
| --- | --- | --- | --- | --- | --- | --- | --- | --- | --- | --- |
| Inflammatory Bowel Disease (European Ancestry) | 0.05 | 0.03 | 1.75 | 0.333 | 9.17E-21 | ld_hub | 26192919 | autoimmune | 0.08 | 0.1101 |
| Celiac disease | -0.07 | 0.05 | -1.49 | 0.314 | 2.50E-10 | ld_hub | 20190752 | autoimmune | 0.136 | 0.1756 |
| Crohn’s disease | 0.04 | 0.03 | 1.35 | 0.504 | 2.65E-17 | ld_hub | 26192919 | autoimmune | 0.179 | 0.2125 |
| Systemic lupus erythematosus | 0.06 | 0.04 | 1.33 | 0.390 | 9.77E-09 | ld_hub | 26502338 | autoimmune | 0.184 | 0.2125 |
| Ulcerative colitis | 0.04 | 0.04 | 1.08 | 0.257 | 1.19E-14 | ld_hub | 26192919 | autoimmune | 0.281 | 0.3094 |
| Bipolar disorder | -0.02 | 0.04 | -0.66 | 0.436 | 5.51E-29 | ld_hub | 21926972 | psychiatric | 0.509 | 0.5329 |
| Parkinson’s disease | 0 | 0.04 | 0.05 | 0.409 | 0.000761 | ld_hub | 19915575 | neurological | 0.961 | 0.9612 |

Non-significant genetic correlation results. rg = genetic correlation coefficient value, se = standard error of correlation value, z = z value, h2 = SNP-heritability value, ph2(fdr) = p value (FDR-corrected) for SNP-heritability, source = source of GWAS summary statistics, PMID = PubMed ID of associated paper (if applicable), p = p value for genetic correlation coefficient, p(fdr) = FDR-corrected p value for genetic correlation coefficient.
