# Supplementary material for: Genome-wide association study of multisite chronic pain in UK Biobank
Source: PLoS Genet. 2019 Jun 13;15(6):e1008164. doi: 10.1371/journal.pgen.1008164 (PMC6592570; doi:10.1371/journal.pgen.1008164)
Supplement: S4 Table — MR results for MDD-exposure. Β refers to the causal effect, SE (β) and P (β) to the standard error and p value of β, P (AD) to the Anderson-Darling test of normality p value, P (SW) to the Shapiro-Wilk test of normality p value, tau to the over-dispersion statistic size and P (τ) to the p value. C.F = corresponding QQ plot panel for the model. P (τ) was calculated from the tau estimate and its standard error [139]. The row of the table corresponding to the regression model found to be best-fitting is in bold. (DOCX) [file pgen.1008164.s011.docx]

| overdispersion | Loss function | β | SE (β) | P (β) | P (AD) | P (SW) | τ | P (τ) | C.F |
| --- | --- | --- | --- | --- | --- | --- | --- | --- | --- |
| FALSE | L2 | 0.0117 | 0.0052 | 0.0241 | 0.9375 | 5.34E-01 | NA | NA | Fig 7. A |
| FALSE | Huber | 0.0153 | 0.0054 | 0.0042 | 0.9285 | 5.23E-01 | NA | NA | Fig 7. B |
| **FALSE** | **Tukey** | **0.0185** | **0.0054** | **0.0006** | **0.9230** | **5.18E-01** | **NA** | **NA** | Fig 7. C |
| TRUE | L2 | -0.0096 | 0.0132 | 0.4671 | 0.0080 | 1.76E-03 | 1.61E-04 | 0.0470 | Fig 7. D |
| TRUE | Huber | -0.0056 | 0.0126 | 0.6556 | 0.0087 | 2.11E-03 | 1.30E-04 | 0.0677 | Fig 7. E |
| TRUE | Tukey | -0.0065 | 0.0137 | 0.6330 | 0.0055 | 9.03E-04 | 1.67E-04 | 0.0627 | Fig 7. F |

MR results for MDD-exposure. Β refers to the causal effect, SE (β) and P (β) to the standard error and p value of β, P (AD) to the Anderson-Darling test of normality p value, P (SW) to the Shapiro-Wilk test of normality p value, tau to the over-dispersion statistic size and P (τ) to the p value. C.F = corresponding QQ plot panel for the model. P (τ) was calculated from the tau estimate and its standard error (28). The row of the table corresponding to the regression model found to be best-fitting is in bold.
