# Supplementary material for: Genome-wide association study of multisite chronic pain in UK Biobank
Source: PLoS Genet. 2019 Jun 13;15(6):e1008164. doi: 10.1371/journal.pgen.1008164 (PMC6592570; doi:10.1371/journal.pgen.1008164)
Supplement: S5 Table — MR results for chronic pain-exposure. Β refers to the causal effect, SE (β) and P (β) to the standard error and p value of β, P (AD) to the Anderson-Darling test of normality p value, P (SW) to the Shapiro-Wilk test of normality p value, τ to the over-dispersion statistic size and P (τ) to the p value. P (τ) was calculated from the τ estimate and its standard error [139]The row of the table corresponding to the regression model found to be of best fit is in bold. (DOCX) [file pgen.1008164.s012.docx]

| overdispersion | Loss function | β | SE (β) | P (β) | P (AD) | P (SW) | τ | P (τ) | C.F |
| --- | --- | --- | --- | --- | --- | --- | --- | --- | --- |
| FALSE | L2 | 0.1714 | 0.0605 | 0.0046 | 0.4256 | 0.0853 | NA | NA | Fig 8. A |
| FALSE | Huber | 0.1815 | 0.0621 | 0.0034 | 0.4247 | 0.0835 | NA | NA | Fig 8. B |
| FALSE | Tukey | 0.2097 | 0.0621 | 0.0007 | 0.4221 | 0.0784 | NA | NA | Fig 8. C |
| TRUE | L2 | 0.1201 | 0.0790 | 0.1286 | 0.8374 | 0.2853 | 9.81E-05 | 2.43E-03 | Fig 8. D |
| TRUE | Huber | 0.1446 | 0.0801 | 0.0712 | 0.8289 | 0.2724 | 9.18E-05 | 5.13E-03 | Fig 8. E |
| **TRUE** | **Tukey** | **0.1578** | **0.0795** | **0.0471** | **0.8236** | **0.2641** | **8.77E-05** | **7.09E-03** | Fig 8. F |

MR results for chronic pain-exposure. Β refers to the causal effect, SE (β) and P (β) to the standard error and p value of β, P (AD) to the Anderson-Darling test of normality p value, P (SW) to the Shapiro-Wilk test of normality p value, τ to the over-dispersion statistic size and P (τ) to the p value. P (τ) was calculated from the τ estimate and its standard error (28)The row of the table corresponding to the regression model found to be of best fit is in bold.
