# Supplementary material for: Genome-wide association study of multisite chronic pain in UK Biobank
Source: PLoS Genet. 2019 Jun 13;15(6):e1008164. doi: 10.1371/journal.pgen.1008164 (PMC6592570; doi:10.1371/journal.pgen.1008164)
Supplement: S6 Table — Regression beta coefficient values (Estimate), odds ratios (OR), and P values. The reference level for ‘sex’ is set to female, PRS = z-polygenic risk score. (DOCX) [file pgen.1008164.s013.docx]

| **Term** | **Estimate** | **SE (Estimate)** | **Z** | **P** | **OR** |
| --- | --- | --- | --- | --- | --- |
| (Intercept) | -61.418 | 2.763 | -22.227 | 1.90E-109 | 2.12E-27 |
| Age | 0.016 | 0.002 | 7.451 | 9.25E-14 | 1.02 |
| Sex | -0.488 | 0.035 | -14.073 | 5.56E-45 | 0.61 |
| PRS | 0.488 | 0.022 | 22.239 | 1.45E-109 | 1.63 |

Regression beta coefficient values (Estimate), odds ratios (OR), and P values. The reference level for ‘sex’ is set to female, PRS = z-polygenic risk score.
