# Supplementary material for: Genome-wide association study of multisite chronic pain in UK Biobank
Source: PLoS Genet. 2019 Jun 13;15(6):e1008164. doi: 10.1371/journal.pgen.1008164 (PMC6592570; doi:10.1371/journal.pgen.1008164)
Supplement: S7 Table — GL = Genomic Locus, Chr = chromosome, pos = position, base pairs, mcp_A2 = other allele (MCP GWAS), mcp_A1 = effect allele (MCP GWAS), mcp_beta = effect (beta) (MCP GWAS), mcp_se = standard error of beta (MCP GWAS), cwp_A1 = effect allele (CWP GWAS), cwp_A2 = other allele (CWP GWAS), cwp_beta = effect (beta) (CWP GWAS), cwp_se = standard error of the beta (CWP GWAS), cwp_gwas_p = gwas P value (CWP GWAS). (DOCX) [file pgen.1008164.s014.docx]

| GL | rsID | chr | pos | p | mcp_A2 | mcp_A1 | mcp_gwas_P | mcp_beta | mcp_se | cwp_A1 | cwp_A2 | cwp_beta | cwp_se | cwp_gwas_P |
| --- | --- | --- | --- | --- | --- | --- | --- | --- | --- | --- | --- | --- | --- | --- |
| 1 | rs10888692 | 1 | 50991473 | 5.30E-09 | G | C | 5.30E-09 | -0.0143259 | 0.0024546 | C | G | -0.00669064 | 0.0053948 | 0.21 |
| 1 | rs35311109 | 1 | 51478336 | 1.80E-08 | A | T | 1.80E-08 | -0.0145472 | 0.0025824 | T | A | -0.00517 | 0.0056789 | 0.36 |
| 2 | rs197422 | 1 | 1.12E+08 | 2.00E-09 | A | C | 2.00E-09 | -0.0150195 | 0.0025027 | C | A | 0.000405999 | 0.0055171 | 0.94 |
| 2 | rs12033257 | 1 | 1.12E+08 | 3.10E-08 | G | A | 3.10E-08 | 0.0139379 | 0.0025186 | A | G | -0.00237042 | 0.00556 | 0.67 |
| 3 | rs59898460 | 1 | 1.5E+08 | 9.20E-12 | C | T | 9.20E-12 | 0.0168835 | 0.0024764 | T | C | 0.00451385 | 0.0054531 | 0.41 |
| 4 | rs12071912 | 1 | 2.43E+08 | 5.30E-09 | T | C | 5.30E-09 | -0.0153198 | 0.0026241 | C | T | -0.0125705 | 0.005799 | 0.03 |
| 4 | rs12058508 | 1 | 2.43E+08 | 1.20E-08 | A | G | 1.20E-08 | -0.015889 | 0.0027846 | G | A | -0.0115768 | 0.0061656 | 0.06 |
| 5 | rs4852567 | 2 | 80703379 | 4.30E-08 | G | A | 4.30E-08 | 0.0148524 | 0.0027109 | A | G | 0.00692484 | 0.0059315 | 0.24 |
| 6 | rs35713889 | 3 | 49162583 | 3.60E-08 | T | C | 3.60E-08 | -0.032398 | 0.0058784 | C | T | -0.00302753 | 0.0129788 | 0.82 |
| 6 | rs7628207 | 3 | 49754970 | 8.40E-10 | C | T | 8.40E-10 | 0.0195319 | 0.0031824 | T | C | 0.020242 | 0.0068909 | 0.0033 |
| 6 | rs62260755 | 3 | 49898318 | 5.50E-09 | G | C | 5.50E-09 | -0.0170906 | 0.0029311 | C | G | -0.0170657 | 0.0064135 | 0.0078 |
| 6 | rs9831967 | 3 | 50217234 | 9.50E-09 | T | C | 9.50E-09 | 0.0140282 | 0.0024443 | C | T | 0.0151083 | 0.0053771 | 0.005 |
| 7 | rs28428925 | 3 | 1.07E+08 | 1.40E-09 | A | G | 1.40E-09 | -0.0214355 | 0.0035411 | G | A | -0.0165719 | 0.0077826 | 0.033 |
| 7 | rs638293 | 3 | 1.07E+08 | 4.90E-08 | T | A | 4.90E-08 | 0.0145921 | 0.0026742 | A | T | 0.00955135 | 0.0059239 | 0.11 |
| 8 | rs6770476 | 3 | 1.36E+08 | 9.40E-09 | T | C | 9.40E-09 | -0.0153908 | 0.0026809 | C | T | -0.00724833 | 0.0059496 | 0.22 |
| 9 | rs34811474 | 4 | 25408838 | 2.70E-11 | A | G | 2.70E-11 | 0.0191705 | 0.0028781 | G | A | 0.0163473 | 0.0063152 | 0.0096 |
| 10 | rs17199964 | 4 | 1.03E+08 | 4.10E-08 | A | G | 4.10E-08 | -0.0247958 | 0.0045185 | G | A | -0.0069167 | 0.0098236 | 0.48 |
| 10 | rs34592089 | 4 | 1.03E+08 | 1.20E-10 | A | G | 1.20E-10 | -0.0326518 | 0.0050675 | G | A | -0.0157633 | 0.0109466 | 0.15 |
| 10 | rs13135092 | 4 | 1.03E+08 | 1.50E-13 | G | A | 1.50E-13 | -0.0327828 | 0.0044374 | A | G | -0.00994974 | 0.0096927 | 0.3 |
| 11 | rs13136239 | 4 | 1.41E+08 | 3.60E-08 | A | G | 3.60E-08 | 0.0141059 | 0.0025609 | G | A | 0.0192237 | 0.0056311 | 0.00064 |
| 12 | rs6869446 | 5 | 65570607 | 9.50E-09 | C | T | 9.50E-09 | -0.0143645 | 0.0025026 | T | C | -0.0011983 | 0.0055001 | 0.83 |
| 13 | rs1363103 | 5 | 1.04E+08 | 9.20E-09 | C | T | 9.20E-09 | 0.0144148 | 0.0025091 | T | C | 0.00658086 | 0.0055234 | 0.23 |
| 13 | rs40465 | 5 | 1.04E+08 | 9.10E-09 | G | T | 9.10E-09 | -0.0148694 | 0.0025877 | T | G | -0.00482418 | 0.0057045 | 0.4 |
| 13 | rs1976423 | 5 | 1.04E+08 | 8.20E-09 | C | A | 8.20E-09 | -0.0140182 | 0.002432 | A | C | -0.0041403 | 0.0053476 | 0.44 |
| 14 | rs17474406 | 5 | 1.23E+08 | 2.40E-08 | A | G | 2.40E-08 | -0.0491806 | 0.0088098 | G | A | -0.0043809 | 0.0194542 | 0.82 |
| 15 | rs1946247 | 5 | 1.61E+08 | 4.90E-08 | G | T | 4.90E-08 | -0.0190308 | 0.0034891 | T | G | -0.00992802 | 0.0076404 | 0.19 |
| 16 | rs4713656 | 6 | 33648997 | 1.50E-08 | A | G | 1.50E-08 | 0.0145713 | 0.0025743 | G | A | 0.00364521 | 0.0056638 | 0.52 |
| 16 | rs6915136 | 6 | 33651322 | 2.40E-08 | C | T | 2.40E-08 | -0.0182277 | 0.0032661 | T | C | 0.00679141 | 0.0072617 | 0.35 |
| 16 | rs482786 | 6 | 33707599 | 3.60E-09 | C | T | 3.60E-09 | -0.0162197 | 0.0027488 | T | C | -0.00439941 | 0.0061024 | 0.47 |
| 16 | rs4713675 | 6 | 33718418 | 8.30E-10 | T | C | 8.30E-10 | 0.0150329 | 0.0024489 | C | T | 0.00120694 | 0.0053873 | 0.82 |
| 16 | rs3828783 | 6 | 33767727 | 3.30E-08 | A | G | 3.30E-08 | 0.0173936 | 0.0031484 | G | A | 0.0123751 | 0.0069192 | 0.074 |
| 16 | rs767896 | 6 | 33791515 | 2.80E-09 | T | C | 2.80E-09 | 0.0157272 | 0.0026457 | C | T | 0.00502396 | 0.0058301 | 0.39 |
| 16 | rs11751591 | 6 | 33794215 | 2.70E-10 | A | G | 2.70E-10 | 0.0214053 | 0.0033896 | G | A | 0.0139265 | 0.0074519 | 0.062 |
| 16 | rs17600945 | 6 | 33802263 | 1.80E-09 | C | A | 1.80E-09 | 0.0149733 | 0.002491 | A | C | 0.00711997 | 0.005488 | 0.19 |
| 17 | rs205262 | 6 | 34563164 | 1.60E-08 | G | A | 1.60E-08 | -0.015494 | 0.0027442 | A | G | -0.00806896 | 0.0060198 | 0.18 |
| 17 | rs6907508 | 6 | 34592090 | 1.10E-08 | G | A | 1.10E-08 | -0.0217127 | 0.0037947 | A | G | -0.0172047 | 0.0083144 | 0.039 |
| 17 | rs79834165 | 6 | 34889423 | 3.40E-08 | C | T | 3.40E-08 | -0.0370699 | 0.0067152 | T | C | -0.0353 | 0.0147539 | 0.017 |
| 17 | rs33959228 | 6 | 35259397 | 1.30E-08 | T | C | 1.30E-08 | -0.0468654 | 0.0082325 | C | T | -0.0487607 | 0.0182443 | 0.0075 |
| 18 | rs6926377 | 6 | 1.45E+08 | 7.90E-09 | C | A | 7.90E-09 | -0.0154511 | 0.0026771 | A | C | 0.000279215 | 0.0058912 | 0.96 |
| 19 | rs10259354 | 7 | 3487414 | 3.00E-08 | A | G | 3.00E-08 | 0.0146793 | 0.0026492 | G | A | -0.00015617 | 0.005807 | 0.98 |
| 20 | rs7798894 | 7 | 21552995 | 1.60E-08 | T | A | 1.60E-08 | 0.015261 | 0.0026986 | A | T | 0.00623613 | 0.0059432 | 0.29 |
| 21 | rs6966540 | 7 | 95727967 | 3.30E-08 | C | T | 3.30E-08 | -0.0139057 | 0.0025164 | T | C | -0.00992351 | 0.0055501 | 0.074 |
| 21 | rs10156143 | 7 | 95844896 | 3.80E-08 | C | T | 3.80E-08 | -0.0139556 | 0.0025369 | T | C | -0.0133262 | 0.0055654 | 0.017 |
| 22 | rs2396719 | 7 | 1.14E+08 | 1.30E-08 | A | G | 1.30E-08 | -0.0160539 | 0.0028237 | G | A | -0.0123589 | 0.0061712 | 0.045 |
| 22 | rs12537376 | 7 | 1.14E+08 | 1.70E-09 | G | A | 1.70E-09 | 0.0150788 | 0.0025045 | A | G | 0.0075699 | 0.0055239 | 0.17 |
| 22 | rs1859100 | 7 | 1.14E+08 | 6.40E-09 | G | T | 6.40E-09 | -0.0144817 | 0.0024947 | T | G | -0.00741426 | 0.0054977 | 0.18 |
| 23 | rs11786084 | 8 | 1.43E+08 | 2.30E-08 | A | G | 2.30E-08 | -0.0144791 | 0.002591 | G | A | -0.00407364 | 0.0056597 | 0.47 |
| 24 | rs7870079 | 9 | 96161300 | 1.60E-08 | T | C | 1.60E-08 | 0.0138424 | 0.0024503 | C | T | 0.0100817 | 0.0053892 | 0.061 |
| 24 | rs10992729 | 9 | 96181075 | 1.10E-09 | T | C | 1.10E-09 | 0.0157618 | 0.0025876 | C | T | 0.0145295 | 0.0056988 | 0.011 |
| 24 | rs10821169 | 9 | 96361119 | 3.20E-08 | T | C | 3.20E-08 | 0.0137651 | 0.0024893 | C | T | 0.0134737 | 0.0054564 | 0.014 |
| 25 | rs6478241 | 9 | 1.19E+08 | 3.10E-09 | G | A | 3.10E-09 | 0.0149283 | 0.0025195 | A | G | 0.00269003 | 0.0055747 | 0.63 |
| 26 | 9:140241209_C_T | 9 | 1.4E+08 | 1.30E-08 | T | C | 1.30E-08 | -0.0267655 | 0.0047103 | C | T | -0.0283478 | 0.0102753 | 0.0058 |
| 26 | 9:140249826_C_T | 9 | 1.4E+08 | 1.30E-08 | T | C | 1.30E-08 | 0.0151261 | 0.0026587 | C | T | 0.0133267 | 0.0058416 | 0.023 |
| 26 | 9:140251458_G_A | 9 | 1.4E+08 | 5.30E-14 | A | G | 5.30E-14 | -0.0276759 | 0.0036783 | G | A | -0.0300154 | 0.0080625 | 2.00E-04 |
| 27 | rs2183271 | 10 | 21957229 | 3.10E-08 | C | T | 3.10E-08 | -0.0140394 | 0.0025369 | T | C | -0.00122744 | 0.0055595 | 0.83 |
| 28 | rs11599236 | 10 | 1.06E+08 | 3.30E-08 | C | T | 3.30E-08 | 0.0137892 | 0.002495 | T | C | 0.0103782 | 0.005517 | 0.06 |
| 29 | rs12765185 | 10 | 1.35E+08 | 3.90E-08 | A | T | 3.90E-08 | -0.0150848 | 0.0027461 | T | A | -0.00567564 | 0.0060237 | 0.35 |
| 30 | rs61883178 | 11 | 16317779 | 2.00E-10 | A | C | 2.00E-10 | -0.0207943 | 0.0032673 | C | A | -0.00257449 | 0.0072093 | 0.72 |
| 30 | rs7109139 | 11 | 16372241 | 2.00E-08 | C | T | 2.00E-08 | -0.015261 | 0.0027209 | T | C | -0.00783097 | 0.0059954 | 0.19 |
| 31 | rs2759694 | 13 | 53695378 | 3.90E-09 | G | A | 3.90E-09 | 0.0147735 | 0.0025095 | A | G | 0.00516741 | 0.005502 | 0.35 |
| 31 | rs6561715 | 13 | 53888526 | 1.80E-09 | A | T | 1.80E-09 | 0.0151705 | 0.0025233 | T | A | -0.00099263 | 0.0055152 | 0.86 |
| 31 | rs1443914 | 13 | 53917230 | 2.80E-11 | C | T | 2.80E-11 | 0.0162156 | 0.0024361 | T | C | 0.00727737 | 0.0053499 | 0.17 |
| 31 | rs7987501 | 13 | 53983445 | 2.50E-09 | C | G | 2.50E-09 | 0.0146558 | 0.0024586 | G | C | 0.0126468 | 0.0054233 | 0.02 |
| 31 | rs9527083 | 13 | 53991125 | 1.70E-10 | A | G | 1.70E-10 | 0.0166166 | 0.0026033 | G | A | 4.60E-05 | 0.0057248 | 0.99 |
| 32 | rs12435797 | 14 | 73797669 | 3.70E-08 | T | G | 3.70E-08 | -0.0172843 | 0.0031399 | G | T | -0.00887809 | 0.0069073 | 0.2 |
| 33 | rs2006281 | 14 | 1.04E+08 | 3.40E-08 | T | C | 3.40E-08 | 0.0134934 | 0.0024458 | C | T | 0.00256779 | 0.0053498 | 0.63 |
| 34 | rs2386584 | 15 | 91539572 | 2.80E-11 | G | T | 2.80E-11 | -0.0166445 | 0.0025001 | T | G | 0.00388569 | 0.0054828 | 0.48 |
| 35 | rs285026 | 16 | 77100089 | 1.90E-08 | T | G | 1.90E-08 | -0.0137932 | 0.0024549 | G | T | -0.00591689 | 0.0053942 | 0.27 |
| 36 | rs11871043 | 17 | 43172849 | 1.70E-09 | C | T | 1.70E-09 | 0.0148701 | 0.0024671 | T | C | 0.00594208 | 0.0054567 | 0.28 |
| 37 | rs11079993 | 17 | 50301552 | 5.70E-12 | T | G | 5.70E-12 | -0.017286 | 0.0025104 | G | T | -0.00477158 | 0.0055027 | 0.39 |
| 38 | rs35938312 | 18 | 50372821 | 2.90E-09 | A | G | 2.90E-09 | -0.0147018 | 0.0024766 | G | A | -0.00592337 | 0.0054144 | 0.27 |
| 38 | rs62099231 | 18 | 50724899 | 7.20E-11 | A | G | 7.20E-11 | -0.015851 | 0.0024328 | G | A | -0.0123089 | 0.00533 | 0.021 |
| 38 | rs62098013 | 18 | 50863861 | 4.00E-11 | A | G | 4.00E-11 | -0.0168595 | 0.0025524 | G | A | -0.0121196 | 0.0055942 | 0.03 |
| 38 | rs7226995 | 18 | 50895755 | 2.00E-09 | G | A | 2.00E-09 | -0.0146104 | 0.0024361 | A | G | -0.0159754 | 0.0053322 | 0.0027 |
| 38 | rs10164055 | 18 | 50919600 | 2.70E-08 | C | T | 2.70E-08 | -0.0136469 | 0.0024546 | T | C | -0.0124915 | 0.0053834 | 0.02 |
| 39 | rs2424248 | 20 | 19650324 | 3.70E-10 | A | G | 3.70E-10 | 0.0230287 | 0.0036755 | G | A | 0.00883328 | 0.008085 | 0.27 |

Association of top MCP-SNPs with CWP in UK Biobank.
